# Identification and validation of immune-related and inflammation-related genes in endometriosis

**DOI:** 10.3389/fendo.2025.1545670

**Published:** 2025-05-08

**Authors:** Yanzhen Zhou, Jingmin Li, Meihuan Chen, Hailong Huang

**Affiliations:** ^1^ College of Clinical Medicine for Obstetrics & Gynecology and Pediatrics, Fujian Medical University, Fuzhou, Fujian, China; ^2^ Fujian Maternity and Child Health Hospital, College of Clinical Medicine for Obstetrics & Gynecology and Pediatrics, Fujian Medical University, Fuzhou, Fujian, China

**Keywords:** endometriosis, immunity, inflammation, machine learning, biomarker

## Abstract

**Aim:**

Endometriosis is characterized by immune evasion and progressive inflammation. This study aimed to identify key genes related to immune and inflammation in endometriosis.

**Methods:**

Differentially expressed genes between patients with and without endometriosis were identified from the GEO database. Furthermore, immune- and inflammation-related genes (IRGs) were identified by intersecting the differentially expressed genes with known immune and inflammatory genes. Functional analyses of the GO and KEGG pathways of these genes were performed. Subsequently, three machine learning models—LASSO regression, SVM-RFE, and Boruta—were conducted to identify the potential key genes in endometriosis. Finally, the expressions of key genes in endometriosis were verified in two validation cohorts using an online database and qRT-PCR, and their immunoregulatory properties were verified.

**Results:**

A total of 13 differentially expressed IRGs were identified. Using machine learning algorithms, five key genes were selected in the endometriosis: BST2, IL4R, INHBA, PTGER2, and MET. Furthermore, the three hub genes exhibited consistent trends across both training and validation datasets. The three keys also correlated with infiltrated immune cells, checkpoint genes, and immune factors in various degrees. Finally, validation analysis using the online database and qRT-PCR confirmed that MET expression aligned with outcomes from both training and validation datasets.

**Conclusion:**

Three immune- and inflammation-related genes were identified as potential biomarkers of endometriosis, providing new insights into the molecular mechanisms underlying immune function in endometriosis. The immune-related function of MET, particularly its correlation with NK cell activity in endometriosis, will be the focus of future studies.

## 1 Introduction

Endometriosis(EM) is one of the most prevalent gynecological diseases, with a 10%-15% prevalence rate among women of reproductive age (1). It is estimated that endometriosis affects approximately 190 million women worldwide (2). It clinically manifests as severe pelvic pain and reduced fertility and is characterized by the presence and growth of endometrial tissue outside the uterus. This disease significantly impairs patients’ quality of life and imposes a heavy burden on the healthcare systems (3, 4). The progression of the disease is slow and often takes 7-10 years before the onset of symptoms, resulting in delays in diagnosis and optimal treatment (5). The pathogenesis of endometriosis remains poorly understood. Despite decades of research, noninvasive diagnostic markers for endometriosis are lacking, and no curative treatment is available. Therefore, it is imperative to identify the potential diagnostic biomarkers and molecular mechanisms of endometriosis to improve early diagnosis and treatment.

The widely accepted theory of endometriosis pathogenesis is a combination of retrograded menses and the immunosuppression hypothesis. Disturbances of the immune microenvironment are critical factors in the pathophysiology and development of endometriosis (6). Endometriosis is a chronic inflammatory disorder characterized by immune evasion and progressive inflammation (7). Inflammation resulting from immune dysregulation is the primary mechanism involved in cell proliferation and infiltration (8). Consequently, it is important to explore the genes associated with immune response and inflammation related to endometriosis.

Owing to the widespread adaptation of computer-based technology in the health sector and the subsequent availability of large health databases, disease prediction, and medical informatics have recently gained attention from the data science research community (9). Machine learning algorithms have also been extensively applied to screen genes associated with diseases (10, 11). Abnormal immune and inflammatory changes may be responsible for major symptoms of endometriosis and contribute to the development of endometriosis and the growth of endometrial tissue (12). Building on these characteristics, this study aims to identify novel and important genes as diagnostic markers and therapeutic targets with endometriosis by constructing and validating immune-related and inflammation-related genes (IRGs) using machine learning algorithms. We identified three critical genes and verified their potential as diagnostic markers. Furthermore, we analyzed the potential relationship between key genes and their immunoregulatory properties, demonstrating the significant role of these genes in the immunopathogenesis of endometriosis. Finally, MET’s downregulation in the online database and clinical samples from the endometriosis group versus the control group. In conclusion, this study offers a novel perspective for the diagnosis and treatment of endometriosis at the molecular level.

## 2 Materials and methods

### 2.1 Collection of tissue samples

This study was approved by the Medical and Ethics Committees of Fujian Maternity and Child Health Hospital (No. 2023KY140-KS001), and informed consent was obtained from each patient prior to enrollment. Ectopic endometrial tissue from two patients with broad ligament endometriosis, three patients with sacral ligament endometriosis, and five patients with ovarian endometriosis (all in the follicular phase, n = 10) (EM group) and 10 eutopic endometrial tissues from women (follicular phase,n = 10) with tubal factor infertility without endometriosis (control group). All 20 women underwent hysteroscopy and laparoscopy surgery at the Department of Obstetrics and Gynecology, Fujian Maternity and Child Health Hospital, between Oct 2023 to Jun 2024.

### 2.2 Data acquisition and processing

**Figure 1:**
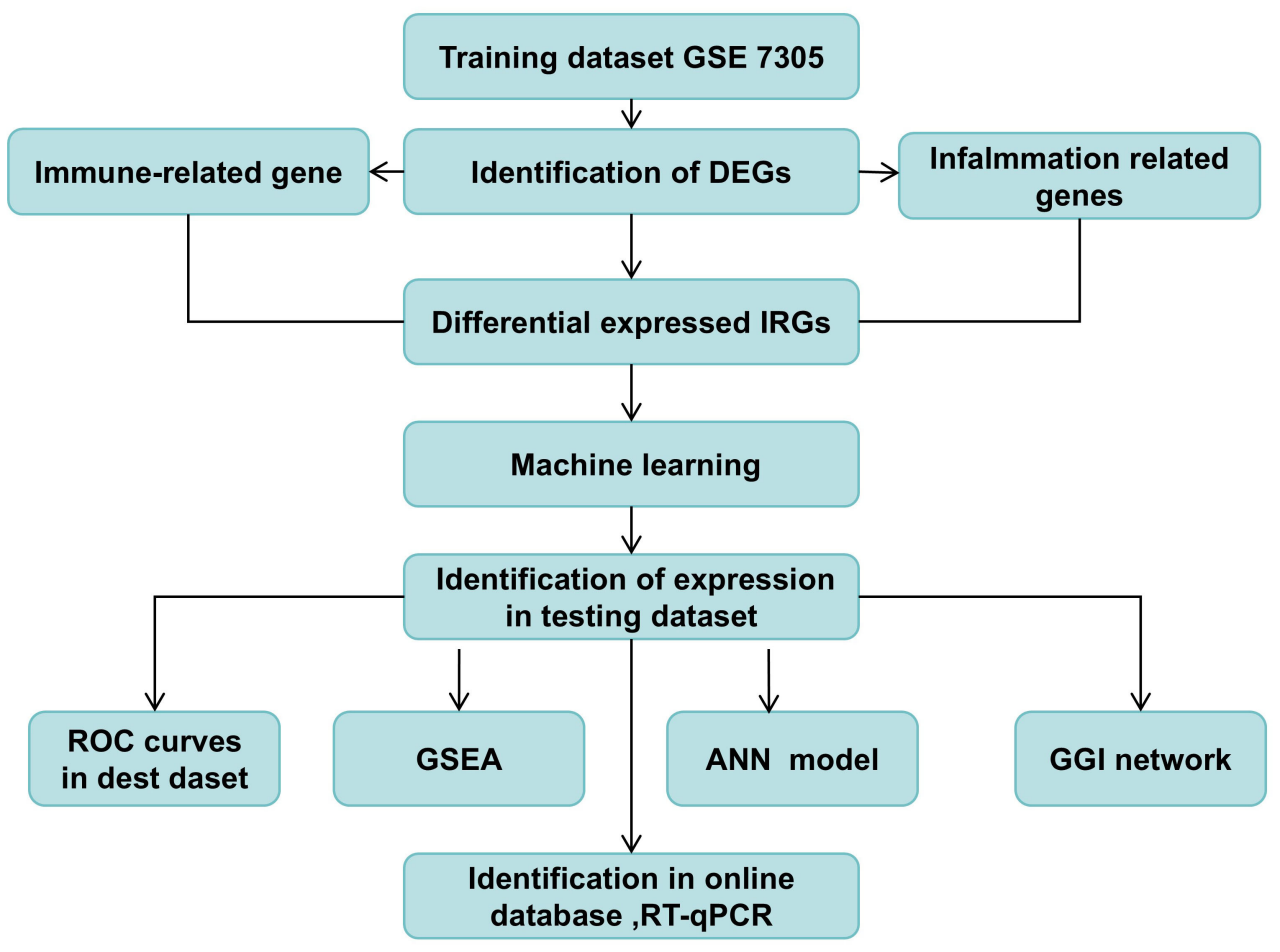
The study design. DEGs, differential expressed genes; ROC, Receiver Operator Characteristic; GSEA, Gene Set Enrichment Analysis; GGI, Gene-Gene Interaction Network; ANN, Construction of artificial neural network.

In this study, the gene expression datasets GSE7305, GSE23339, and GSE7307 were obtained from the GEO database (https://www.ncbi.nlm.nih.gov/geo/). The study design is illustrated in **Figure 1**. The training dataset GSE7305 comprised 10 samples without endometriosis and 10 samples with endometriosis, which were sequenced using GPL570 technology. The validation dataset GSE23339, comprising 9 non-endometriosis and 10 endometriosis samples, was sequenced using GPL6102. Additionally, GSE7307, which included 18 endometriosis and 18 non-endometriosis samples, was sequenced using GPL570.

Clinical information and sequencing data were obtained in compliance with GEO requirements. Data filtering, background correction, log2 transformation, and normalization were performed on the datasets.

### 2.3 Identification of differentially expressed IRGs

The differentially expressed genes (DEGs) between the endometriosis and non-endometriosis groups were analyzed using the LIMMA package in R Studio. Adj.P <0.05 and |log_2_FC| >1.0 were set to determine the significant differentially expressed genes in patients with endometriosis.

This study intersected the differentially expressed genes with immune-related genes and inflammatory-related genes as the differential expressed IRGs using the R package ‘ggVenndiagram ‘.

### 2.4 Functional enrichment analysis and construction of protein-protein interaction network

To identify specific biological pathways in IRGs, we conducted a comprehensive analysis. The R package ‘clusterProfler’ was used to perform Gene Ontology (GO) and Kyoto Encyclopedia of Genes and Genomes (KEGG) analyses to identify the functions and pathways. Visualization of the results was achieved using the R package ‘ggplot2’. GO annotations encompassed three main categories: biological processes (BP), cellular components (CC), and molecular functions (MF).

To construct the Protein-Protein Interaction (PPI) Network, the differentially expressed IRGs were simultaneously input into the STRING database (https://cn.string-db.org/), with the species defined as Homo sapiens and the PPI Network parameter set to 0.4 (medium confidence). Subsequently, Cytoscape (https://cytoscape.org/) was employed to visualize the protein-protein interaction network.

### 2.5 Machine learning and screening for potential key genes

To select disease diagnostic markers in the above IRGs, we developed three machine-learning models: LASSO regression, SVM-RFE, and Boruta. The potential key genes were determined by overlapping LASSO regression analysis, SVM analysis, and the Boruta algorithm results.

### 2.6 Identification and validation of the key genes

The validation datasets GSE23339 and GSE7307 were utilized to identify the expression trend of potential key genes. Furthermore, both the training and validation cohorts were utilized to evaluate the discriminative ability of hub genes in distinguishing endometriosis from non-endometriosis samples. The diagnostic performance of these hub genes was assessed by plotting the AUC using the R package ‘pROC’.

To further evaluate the predictive capability of the key genes, we developed a nomogram using the R package ‘rsm’ with the training set. The predictive performance of the nomogram model was assessed through calibration curves and decision curve analysis (DCA).

### 2.7 Gene-gene interaction networks and enrichment pathway analysis of key genes

GeneMANIA (https://genemania.org/) searches through extensive, publicly available biological datasets to identify functionally related genes. We constructed Gene–Gene Interaction (GGI) networks for key genes using the GeneMANIA platform and selected the top 20 most relevant genes as well as the seven most important pathways associated with the three key genes.

Gene Set Enrichment Analysis (GSEA) was performed for each key gene to explore the potential functions of the three key genes. First, we performed Spearman correlation analysis on the training set to assess the relationship between each key gene and all other genes using the R package ‘psych’. Then the enrichment analysis of GSEA was conducted using the R package ‘clusterProfiler’, based on the KEGG background gene set. The results were screened using the criteria |NES|>1 and p <0.05.

### 2.8 The construction and verification of the artificial neural network model

The training cohort was utilized to develop the Artificial Neural network (ANN) model, while the validation cohort was used for signature validation. The discrimination and net clinical benefit of the ANN model were assessed using a Receiver operating characteristic (ROC) curve.

### 2.9 Analysis of the relationship between key genes and immunoregulatory properties

#### 2.9.1 Immune cell infiltration pattern

To elucidate the differences in infiltrated immune cells between the control and EM samples, the training cohort was analyzed using ssGSE(single sample gene set enrichment analysis), yielding enrichment scores for 28 immune cells based on immune-related genes referenced in the literature (PMID:28052254). Spearman’s correlation analysis was conducted to investigate the relationship between hub gene expression and immune cell abundance using the ‘psych’ package in R Studio. The results were visualized using the R package ‘ggplot2’. Statistical significance was set at P <0.05.

#### 2.9.2 Correlation between key genes with immune checkpoints

This study involved a comparison of 53 immune checkpoints [PMID: 36979463, PMID: 36793711]. We analyzed differences in immune cell scores between the disease and control groups using the rank sum test and visualized the results with boxplots. Following this, we conducted Spearman’s correlation analysis to investigate the relationship between key genes and 18 differential immune checkpoints.

#### 2.9.3 Correlation with immune factors

The relationships between key genes and different immune factors, including 24 chemokines, 14 immunosuppressive factors, and 27 immunostimulatory factors, were evaluated using Spearman’s correlation analysis.

### 2.10 Validation of key genes in an online database (Turku Endometriosis Database)

The online Turku Endometriosis Database (https://endometdb.utu.fi) was used to validate the expression of five key genes in various tissues of patients with EM.

### 2.11 RNA isolation, reverse transcription, and real-time RT-PCR

Total RNA was extracted from the ectopic endometrial tissues of the EM group and endometrial tissues of the control group using TRIzol reagent (RNAprep Pure Tissue Kit, TIANGEN, Beijing, China) and was then reverse-transcribed into cDNA using the Primescript reverse transcription reagent kit (Takara, Dalian, China) following the manufacturer’s instructions.

Real-time PCR was performed using 2×SG Fast qPCR Master Mix (BBI, Roche, Switzerland) on a LightCycler480II Real-Time PCR System (Roche, Rotkeruz, Switzerland). A 10ul PCR reaction that included 1ul of cDNA, 5ul of sybrGreen qPCR Master Mix, and 0.2ul of each primer was prepared and adjusted to the final volume with double distilled H2O (ddH2O). b-actin was used as the internal control for each run. Reactions were performed according to the manufacturer’s protocol. The relative mRNA expression ratio was quantified using the 2 (-ΔΔCt) method. The Real-time RT-PCR primers used are listed below.

H-MET-F 5’ AATCTTGGGACATCAGAGGGT 3’

H-MET-R 5’ TAATGTATGCTCCACAATCACTTCT 3’

H-b-actin-F 5’ TAGTTGCGTTACACCCTTTCTTG 3’

H-b-actin-R 5’ TCACCTTCACCGTTCCAGTTT 3’

### 2.12 Western blotting

After extraction of total tissue proteins from RIPA lysates(Servicebio, Wuhan), protein concentrations were quantified using the BCA Protein Quantitative Assay Kit(Jabes Biotechnology Guangzhou). The proteins were denatured by boiling at 100°C for 10 min. Subsequently, the protein samples (40 ugs per well) were separated via electrophoresis on a 10% SDS-PAGE gel and transferred to a PVDF membrane(Millipore, USA). The membrane was incubated with a primary antibody rabbit anti-MET antibody(Abclonal Wuhan) and rabbit anti-β-actin(Affinity USA) at 4°C overnight). The next day, after washing the PVDF membrane three times with TBST (Servicebio Wuhan), it was incubated with an HRP-conjugated secondary antibody for 2 h at room temperature on an orbital shaker. Lastly, the membrane was exposed for imaging using Immobilon Western Chemiluminescent HRP Substrate (Servicebio, Wuhan). The development was carried out in a dark room, and the grayscale values of the target bands were analyzed after taking photographs.

### 2.13 Statistical analysis

Statistical analyses were conducted using Student’s and Mann-Whitney tests with Prism version 10.0 Graphpad software. A P-value of<0.05 was considered statistically significant.

## 3 Result

### 3.1 Identification of differentially expressed IRGs in EM

**Figure 2:**
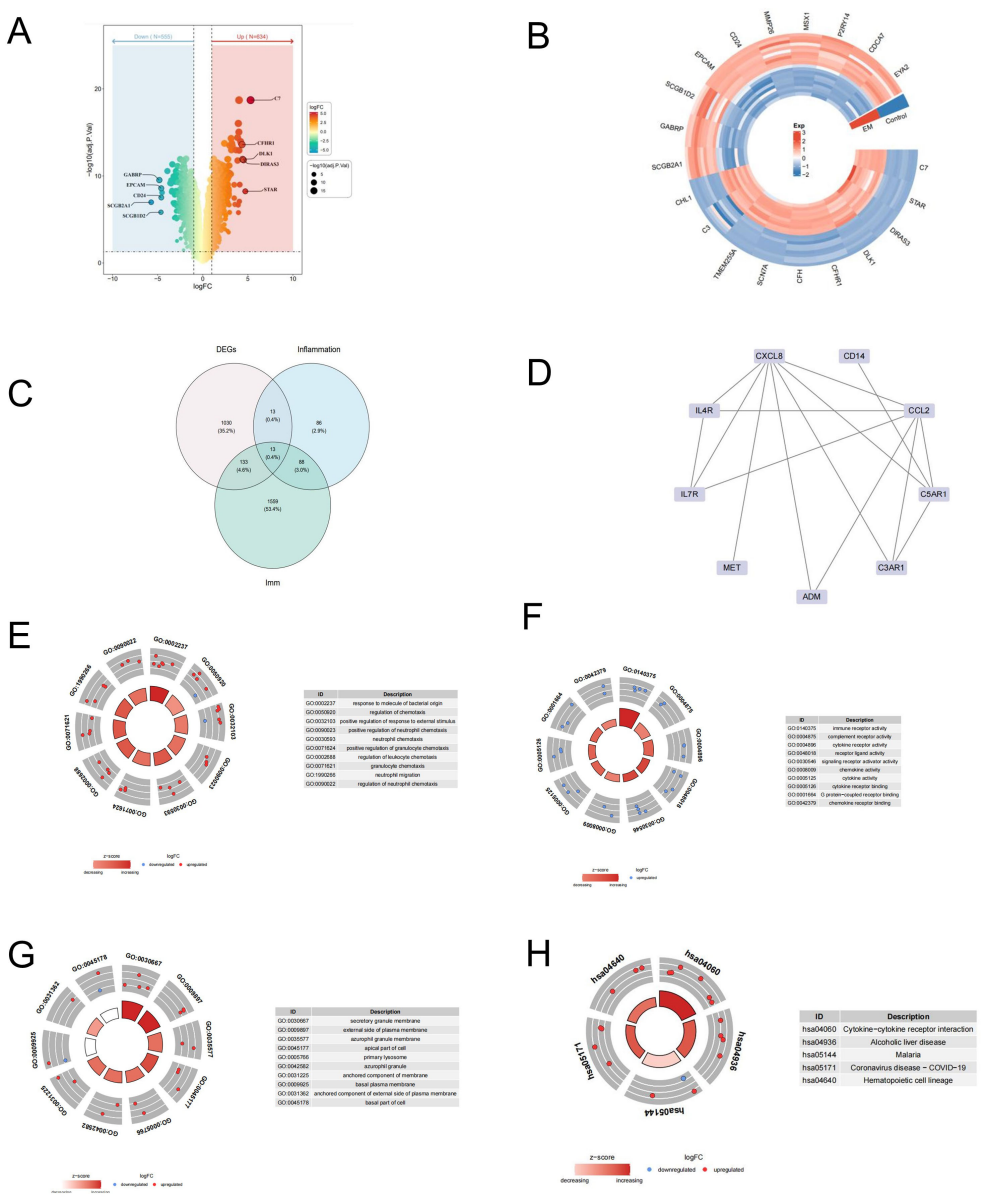
Identification of DEGs and functional enrichment analysis of differential expressed IRGS. **(A)** The heatmap and **(B)** volcano plot of differentially expressed genes (DEGs) between EM and control samples. **(C)** The venngram of DEGs and IRGs. **(D)** Analysis of PPI. **(E–H)** Gene Ontology (GO) and Kyoto Encyclopedia of Genes and Genomes (KEGG) functional enrichment analysis of differential expressed IRGs.

We screened 1189 differentially expressed genes(DEGs) between EM and control samples in the GSE7305 dataset, including 634 upregulated and 555 downregulated DEGs (**Supplementary Table S1**). Volcano and heat maps of the DEGs are shown in **Figures 2A, B**.

To screen differential expressed IRGs, we further overlapped DEGs and immune-related and inflammatory-related genes, and 13 IRGs were acquired, including BST2, C3AR1, IL4R, OSMR, INHBA, CXCL8, PTGER2, CCL2, CD14, ADM, IL7R, MET, and C5AR1 (**Figure 2C**).

### 3.2 Functional enrichment analysis of differential expressed IRGs

To investigate potential interactions among these genes, we performed the analysis of the protein-protein interaction (PPI) network involving 13 candidate genes. Fifteen interaction pairs, consisting of 9 genes, were identified. Refer to **Figure 2D**.

**Figure 3:**
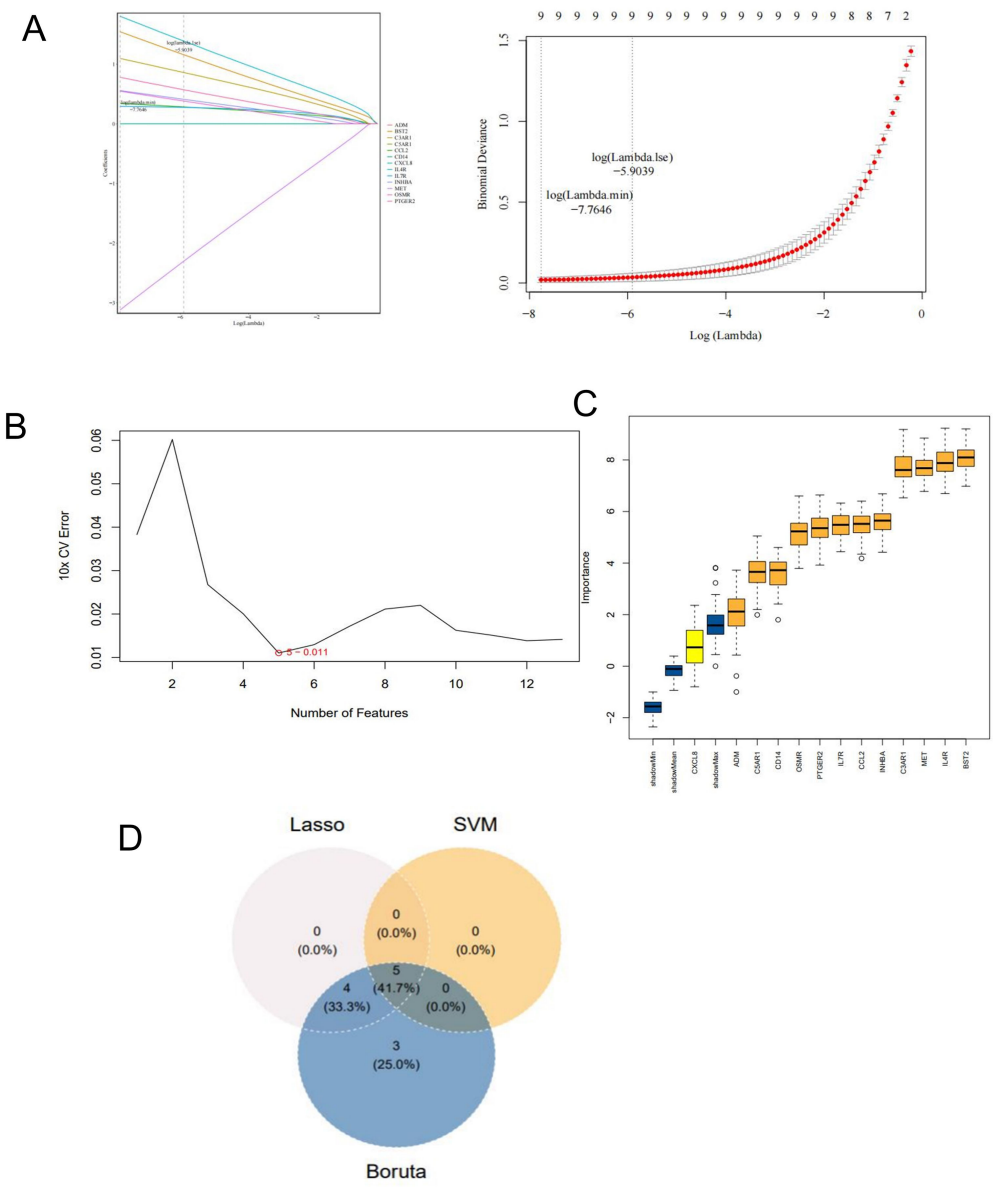
Screen for potential key genes. **(A)** Genes identifed by the LASSO regression analysis. **(B)** Optimal biomarker selection using the SVM-RFE analysis. **(C)** The 12 genes selected by the Boruta algorithm. **(D)** Venn diagram showing the potential key genes intersected by LASSO, SVM, and Boruta.

Subsequently, we annotated the 13 genes using the KEGG pathway and GO function to investigate the biological significance of each gene. A total of 217 Gene Ontologies (GOs) and 24 Kyoto Encyclopedia of Genes and Genomes (KEGG) pathways were enriched, as indicated in **Supplementary Tables S2, S3**. The primary enriched terms in the GO-CC category were ‘secretory granule membrane’, ‘external side of plasma membrane’, and ‘azurophil granule membrane’. In the GO-BP category, the top term included ‘response to molecules of bacterial origin’, ‘regulation of chemotaxis’, ‘positive regulation of response to external stimulus’, and ‘neutrophil chemotaxis’ (**Figure 3B**; P<0.05). Additionally, the GO-MF categories were predominantly enriched in ‘immune receptor activity’, ‘complement receptor activity’, and ‘cytokine receptor activity’ (**Figures 2E–G**; P<0.05). To further determine the potential signaling pathway, we analyzed the KEGG pathway. As **Figures 2E-H** indicated the IRGs were significantly enriched in several KEGG pathways, such as ‘cytokine-cytokine receptor interaction,’ ‘Alcoholic liver disease,’ and ‘Malaria,’ among others. These results indicate that certain IRGs may play a role in the immune response.

### 3.3 Identification of key genes

We use three machine learning models to screen potential hug genes. LASSO regression analysis, SVM-RFE analysis, and the Boruta algorithm were employed to identify key genes in the training dataset (**Figures 3A–C**). We screened meaningful five key genes: BST2, IL4R, INHBA, PTGER2, and MET. These genes resulted from the intersection of 9 genes from LASSO, 5 genes from SVM-RFE, and 12 genes from Boruta (**Figure 3D**).

### 3.4 Validation of the key genes

**Figure 4:**
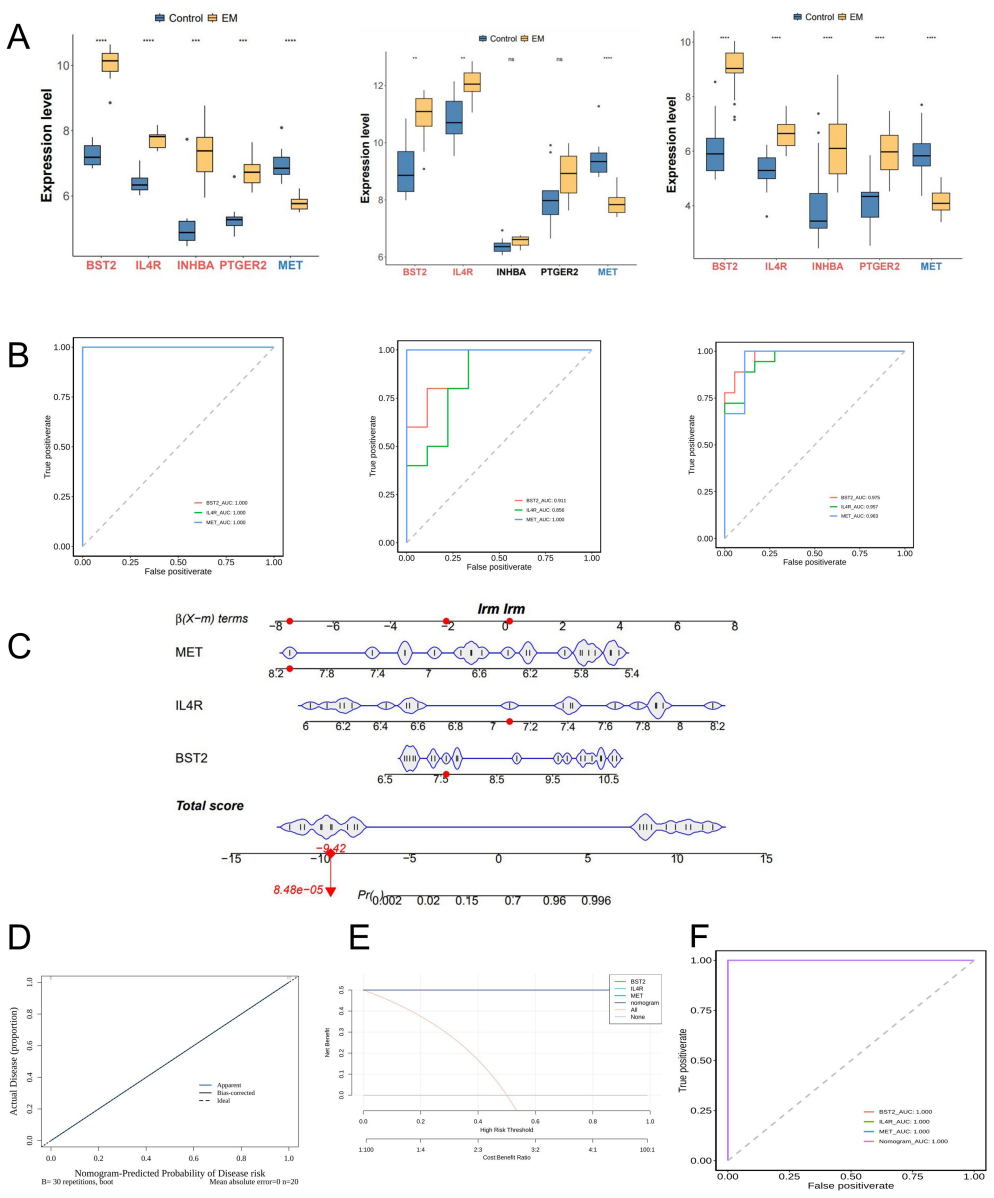
Identification and Validation of key genes. **(A)** Expression profiles of 5 potential key genes between EM and control group in the training and 2 test cohorts. **(B)** Receiver operating characteristics (ROC) curves of 3 key genes in training and test cohorts. **(C)** Nomogram displaying the predicted risk for EM based on the KEY genes. **(D)** Calibration curve showing the predicted performance of the nomogram. **(E)** DCA showing the clinical benefits of the nomogram. **(F)** ROC curves showing the diagnostic performance of the feature genes.

Two validation datasets were used to confirm the differential expression of the hub genes and to demonstrate their diagnostic capacity for endometriosis (**Figure 4A**). The three hub genes exhibited consistent trends across both training and validation datasets. Two genes exhibited an up-regulated trend, while one gene displayed a down-regulated trend in the endometriosis groups across both the training and validation databases.

The three genes were identified as key targets and their ability to differentiate between EM samples and control samples was evaluated using ROC analysis. The area under the curve (AUC) values of the three key genes were greater than 0.8 in both the training and validation sets, indicating their strong diagnostic value in EM (**Figure 4B**).

We also developed a nomogram model to assess the predictive ability of key genes. The calibration curve indicated the high accuracy of the nomogram model in predicting EM (**Figure 4C**). DCA indicated potential benefits for patients utilizing the nomogram model, showing a greater clinical advantage observed compared to the single gene curve (**Figure 4F**). The AUC values for the ROC curves in the testing sets approached 1 for the nomogram model (**Figure 4E**).

### 3.5 GGI networks, GSEA enrichment analysis, and ANN model

**Figure 5:**
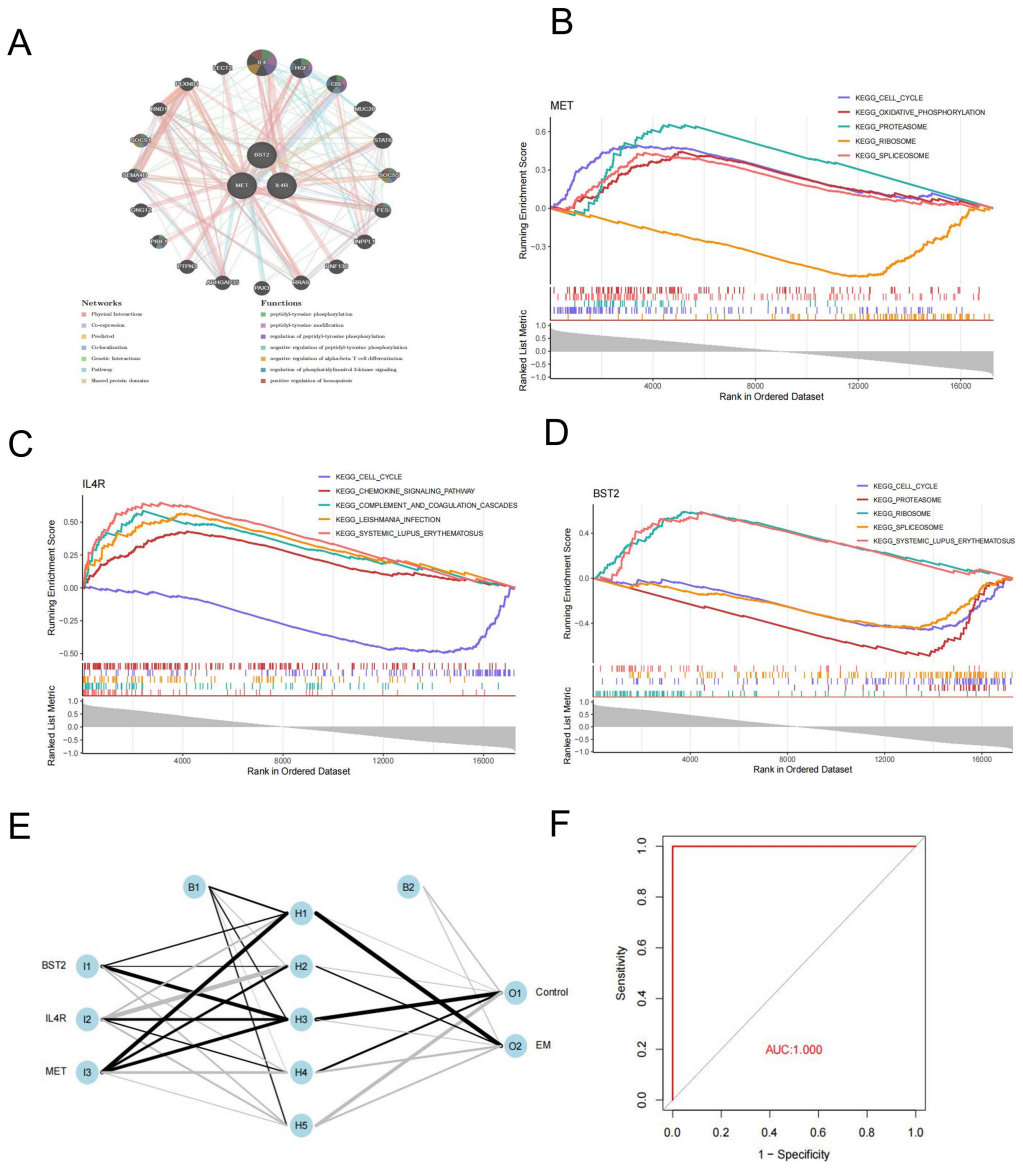
Gene-gene interaction networks and Enrichment pathway analysis of key genes. **(A)** GGI of key genes. **(B–D)** MET, IL4R and BTS2 GSEA analysis. **(E)** Construction of artificial neural network (ANN) and model evaluation. **(F)** ROC curves of the ANN model.

We used the GeneMANIA platform to establish a Gene-Gene Interaction Network (GGI). The analysis identified the top 20 correlated genes and the top 7 significant pathways related to the three key genes. The three key genes are linked to the pathways involving peptidyl-tyrosine phosphorylation, peptidyl-tyrosine modification, and regulation of peptidyl-tyrosine phosphorylation (**Figure 5A**).

We used GSEA analysis to study the specific signaling pathways involved in key genes in endometriosis. The findings revealed that three genes were significantly enriched in cell cycle pathways. Additionally, BST2 and MET were found to be enriched in the proteasome, ribosome, and spliceosome pathways (**Figures 5B–D**).

Three hub genes were integrated to construct an artificial neural network(ANN) in the training datasets (**Figure 5E**). A prediction model was then established based on the weights of the three key genes and the neural network. The ROC curves indicated that the key genes exhibited AUC values higher than 0.9 in the training set, demonstrating their significant potential in neural network prediction (**Figure 5F**).

### 3.6 Validation of the potential immunoregulatory properties of key genes

To investigate the differences in the immune microenvironment between the EM group and the control group, we employed immune infiltration analysis as well as an association analysis between key genes and immune checkpoints and factors.

#### 3.6.1 Immune infiltration analysis

**Figure 6:**
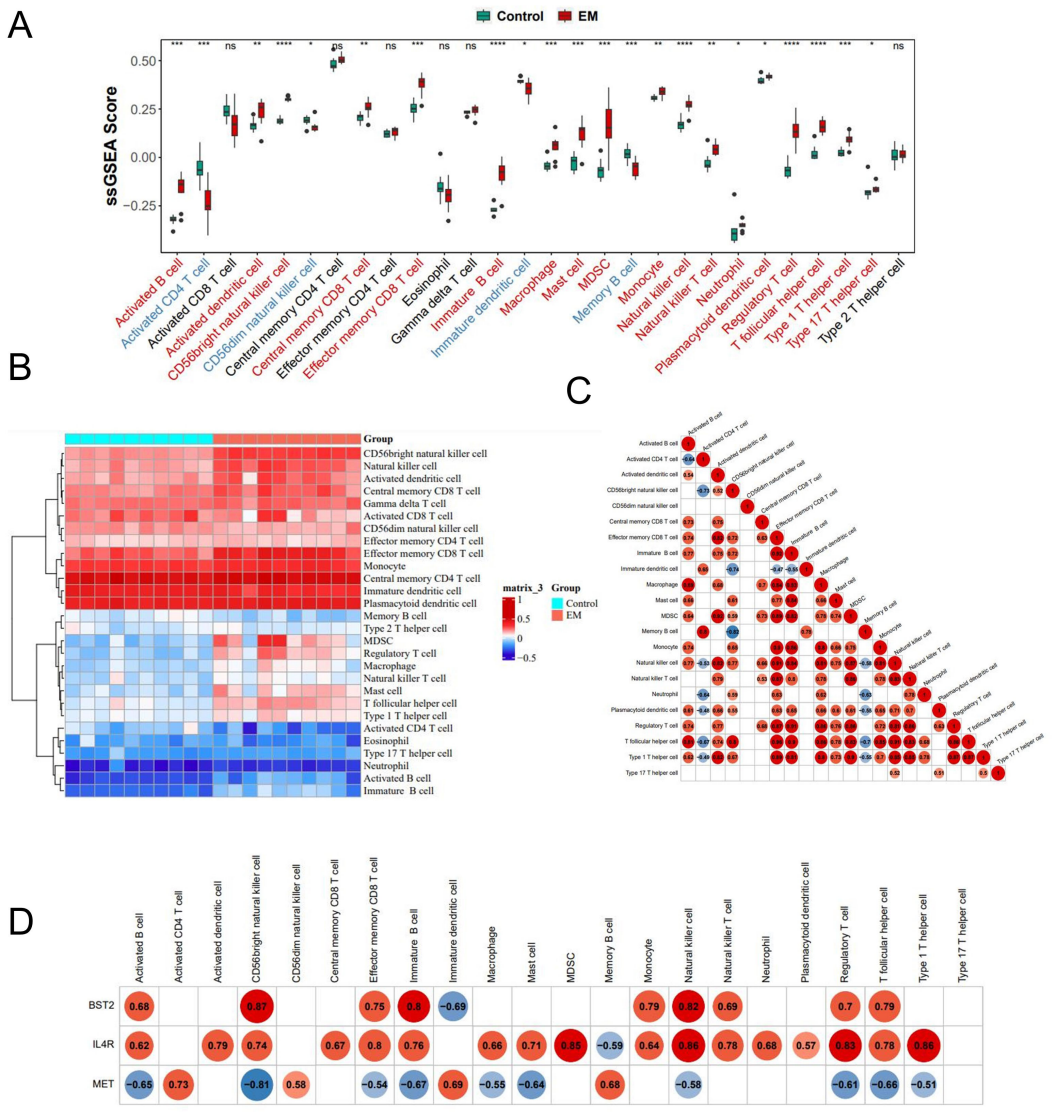
The profiles of immune cell subtype distribution pattern in training cohort. **(A)** The boxplots of the immune infiltration landscape in EM and control. **(B)** The heatmap of 22 immune differentially infiltrated fraction. **(C)** Correlation heatmap of all 22 immune cells. **(D)** Correlation heatmap of 3 key genes and 22 immune cells.

Significant differences were observed in twenty-two kinds of immune cells between the disease and control groups (p<0.05) (**Figures 6A, B**). The heatmap revealed that, among the 22 kinds of immune cell types, BST2 demonstrated the highest positive correlation with CD56bright natural killer cells (r = 0.87, P<0.05). Otherwise, CD56bright natural killer cells correlated with Memory B cells(r=-0.82, P<0.05) (**Figure 6C**). Conversely, MET exhibited the most significant negative correlation with this cell type (r = -0.81, P<0.05) (**Figure 6D**).

#### 3.6.2 Association between key genes and checkpoints

**Figure 7:**
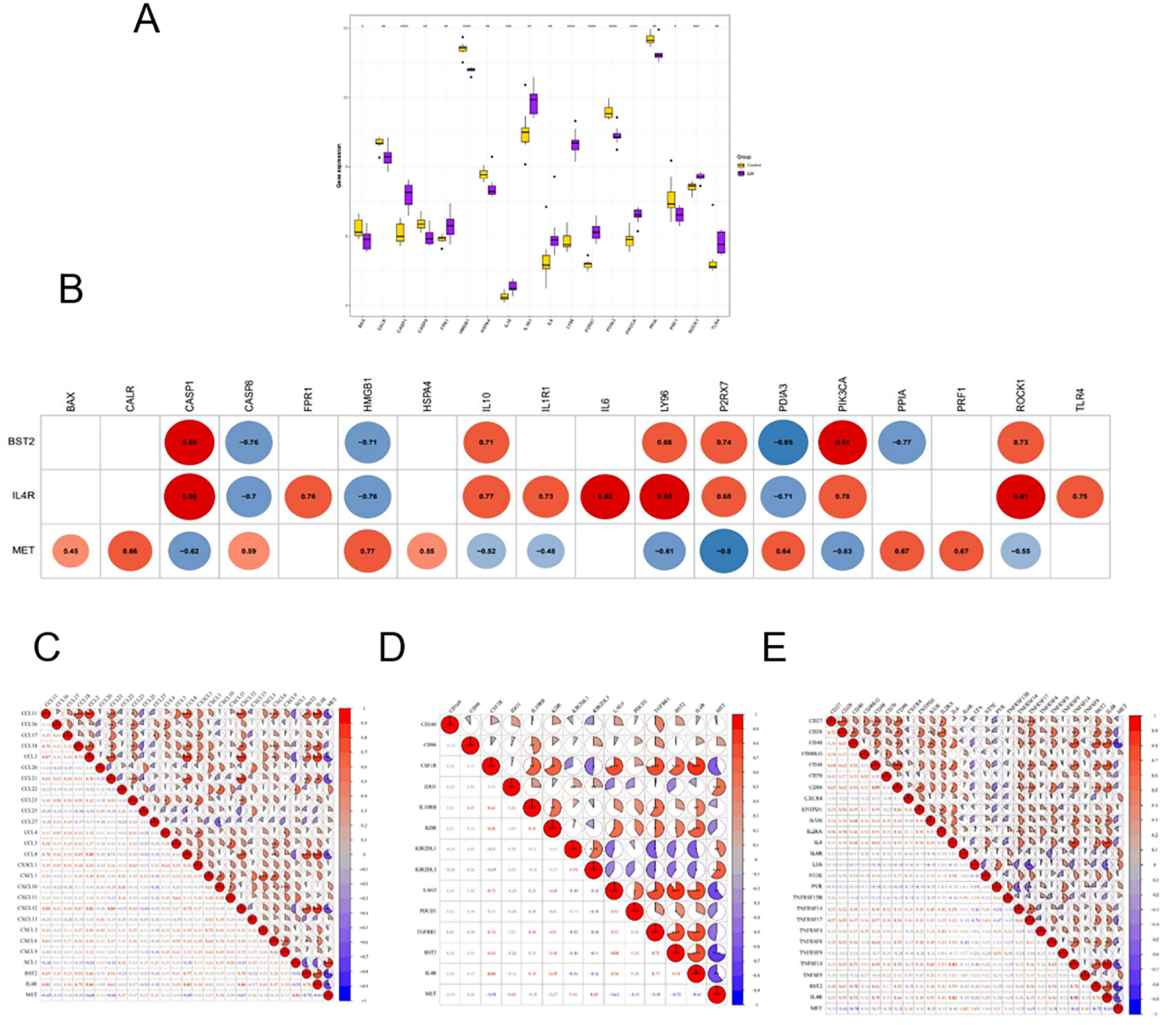
**(A)** Expression levels of immune checkpoint genes in EM and control group. **(B)** correlation between key genes and checkpoint. **(C–E)** Correlation between key and chemokine, immunosuppressive cytokine and immunostimulatory factor.

Differential comparisons of immune cell scores between the EM and control groups indicated significant differences at 18 immune checkpoints as illustrated by box plots using the ggplot2 package (**Figure 7A**). Additionally, the relationships between the key genes and the 18 differential immune checkpoints were examined (**Figure 7B**). The strongest positive correlation was found between BST2 and CASP1 (r = 0.85, P < 0.05), whereas the most significant negative correlation was identified between BST2 and PDIA3 (r = -0.85, P < 0.05). Furthermore, a significant positive correlation was observed between MET and HMGB1 (r = 0.77, P < 0.05), whereas a notable negative correlation was detected between MET and P2RX7 (r = -0.8, P < 0.05). The most robust positive correlation was observed between IL4R and CASP1 (r = 0.89, P < 0.05), whereas the most notable negative correlation was observed between IL4R and HMGB1 (r = -0.76, P < 0.05).

#### 3.6.3 Analysis of correlation between key genes with immune factors

The examination of the relationship between key genes and chemokines indicated that the most significant positive correlation existed between BST2 and CCL8 (r = 0.77, P<0.05), whereas the most substantial negative correlation was observed between BST2 and XCL1 (r = -0.56, P<0.05). The strongest positive correlation between MET and XCL1 was found (r = 0.83, P<0.05), whereas the most significant negative correlation was found between MET and CCL2 (r = -0.68, P<0.05). Conversely, the most significant positive correlation was observed between IL4R and CCL2 (r = 0.84, P < 0.05), whereas the most negative correlation was observed between IL4R and XCL1 (r = -0.50, P < 0.05) (**Figure 7C**).

The analysis of the relationships between the key genes and immunosuppressive factors revealed several significant correlations. The strongest positive correlations were found between: BST2 and LAG3 (r=0.90, P<0.05), MET and KIR2DL3 (r=0.61, P<0.05), and IL4R and CSFIR (r=0.84, P<0.05). The most significant negative correlations included: BST2 and KIR2DL1 (r = -0.46, P < 0.05), MET and LAG3 (r = -0.63, P < 0.05), and IL4R and KIR2DL1 (r = -0.54, P < 0.05) (**Figure 7D**).

The analysis of the relationship between key genes and immunostimulatory factors revealed a strong positive correlation between BST2 and CD40, TNFSF14 (r=0.70, P<0.05), MET and IL6R, LTA (r=0.45, P<0.05), and IL4R and TNFSF14 (r=0.93, P<0.05). On the other hand, the most significant negative correlations were between BST2 and LTA (r = -0.33, P < 0.05), MET and CD40 (r = -0.70, P < 0.05), and IL4R and LTA (r = -0.52, P < 0.05) (**Figure 7E**).

### 3.7 Validation of key genes in an online database and qRT-PCR

**Figure 8:**
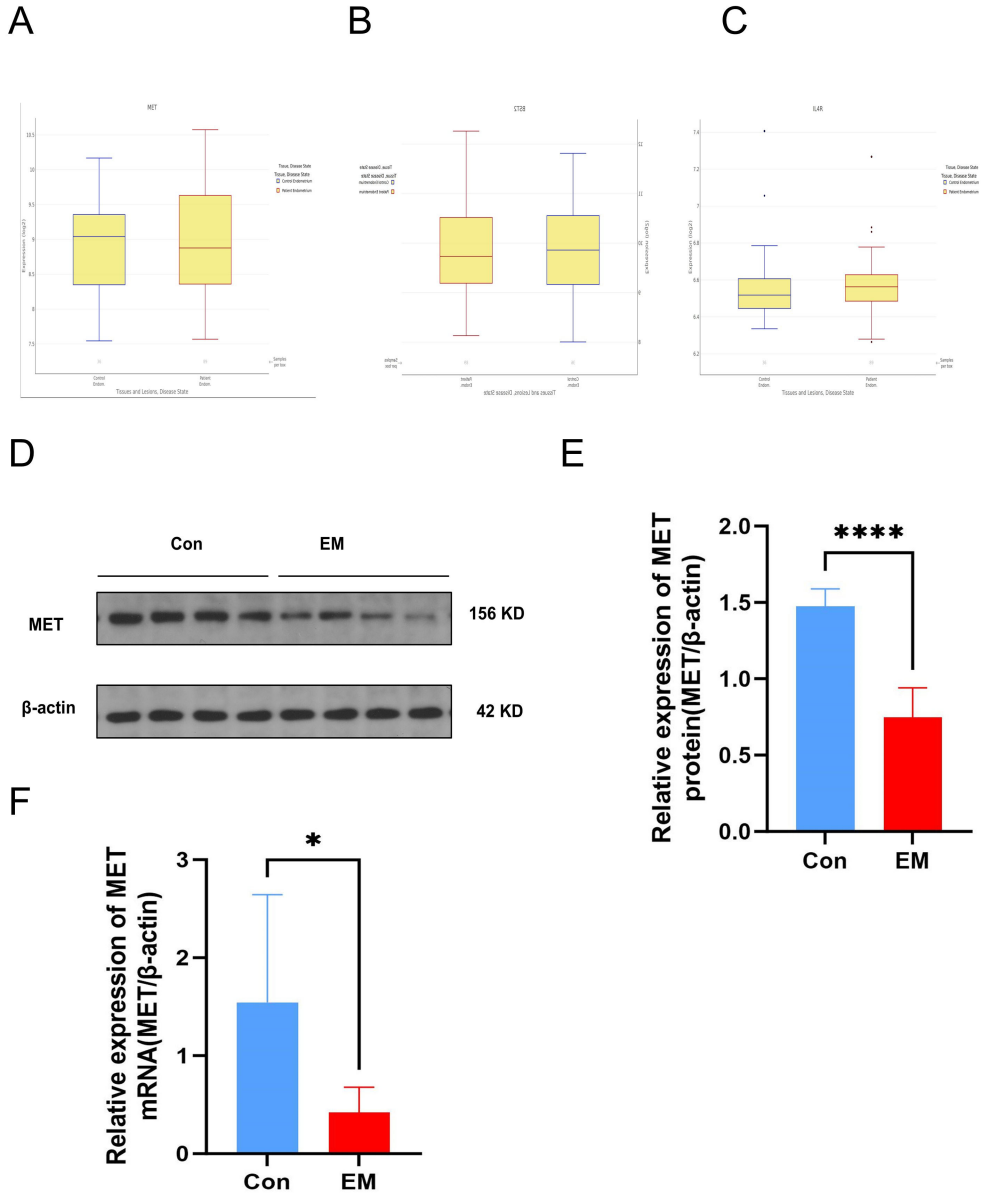
**(A–C)** Expression trend of three hub genes in an online dataset. **(D)** Protein expression MET detected by Western Blot in tissue sample. **(E)** Western Blot verified the difference of MET in protein in patients' tissue between EM and control. **(F)** RT-qPCR verified the difference in MET gene mRNA in patients' tissue between EM and control.

In addition to the GEO datasets, we identified the expression of the three hub genes in an online database, noting that the MET gene exhibited a consistent trend in both the GEO datasets and the online database (**Figure 8A**). In contrast, the gene BST2 exhibited an opposing trend, while IL4R exhibited a minor variation. Consequently, we assessed the expression of MET through qRT-PCR analysis and Western Blotting experiments using ectopic endometrial tissue from the EM group and normal endometrium from the control group (**Figure 8B**). Consistent with the bioinformatics analysis results, mRNA and protein expression of MET was significantly lower in EM tissues than in controls.

## 4 Discussion

Endometriosis is a chronic, inflammatory disease characterized by the growth of endometrial tissue outside of the uterine cavity. Immunological dysfunction has been proposed as a critical facilitator of ectopic lesion growth following retrograde menstruation of endometrial debris. One of the major challenges in contemporary gynecology is understanding and preventing the pathophysiology of endometriosis. An enhanced understanding of immune and inflammatory mechanisms occurring at the site of endometriotic lesion development may provide invaluable insight into the disease’s pathogenesis of the disease.

In this study, we analyzed bioinformatics databases to identify the potential key genes involved in endometriosis. Initially, we intersected 1,189 DEGs and 1,996 immune and 200 inflammation genes, screening out 13 DEGs correlated with inflammation and immunity. Subsequently, LASSO regression analysis, SVM-RFE analysis, and Boruta machine learning were performed to select five potential key genes from 13 DEGs. By comparing the differences in the expression of these five genes in both the training and external validation sets, we identified 3 key genes (MET, IL4R, and BST2) that exhibited consistent trends and significant differences between the training and validation sets. Finally, we validated MET expression in online datasets and endometriosis samples to confirm its role as a hub gene.

In the present study, based on IRGs, we identified a hub gene that is closely associated with intrinsic characteristics, such as molecular markers and therapeutic targets. Our findings confirm the close correlations between these three genes and immunoregulatory mechanisms, demonstrating both positive and negative relationships with immune cells, chemokines, and immune factors to varying degrees. Endometriosis, a disease influenced by immune-related mechanisms, has prompted numerous studies to identify biomarkers associated with immune function. Wang et al. utilized lasso regression and MCP-counter to identify immune-related biomarkers (13). In another study, He et al. intersected differentially expressed genes (DEGs) with transcription factors (TFs) from two databases and immune-related genes (IRGs) from the ImmPort database. Subsequently, they constructed a protein-protein interaction (PPI) network using Cytoscape to identify hub genes (14). In contrast, our study intersected DEGs with immune and inflammation-related genes, integrated machine learning results, validated key findings using training datasets and online databases, and investigated immunoregulatory properties. We aimed to screen and identify key genes using a comprehensive step-by-step screening process, enhancing the accuracy and consistency of our research.

The MET proto-oncogene, located on chromosome 7q21–q31 (15), encodes the tyrosine kinase receptor of the hepatocyte growth factor (HGF). It is characterized as a single-pass transmembrane receptor comprising an extracellular domain, transmembrane and juxtamembrane regions, and a tyrosine kinase domain (16). In cancer, aberrant MET signaling contributes to tumor invasion and regulates various physiological processes, including embryogenesis, wound healing, liver regeneration, angiogenesis, and immunomodulation (17–20). In a study by Finisguerre et al., Met was essential for neutrophil chemoattraction and cytotoxicity. MET is induced by inflammatory stimuli in both mouse and human neutrophils, subsequently activating the endothelium, inducing iNOA production upon HGF stimulation, and promoting cancer cell death (21). These findings were consistent with those of our analysis, suggesting that the immunological role of MET may be linked to the onset and progression of endometriosis.

IL4R is a specific cell surface receptor with which IL-4 interacts to exert its activities. The IL-4 receptor (IL-4R) signaling system consists of two receptors: type 1 IL-4R and type 2 IL-4R (22, 23). IL-4 and IL-13 share a common receptor subunit and signaling pathway involving the Janus kinase (JAK): STAT6 pathway (24). IL-4 and IL-13 play important roles in Th2 immune responses, metabolism, tissue regeneration, remodeling, cancer, learning, and memory (25). Bone marrow stromal cell antigen 2 (BST2) is expressed in numerous cells, including hepatocytes, plasma blast cells, early plasma cells, mature B cells, dendritic cells, pneumocytes, monocytes, pancreatic cells, kidney cells, and vascular endothelial cells, suggesting that it plays vital roles in the innate immune response against viral infection and other physiological processes (26, 27). Increased BST2 expression has been observed in multiple human cancers, including hematological tumors and solid tumors, especially in breast cancer, HCC, gastrointestinal cancer, and lung cancer (28) via activating signaling pathways, such as EGFR/AKT, NF-κB/ERK, and GRB2/DIM/caspase 3 (29–31). All three genes are involved in signatures related to immunomodulation, but their roles in endometriosis remain unreported, and further studies are needed to elucidate their function in endometriosis.

We validated the three hub genes using the Turku Endometriosis Database and tissue samples collected from women with and without non-endometriosis. The expression of the MET gene in both the online database and clinical tissue showed the same downregulated trend in the EM group as in the training and two validation sets. MET is overexpressed in a variety of tumors, whereas we observed low expression levels of MET in the EM group. MET may play a distinct role in immune regulation in endometriosis. The composition of the immune microenvironment in endometriotic lesions is associated with different disease stages, phenotypes, and symptoms of the disease (32). The functional properties of MET in EM may vary depending on the stages and phenotype of the endometriosis. Endometriotic lesions develop in complex and dynamic environments. MET was the target gene of our study, and further studies are required to understand its role in the pathogenesis of endometriosis.

In endometriosis, the network of immune cell populations originating from both the innate and adaptive immune systems creates an optimal environment for lesion formation in the ectopic endometrium. Numerous studies have attempted to construct immune-related gene signatures as diagnostic markers and correlate these genes with patterns of immune cell infiltration. Macrophages play a crucial role in the development of endometrial lesions and contribute to ectopic cell survival (33). A previous study found that the number of macrophages increased in the eutopic endometrium of patients with endometriosis (34). Additionally, macrophages polarize from M2 (anti-inflammatory phenotype) to M1 (proinflammatory phenotype) in the eutopic endometrium of patients with endometriosis compared to the control group (35). Ding et al. (36) and Cui et al. (37), focused on screening M2 macrophage-related genes based on the theory that immune cell dysfunction plays an important role in endometriosis using different analysis methods. In this study, we investigated the correlation between signature genes and infiltrating immune cells. It has been shown that CD56bright natural killer cells, related to two hub genes (MET and BST2), may exhibit dysfunction in EM. NK cells are key components of the innate immune system, serving as the initial defense against viral infections and tumor growth while playing a critical role in maintaining normal tissue homeostasis (38). NK cell populations exist in both the peripheral circulation and the uterus and are primarily characterized as CD56dimCD16+ and CD56brightCD16-, respectively (39). One study indicated that NK cells had less cytotoxicity in women with endometriosis than in those without it (40). Decreased cytotoxic can impair the clearance of endometrial fragments by NK cells in the peritoneal cavity, facilitating the implantation of ectopic implants. Julia et al. found that NK cytotoxic activity in the endometrium of women with endometriosis was lower than in normal endometrium. However, in cases where the endometriosis patient was infertile and/or experienced recurrent miscarriage, NK cytotoxic activity was elevated (41). Altered immune cells in the peritoneal cavity of women with endometriosis can influence other endometrial cell populations, thereby increasing their susceptibility to lesion formation, survival, and growth. Further studies are required to clarify the functional properties and interaction between MET and NK cells in endometriosis, which may provide in-depth insights into the pathophysiology of endometriosis.

The present study has several limitations. First, we validated the downregulation of MET in endometriosis(EM). However, the regulatory mechanisms of MET in EM need to be investigated, particularly the correlation between MET and NK cells. This can be achieved through both *in vivo* and *in vitro* experiments. Second, there was a lack of clinical information regarding the patient’s fertility and disease severity. Therefore, the mechanism by which this gene affects the progression of endometriosis throughout the menstrual cycle needs to be confirmed by experiments based on large sample numbers.

## Data Availability

The original contributions presented in the study are included in the article/**Supplementary Material**. Further inquiries can be directed to the corresponding author.
