# Supplementary material for: Identification and validation of immune-related and inflammation-related genes in endometriosis
Source: Front Endocrinol (Lausanne). 2025 May 8;16:1545670. doi: 10.3389/fendo.2025.1545670 (PMC12095003; doi:10.3389/fendo.2025.1545670)
Supplement: Supplementary file 4 [file DataSheet1.zip › Raw data/03Venn/fig02_VennDiagram_2.pdf]

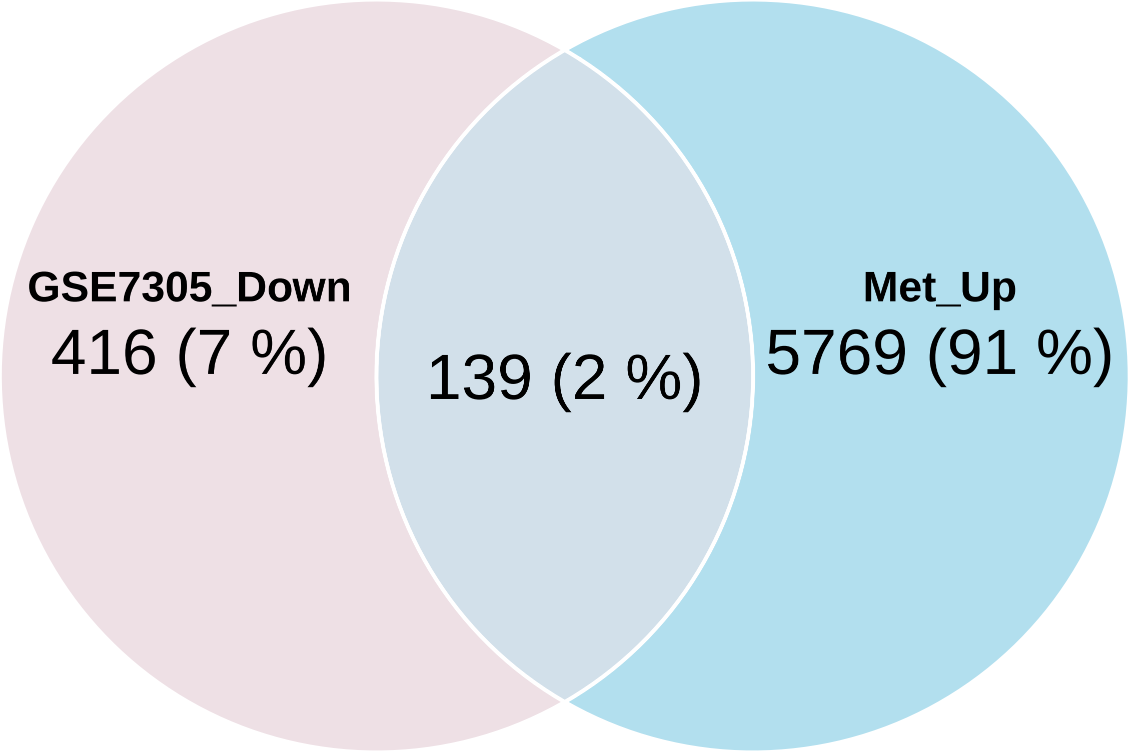
GSE7305_Down
416 (7 %)
139 (2 %)
Met_Up
5769 (91 %)
