# Supplementary material for: Identification and validation of immune-related and inflammation-related genes in endometriosis
Source: Front Endocrinol (Lausanne). 2025 May 8;16:1545670. doi: 10.3389/fendo.2025.1545670 (PMC12095003; doi:10.3389/fendo.2025.1545670)
Supplement: Supplementary file 4 [file DataSheet1.zip › Raw data/06machine/fig04_LASSO&SVMRFE_venn.pdf]

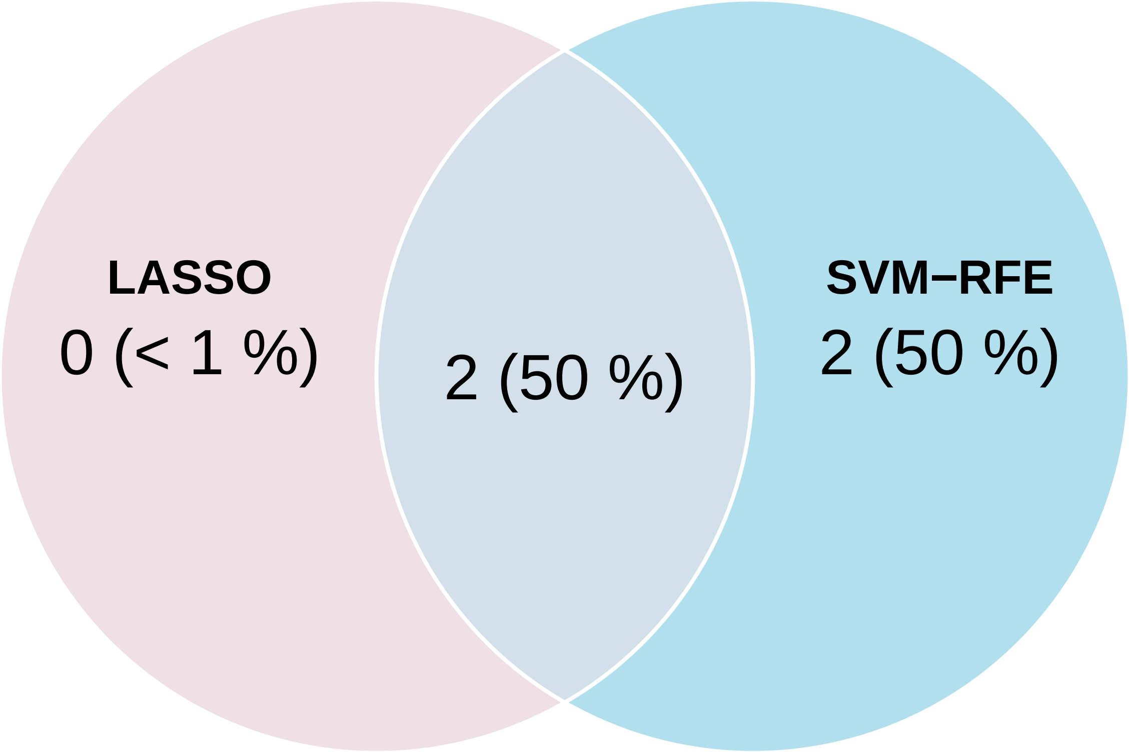

LASSO
0 (< 1 %)
2 (50 %)
SVM−RFE
2 (50 %)
