# Supplementary figures and images for: Identification and validation of immune-related and inflammation-related genes in endometriosis
Source: Front Endocrinol (Lausanne). 2025 May 8;16:1545670. doi: 10.3389/fendo.2025.1545670 (PMC12095003; doi:10.3389/fendo.2025.1545670)

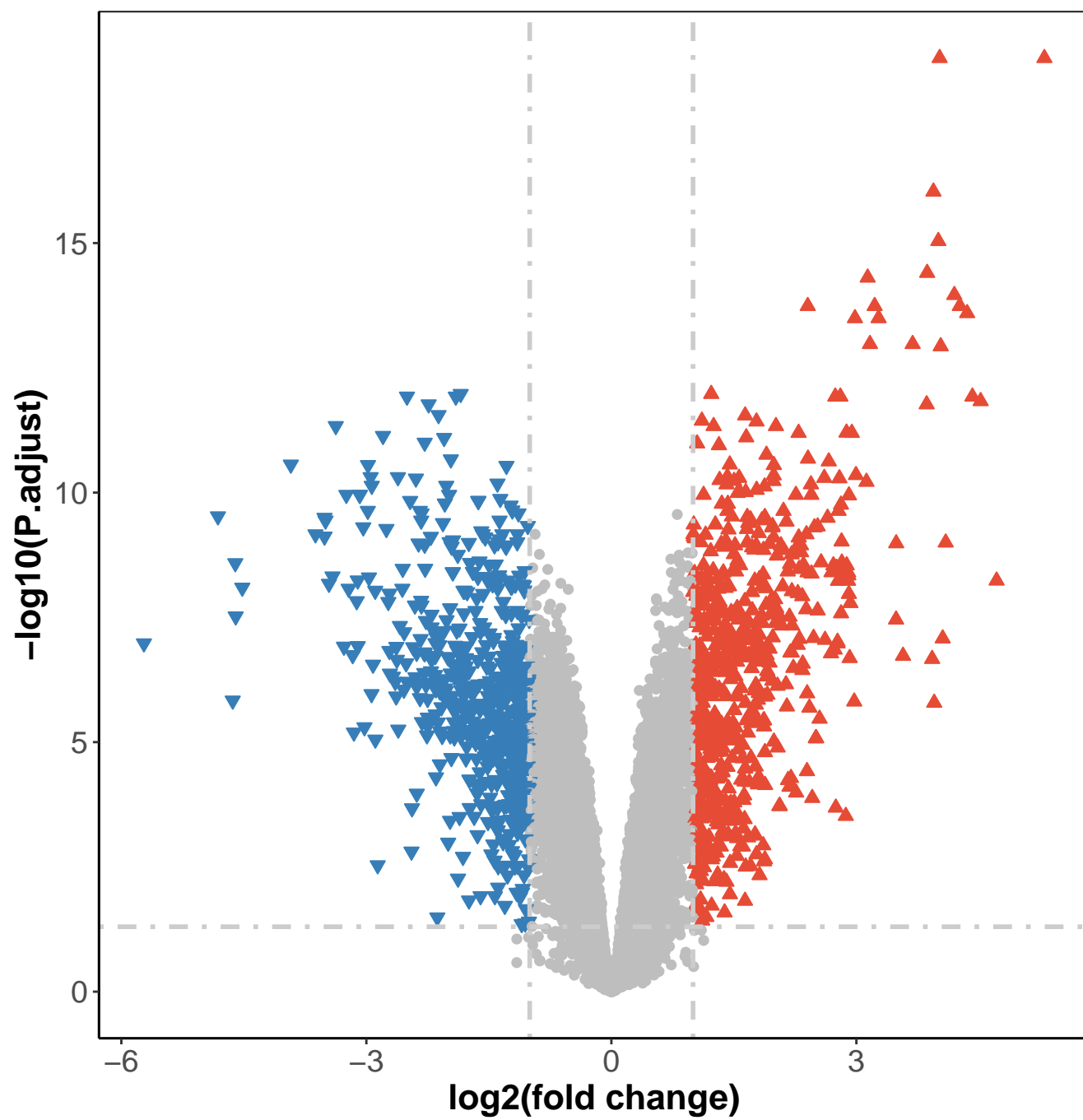

▲ Up expression ▼ Down expression • Non significant

Supplement: Supplementary file 4 [file DataSheet1.zip › Raw data/01DEGs/fig01_DEGs_volcano.pdf]

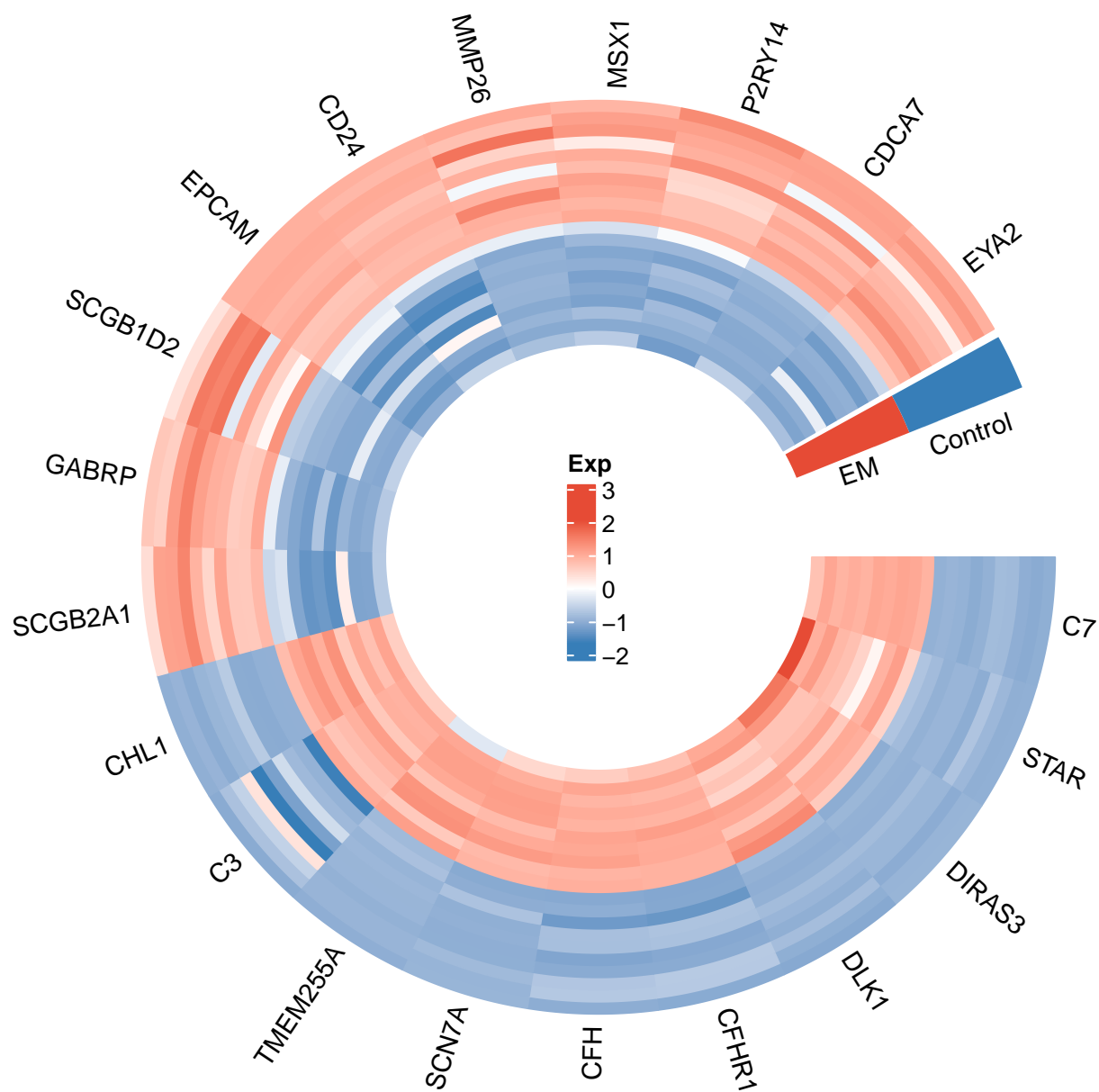

Supplement: Supplementary file 4 [file DataSheet1.zip › Raw data/01DEGs/fig02_DEGs_circpheatmap.pdf]

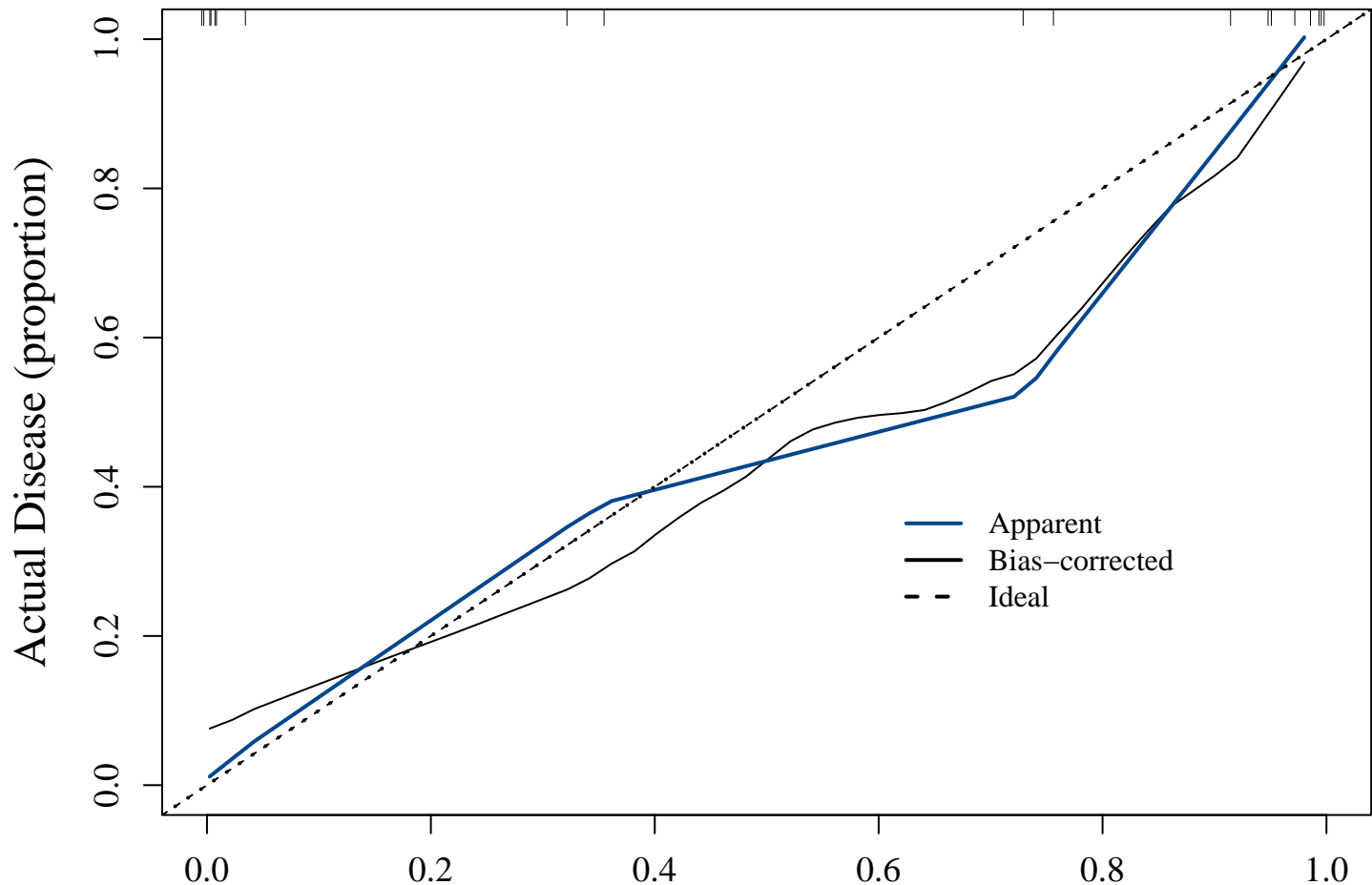

Nomogram–Predicted Probability of Disease risk

B= 30 repetitions, boot

Mean absolute error=0.075 n=19

Supplement: Supplementary file 4 [file DataSheet1.zip › Raw data/01_GSE23339/02.calibrate.pdf]

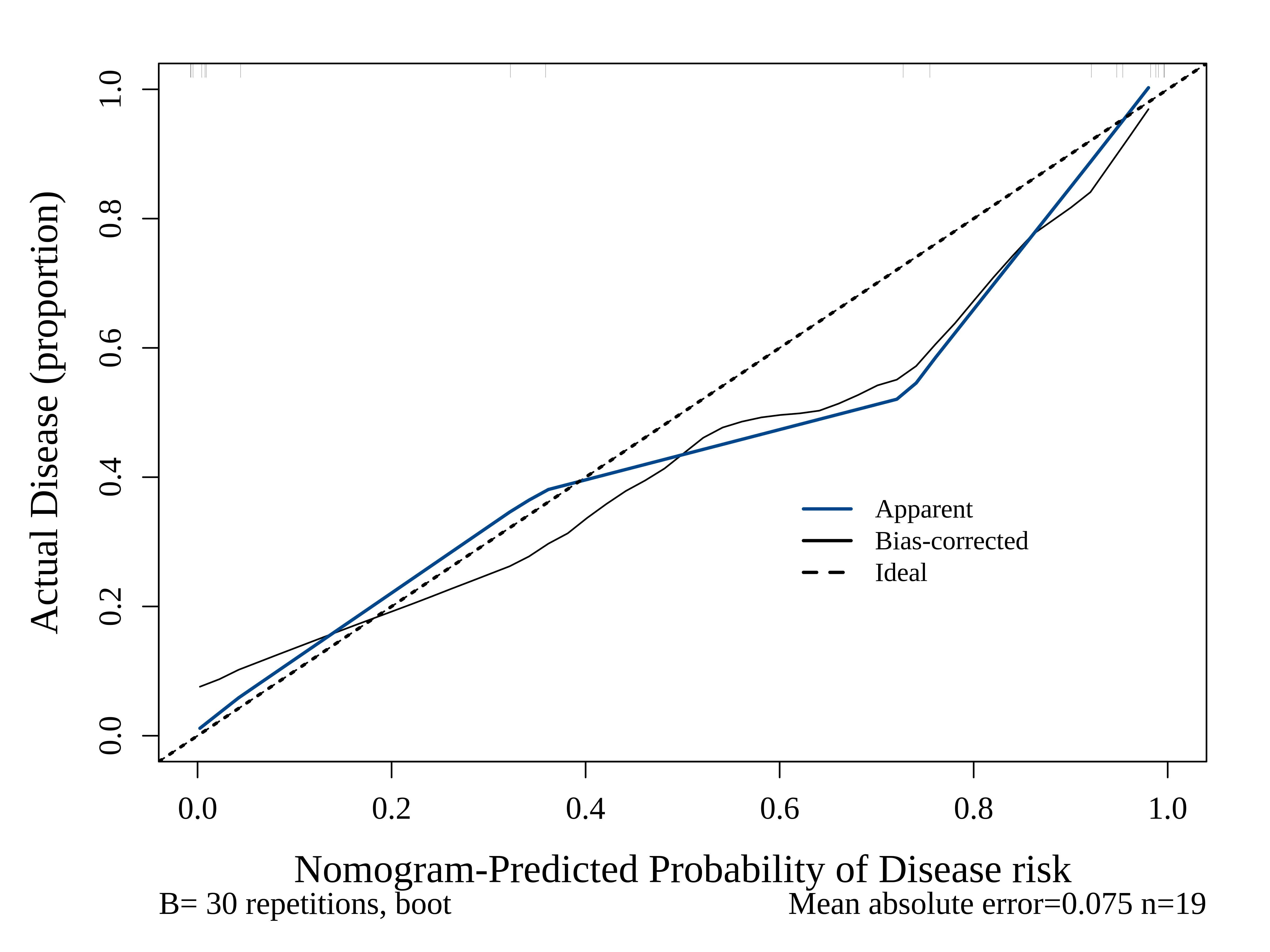

Supplement: Supplementary file 4 [file DataSheet1.zip › Raw data/01_GSE23339/02.calibrate.png]

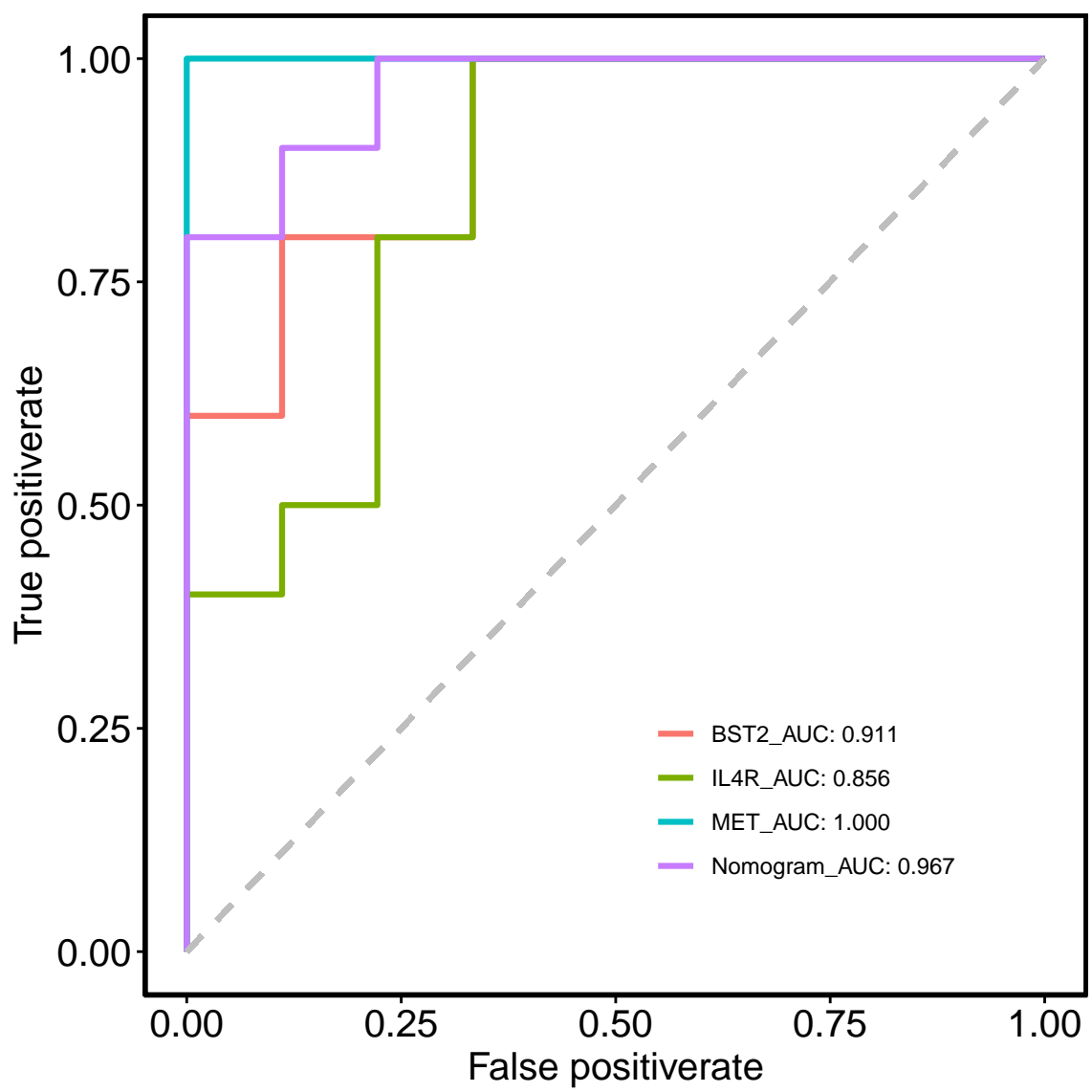

Supplement: Supplementary file 4 [file DataSheet1.zip › Raw data/01_GSE23339/03.ROC.pdf]

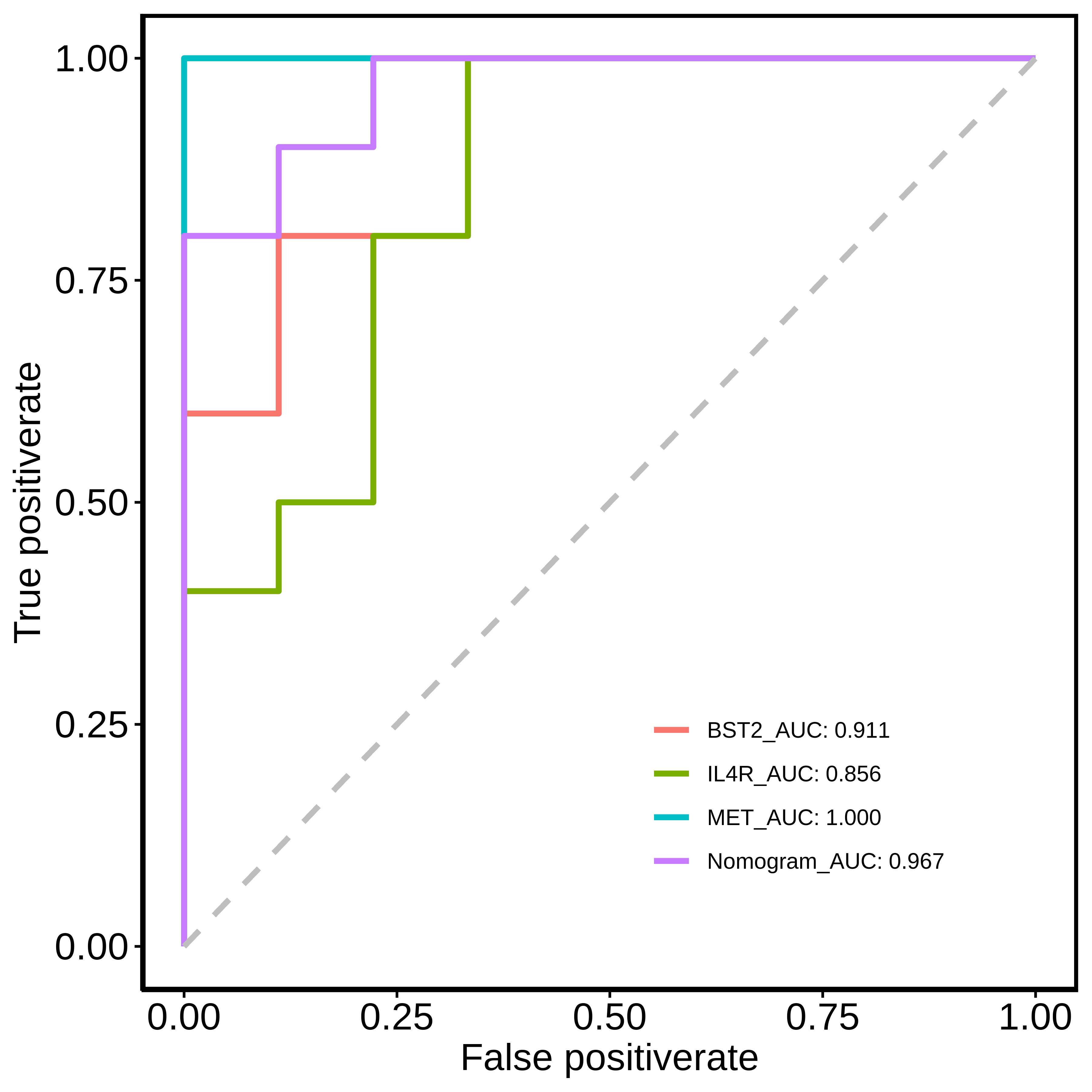

Supplement: Supplementary file 4 [file DataSheet1.zip › Raw data/01_GSE23339/03.ROC.png]

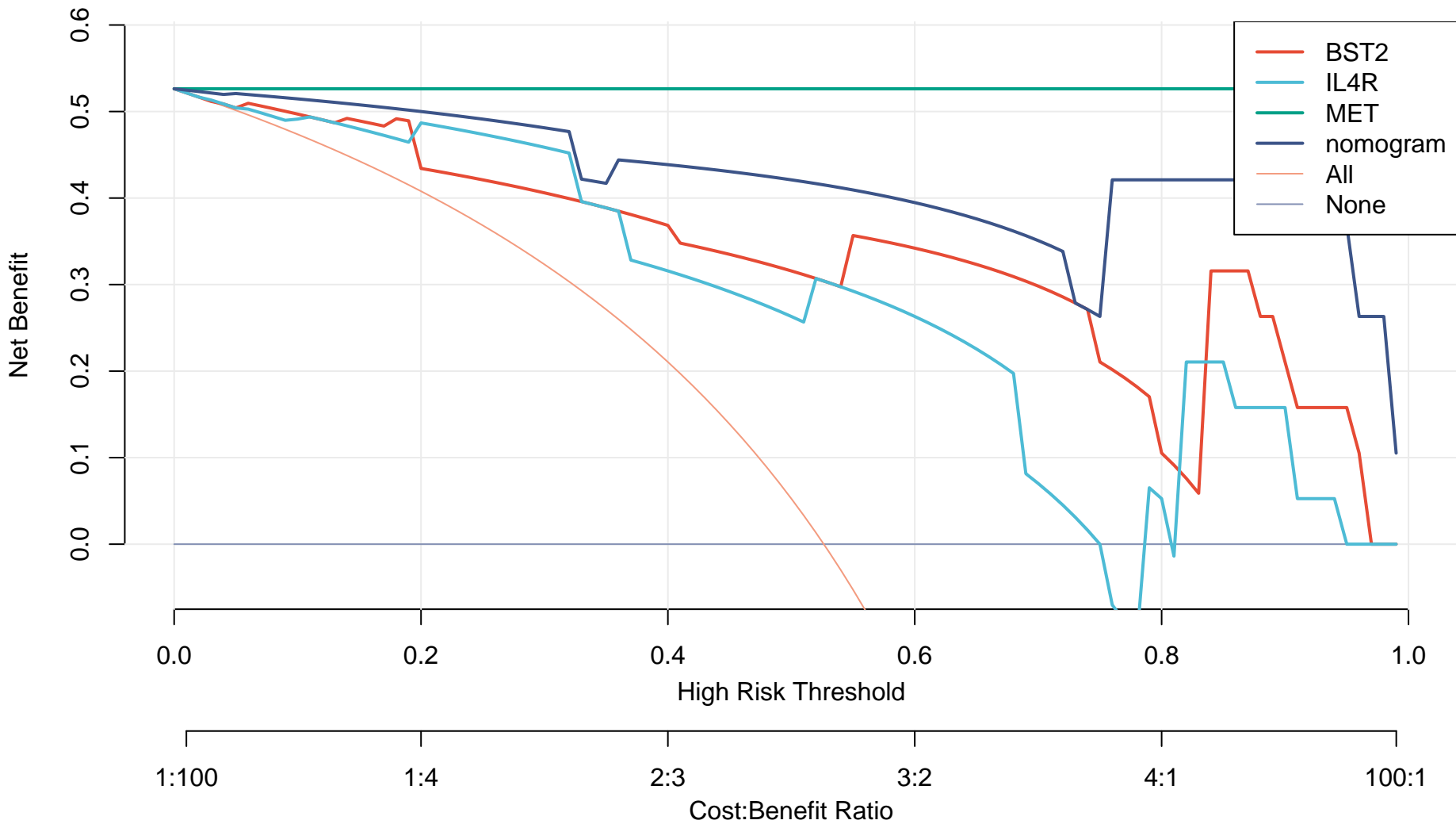

Supplement: Supplementary file 4 [file DataSheet1.zip › Raw data/01_GSE23339/04.DCA.pdf]

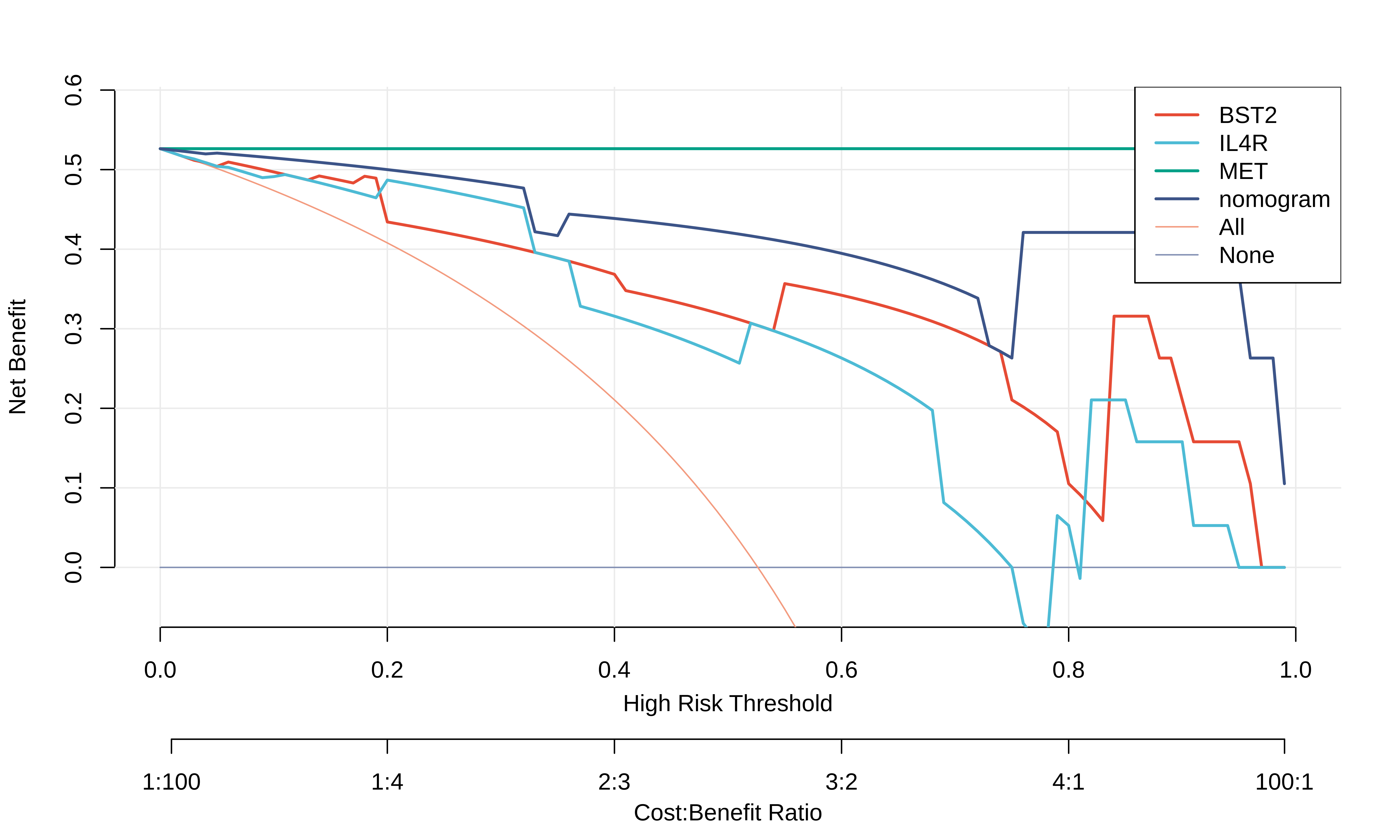

Supplement: Supplementary file 4 [file DataSheet1.zip › Raw data/01_GSE23339/04.DCA.png]

# All samples before normalization (403707 probes)

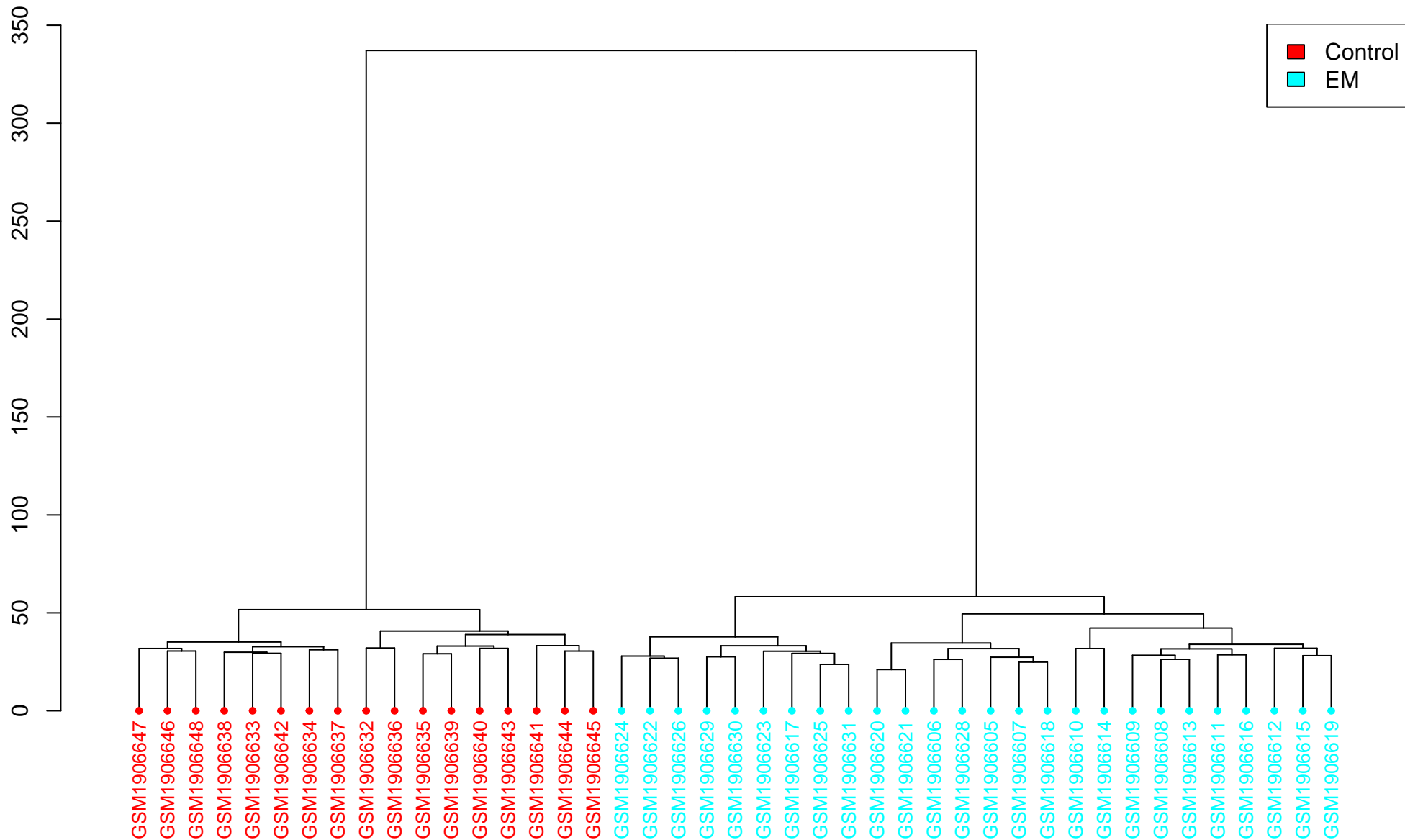

Supplement: Supplementary file 4 [file DataSheet1.zip › Raw data/02Met/fig01_SampleCluster.pdf]

**Density plot of raw data (403707 probes)**

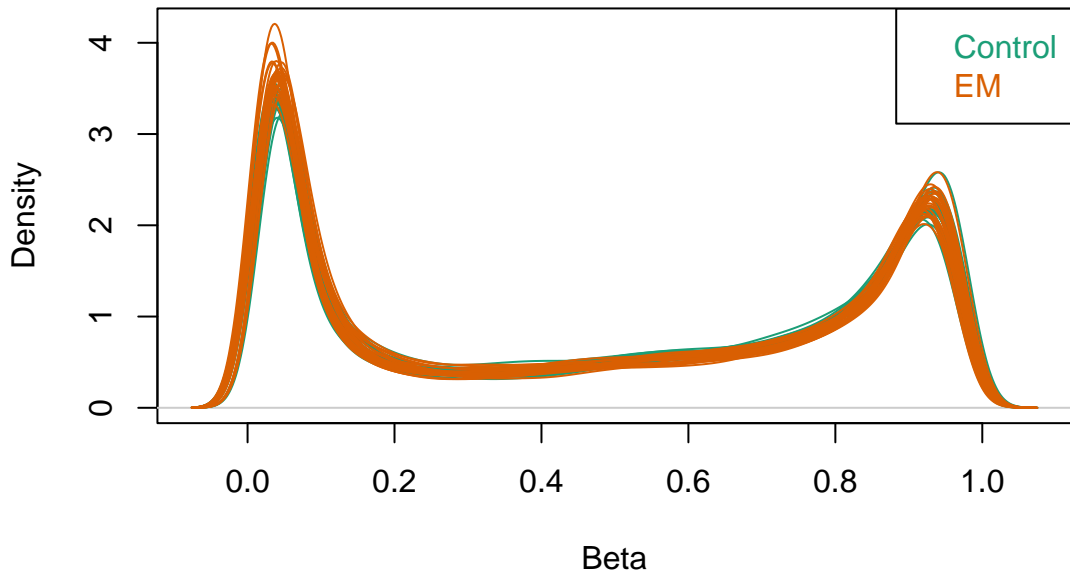

Supplement: Supplementary file 4 [file DataSheet1.zip › Raw data/02Met/fig03_densityPlot.pdf]

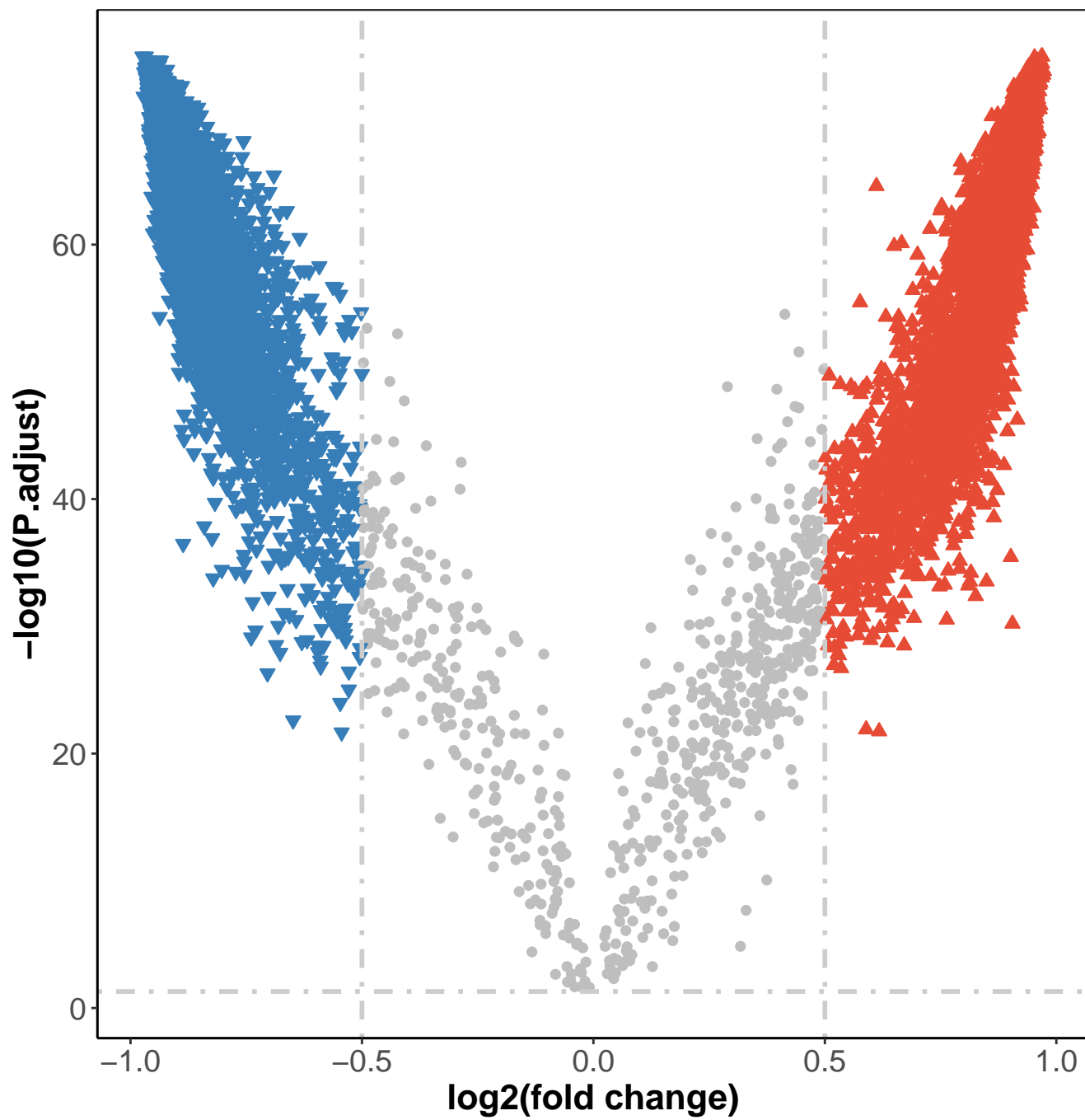

▲ Up expression ▼ Down expression • Non significant

Supplement: Supplementary file 4 [file DataSheet1.zip › Raw data/02Met/fig04_DEGs_volcano.pdf]

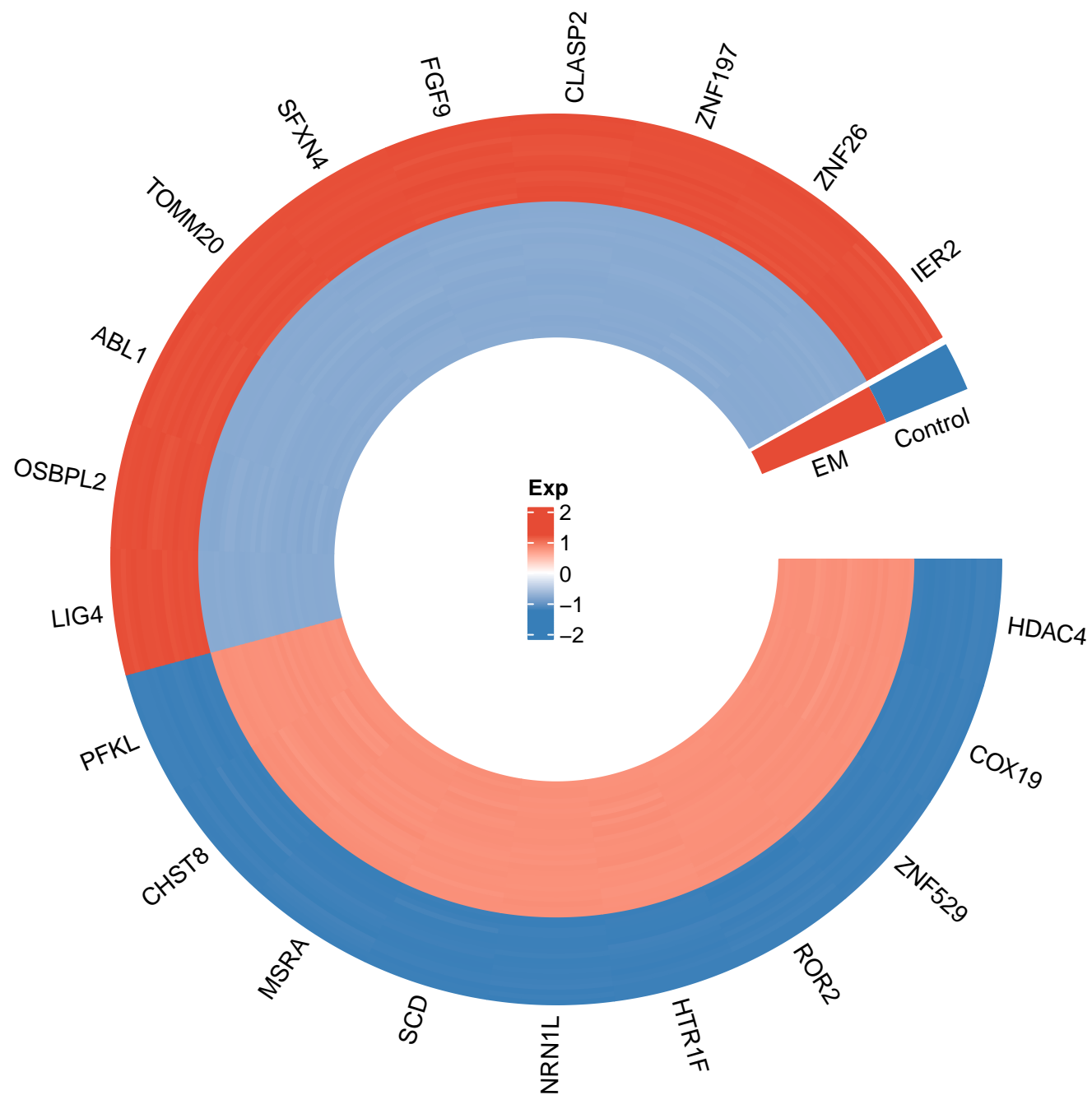

Supplement: Supplementary file 4 [file DataSheet1.zip › Raw data/02Met/fig05_DEGs_circpheatmap.pdf]

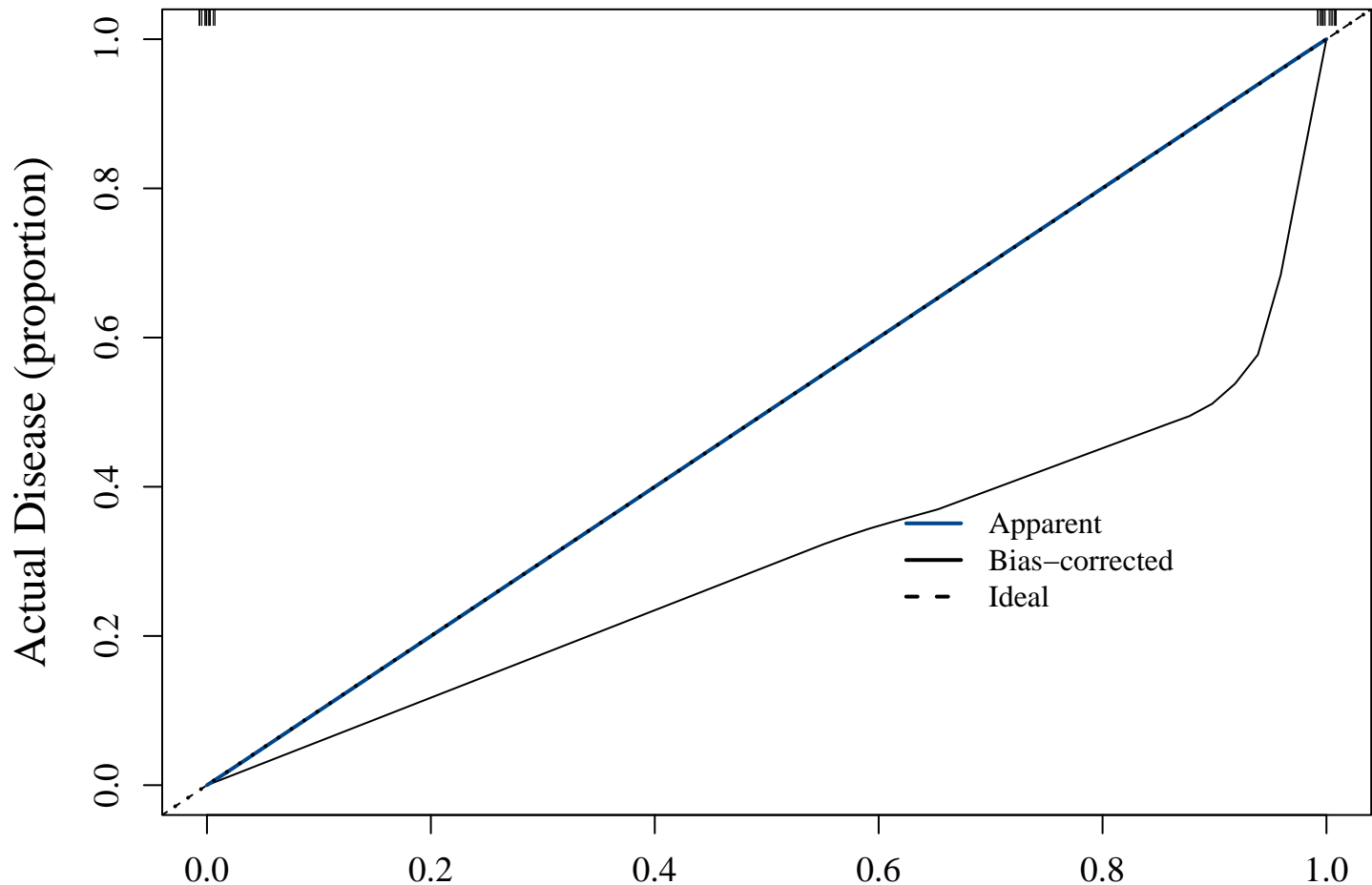

Nomogram-Predicted Probability of Disease risk

B= 30 repetitions, boot

Mean absolute error=0 n=36

Supplement: Supplementary file 4 [file DataSheet1.zip › Raw data/02_GSE7307/02.calibrate.pdf]

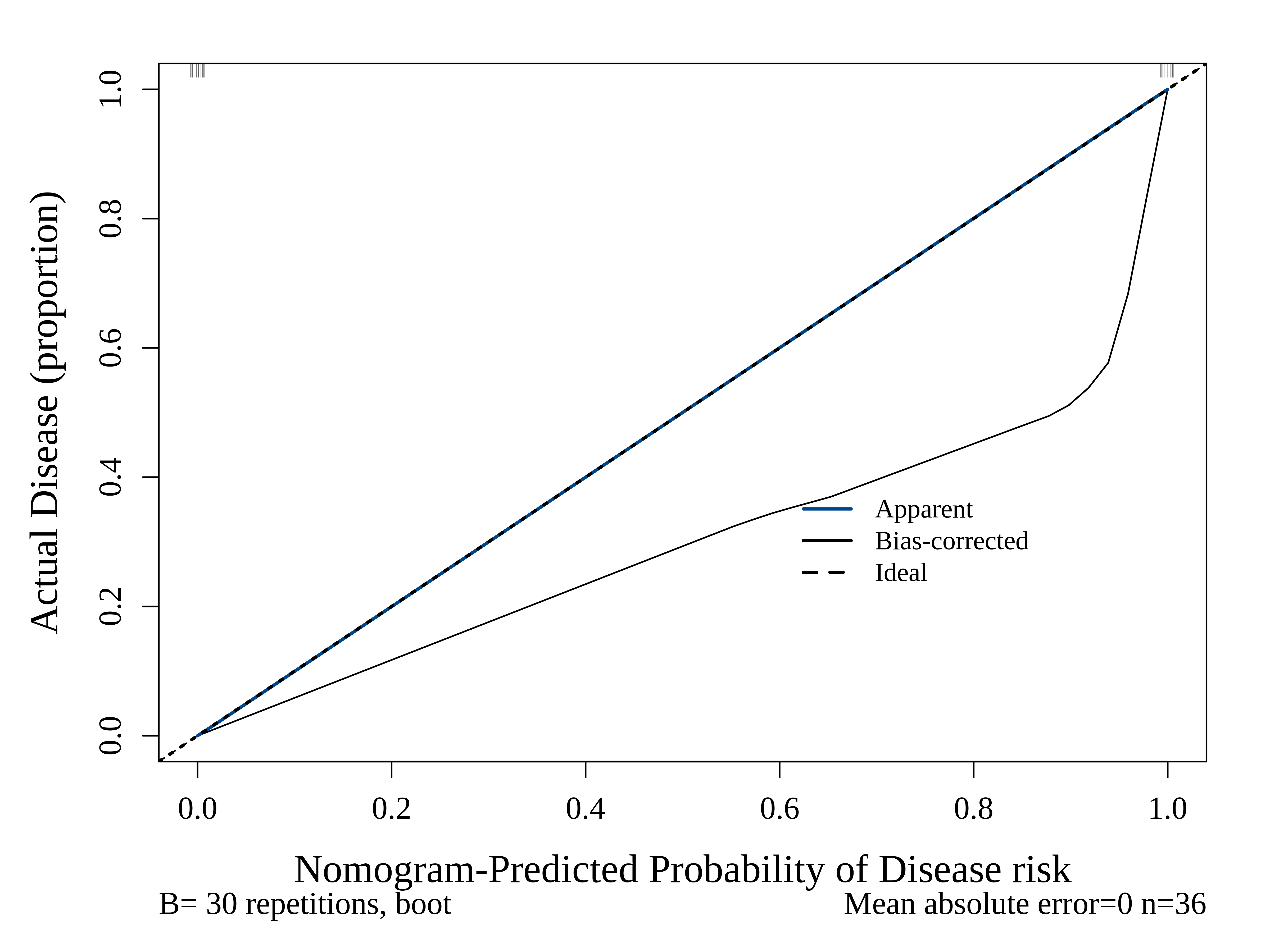

Supplement: Supplementary file 4 [file DataSheet1.zip › Raw data/02_GSE7307/02.calibrate.png]

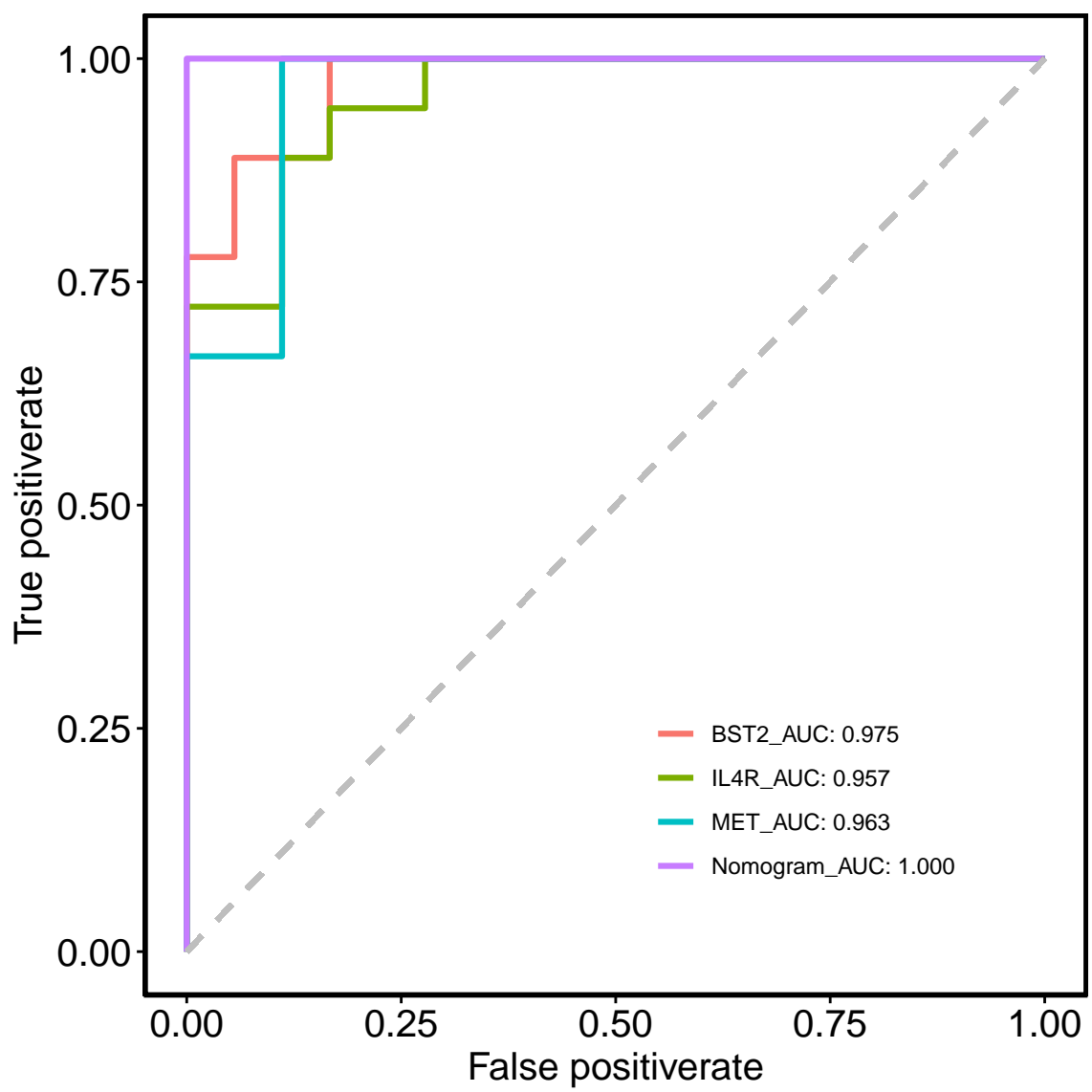

Supplement: Supplementary file 4 [file DataSheet1.zip › Raw data/02_GSE7307/03.ROC.pdf]

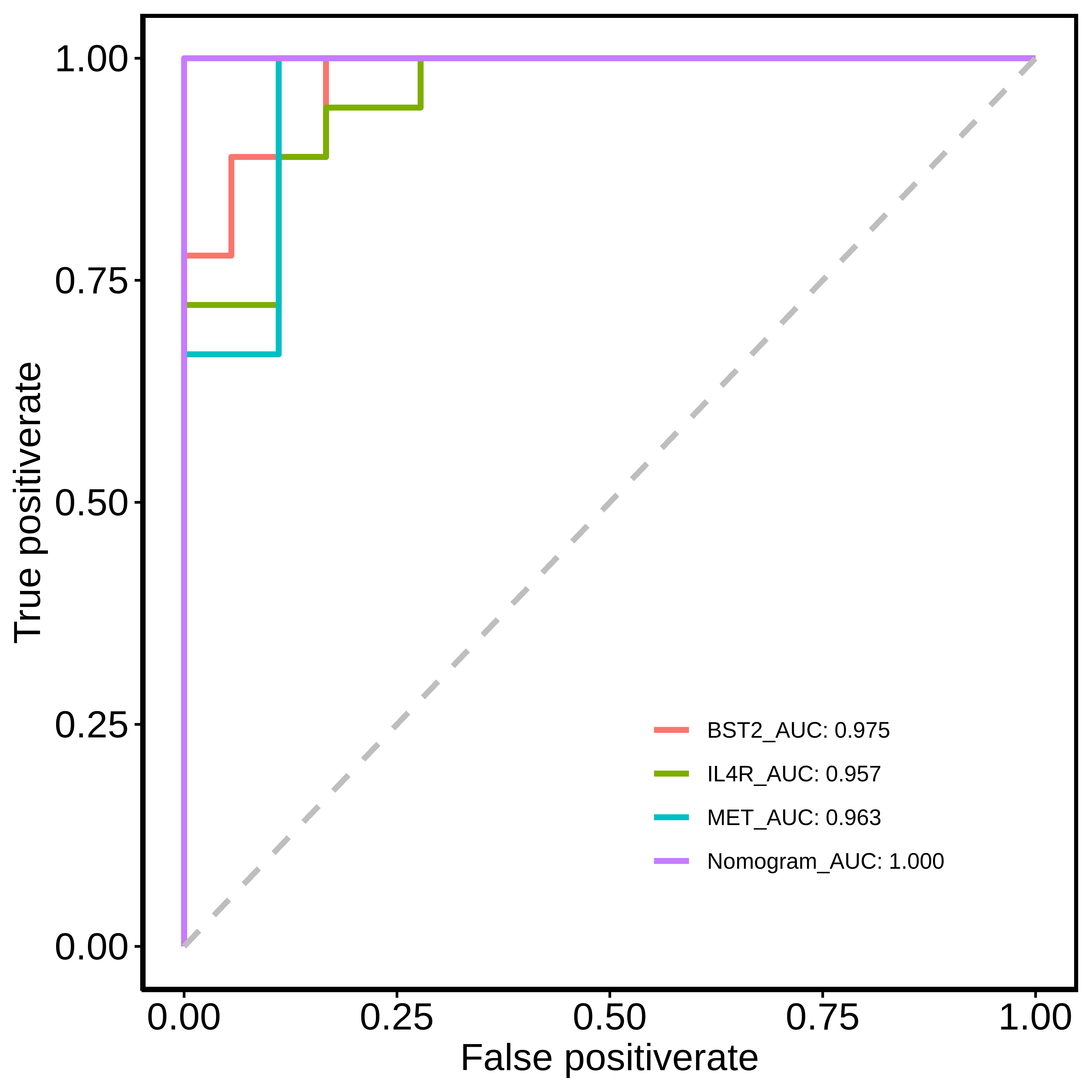

Supplement: Supplementary file 4 [file DataSheet1.zip › Raw data/02_GSE7307/03.ROC.png]

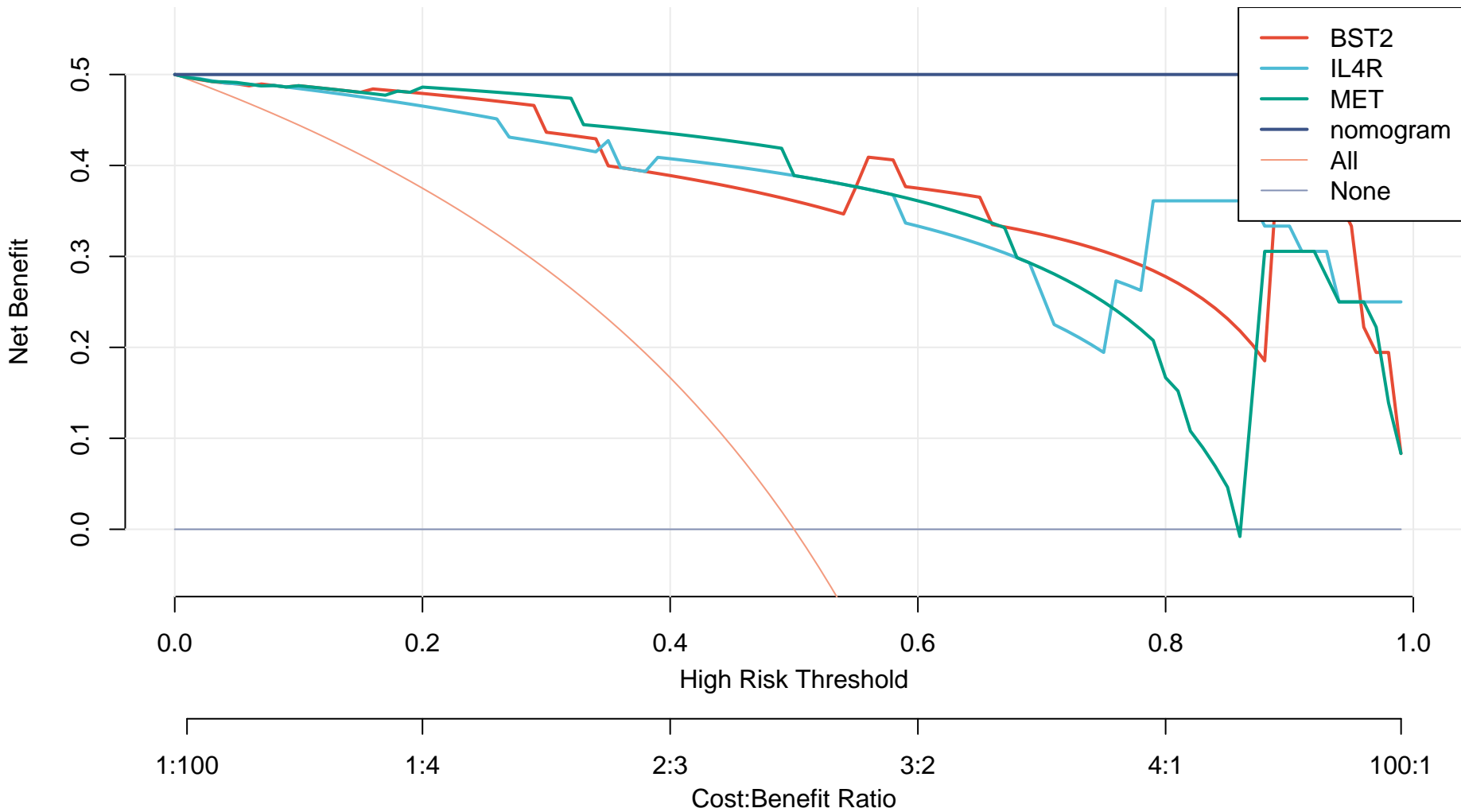

Supplement: Supplementary file 4 [file DataSheet1.zip › Raw data/02_GSE7307/04.DCA.pdf]

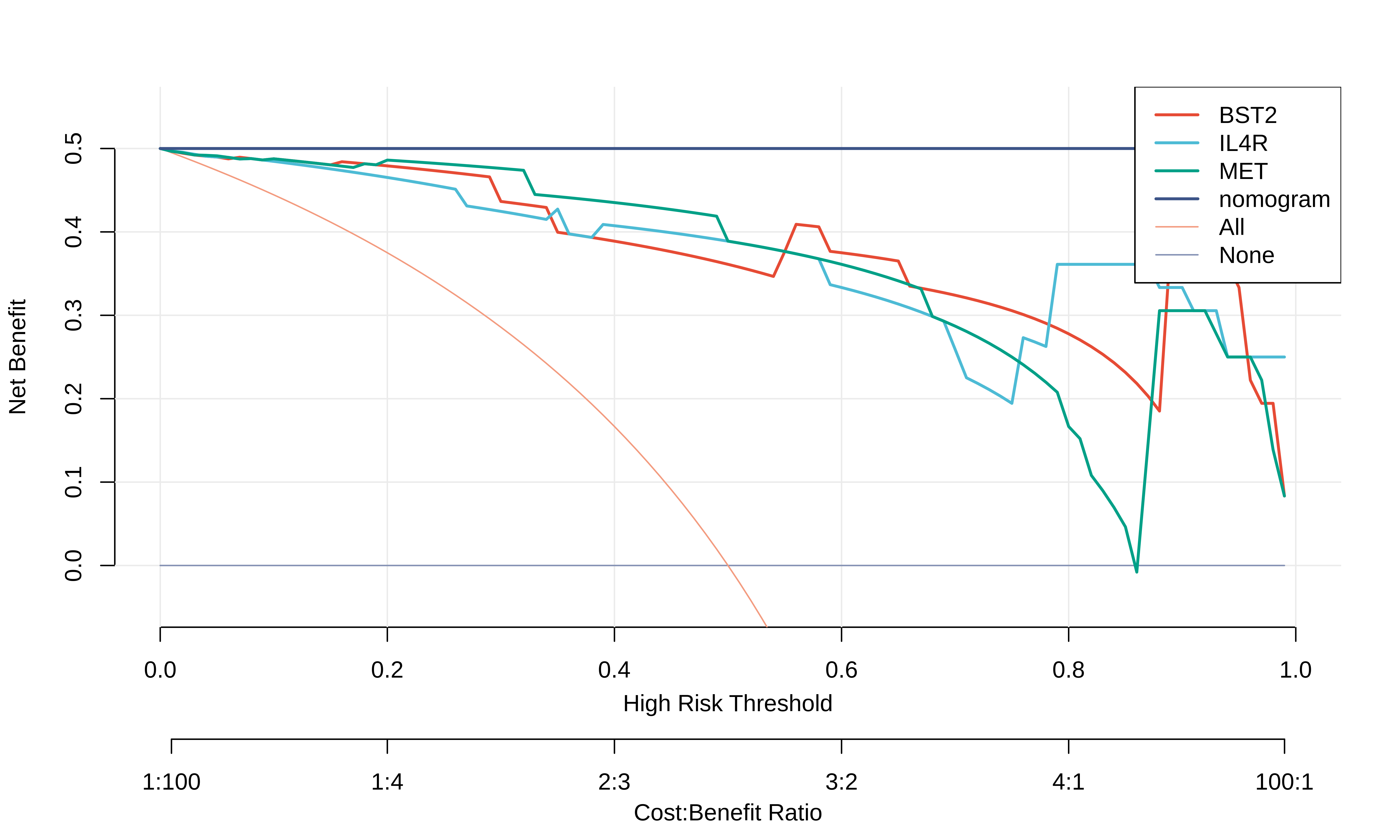

Supplement: Supplementary file 4 [file DataSheet1.zip › Raw data/02_GSE7307/04.DCA.png]

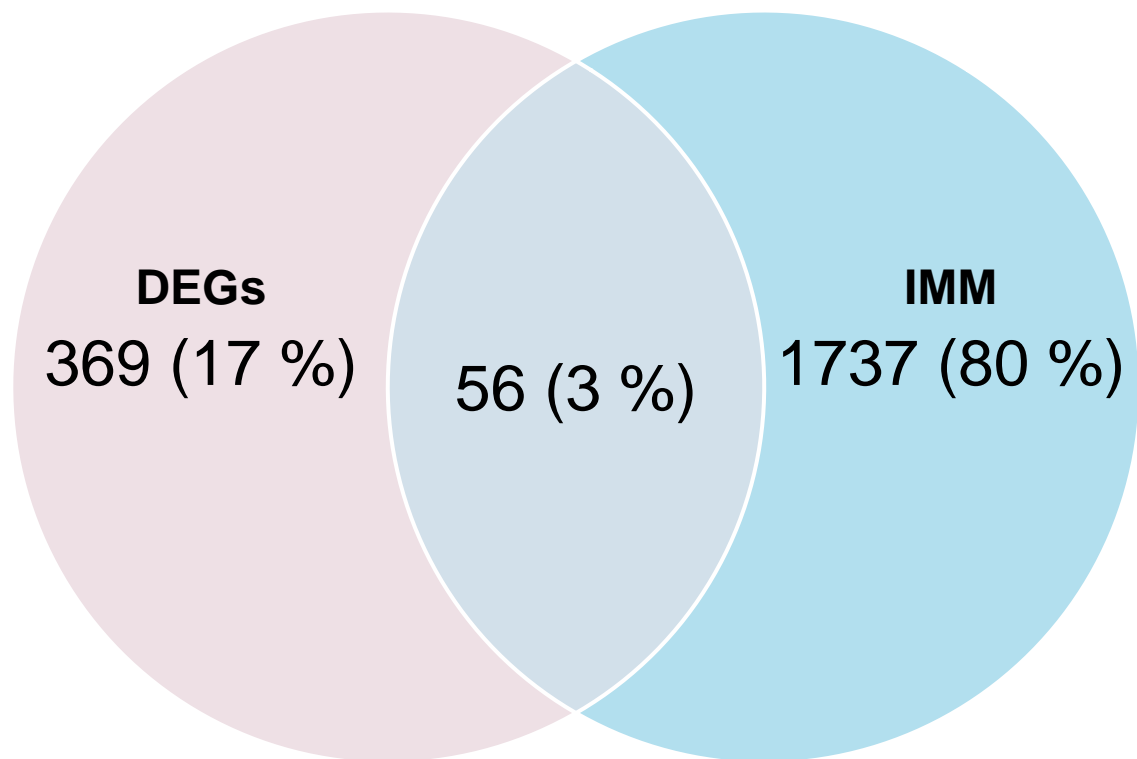

Supplement: Supplementary file 4 [file DataSheet1.zip › Raw data/03Venn/fig03_Venn_DEGs_IMM.pdf]

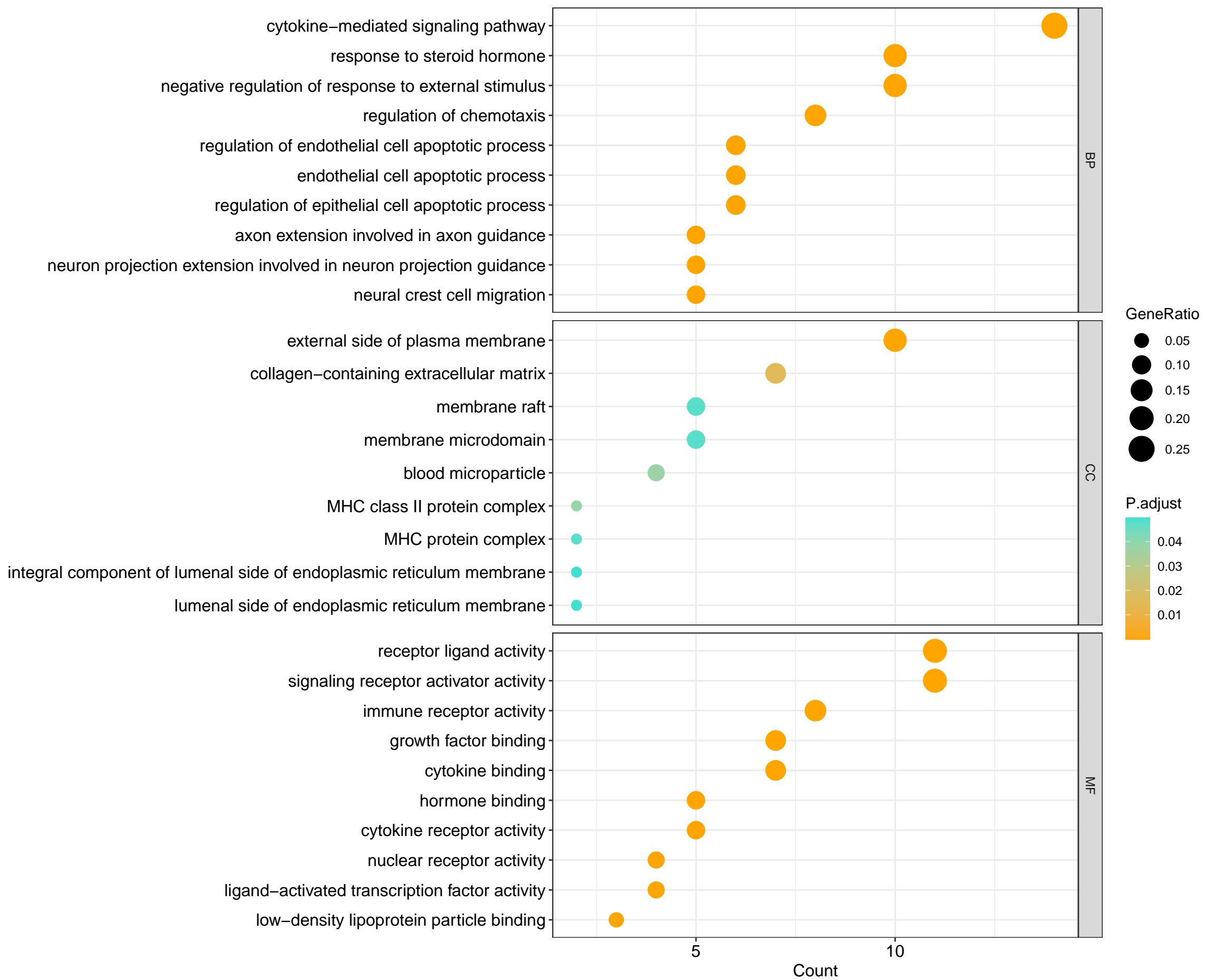

Supplement: Supplementary file 4 [file DataSheet1.zip › Raw data/04GO&KEGG/fig01_GO_dot_plot.pdf]

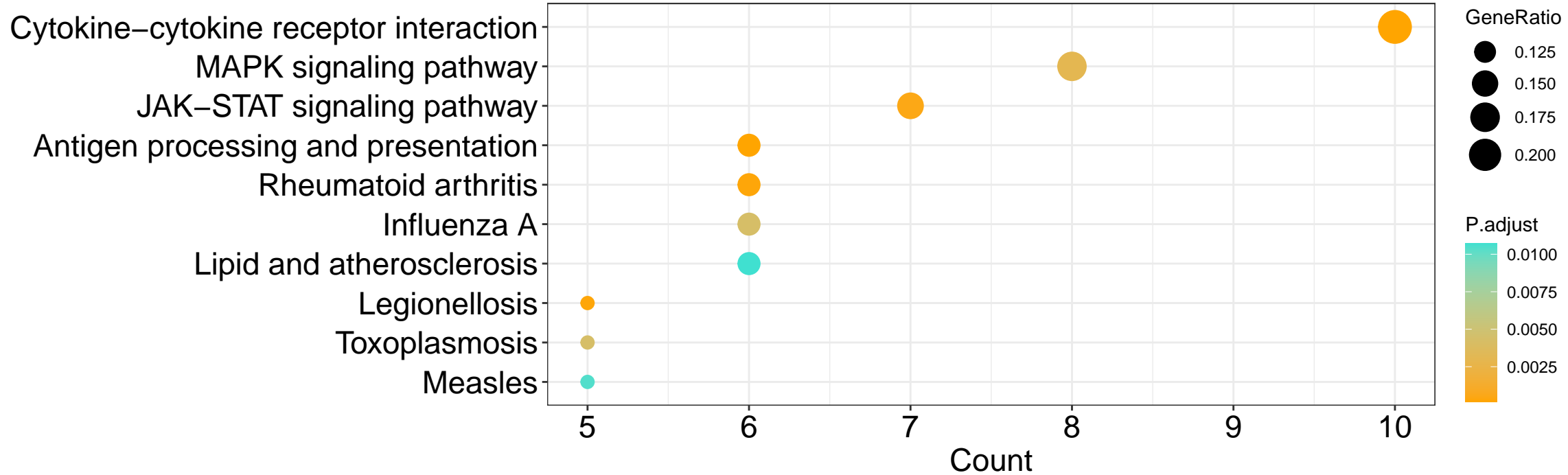

Supplement: Supplementary file 4 [file DataSheet1.zip › Raw data/04GO&KEGG/fig02_KEGG_dot_plot.pdf]

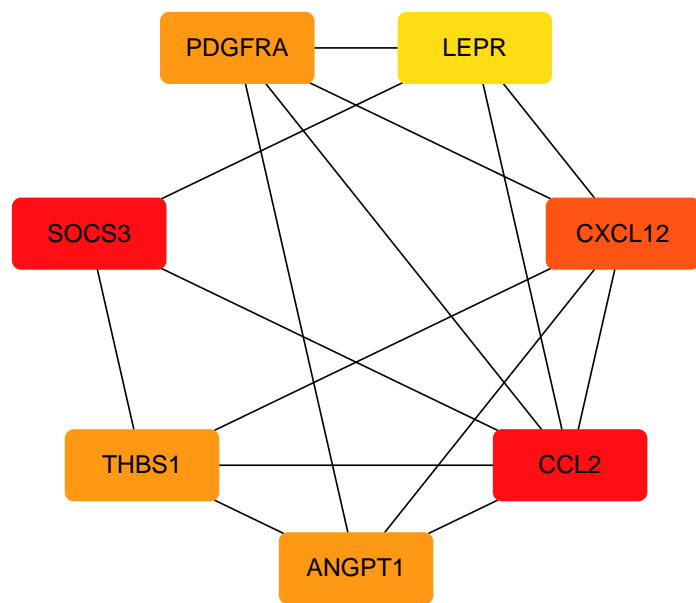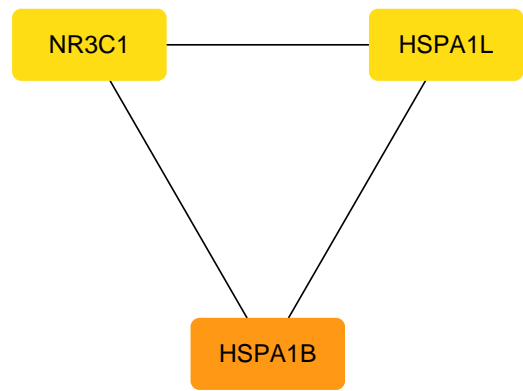

Supplement: Supplementary file 4 [file DataSheet1.zip › Raw data/05PPI/fig02_Degree_top10.pdf]

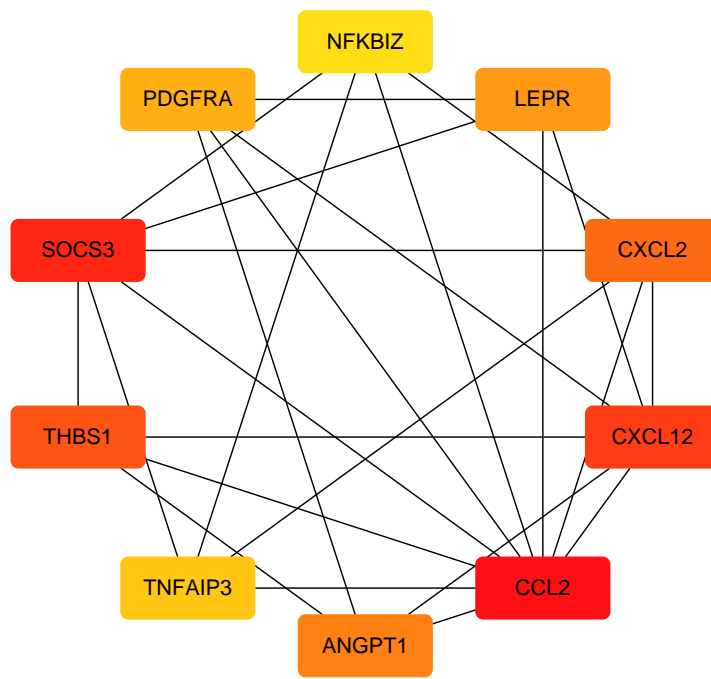

Supplement: Supplementary file 4 [file DataSheet1.zip › Raw data/05PPI/fig03_EPC_top10.pdf]

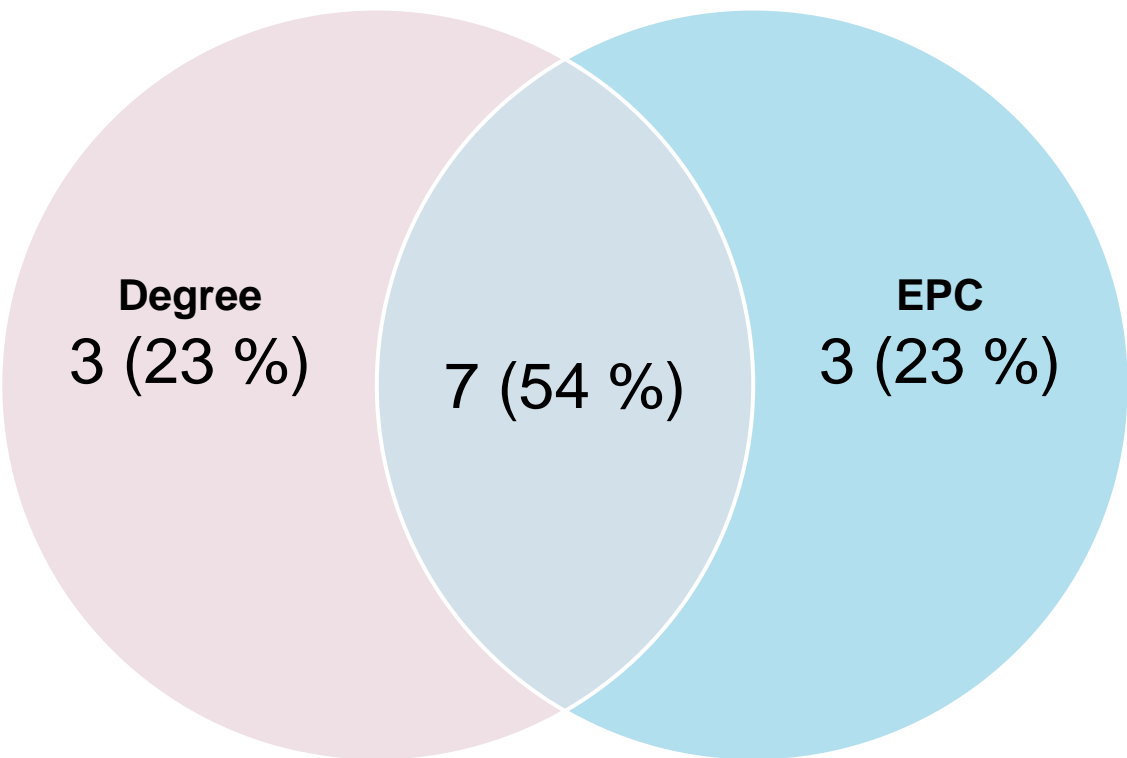

Supplement: Supplementary file 4 [file DataSheet1.zip › Raw data/05PPI/fig04_Venn_Degree_EPC.pdf]

Binomial Deviance

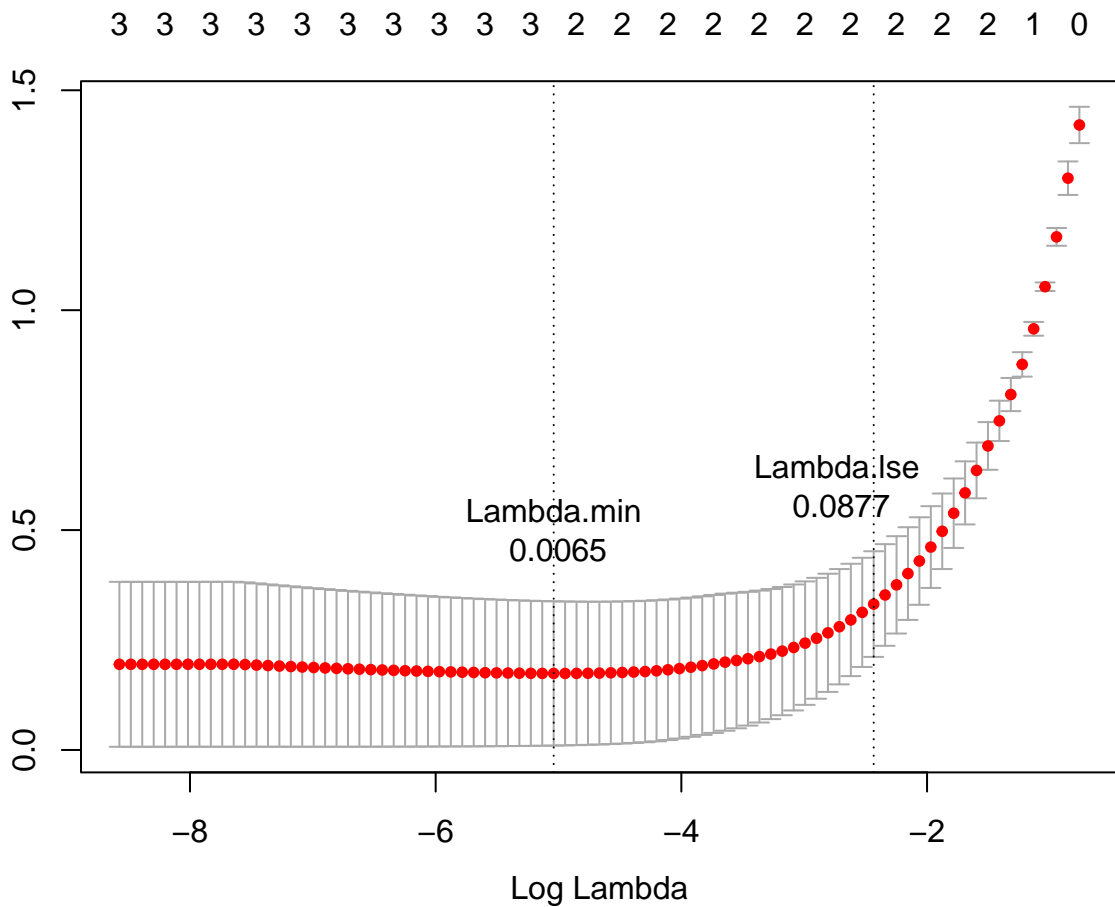

Supplement: Supplementary file 4 [file DataSheet1.zip › Raw data/06machine/fig01_lasso.Binomial.Deviance1.pdf]

Coefficients

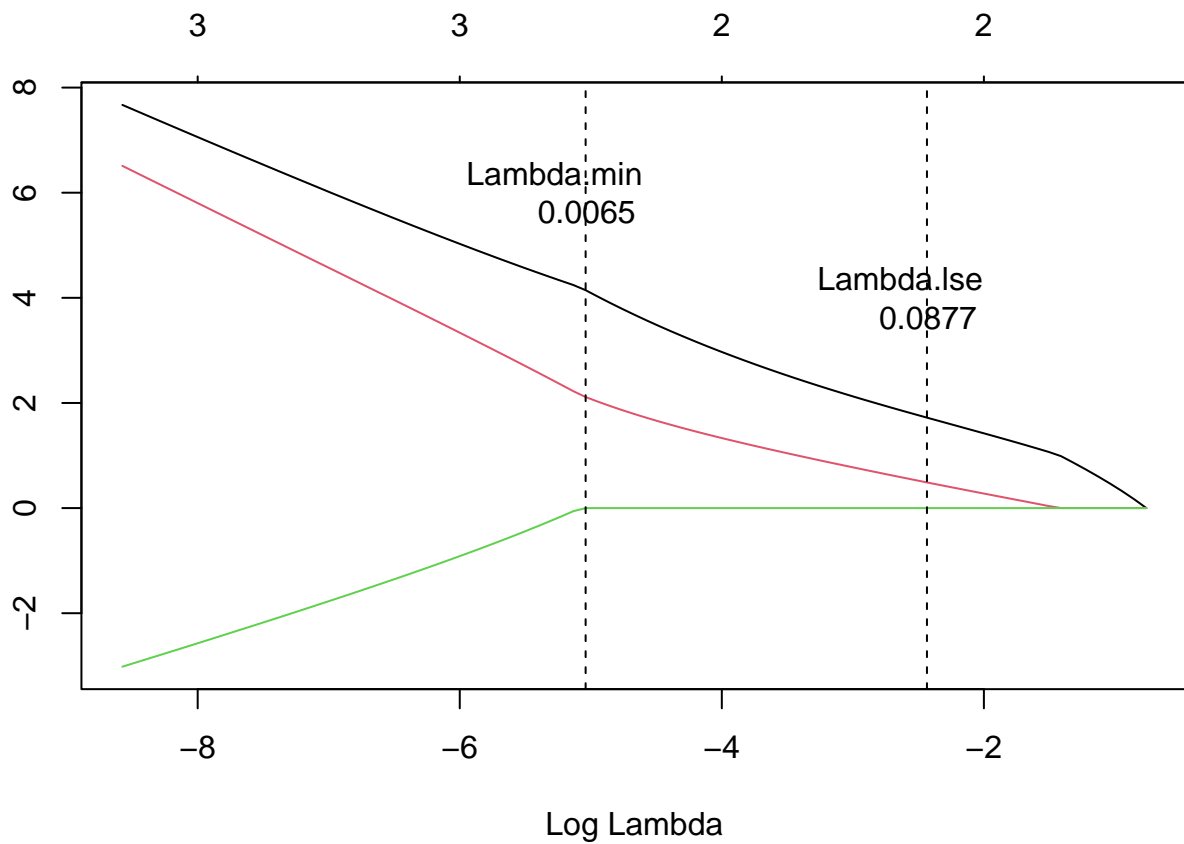

Supplement: Supplementary file 4 [file DataSheet1.zip › Raw data/06machine/fig02_lasso.voefficients.venalty.pdf]

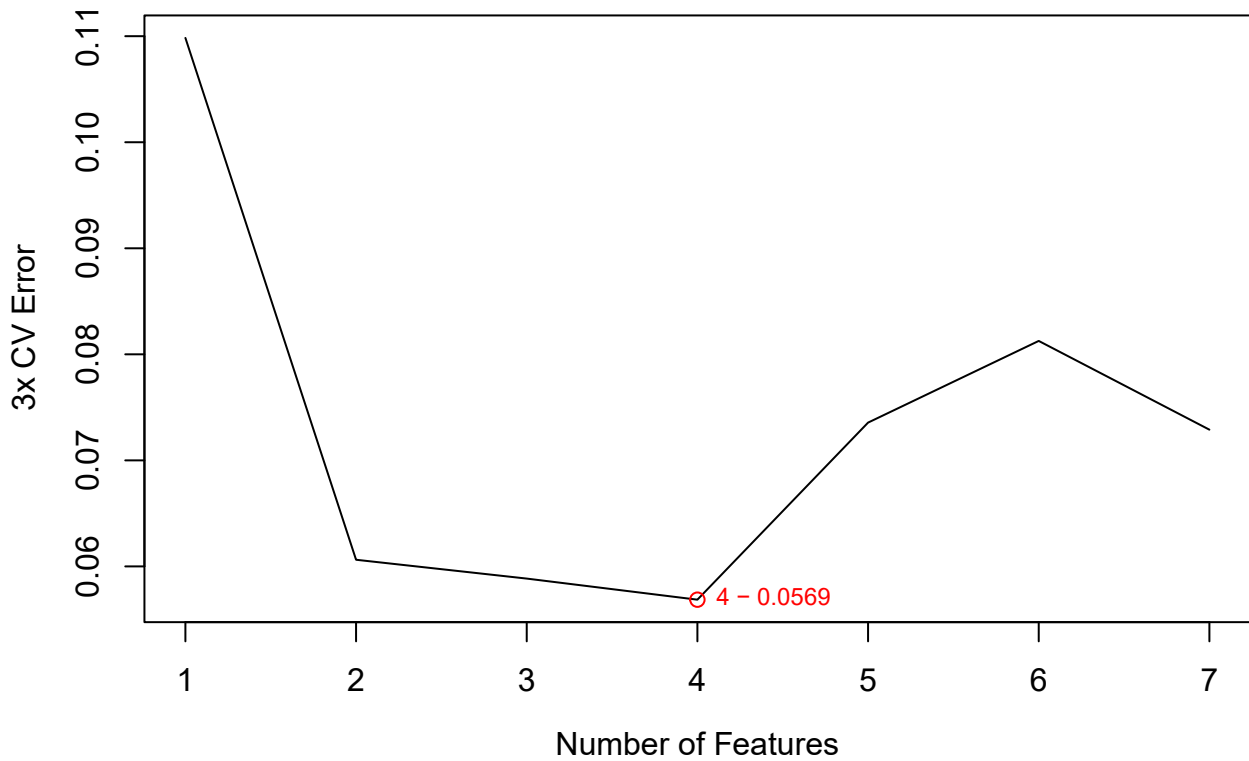

Supplement: Supplementary file 4 [file DataSheet1.zip › Raw data/06machine/fig03_svm_error.pdf]

Control EM

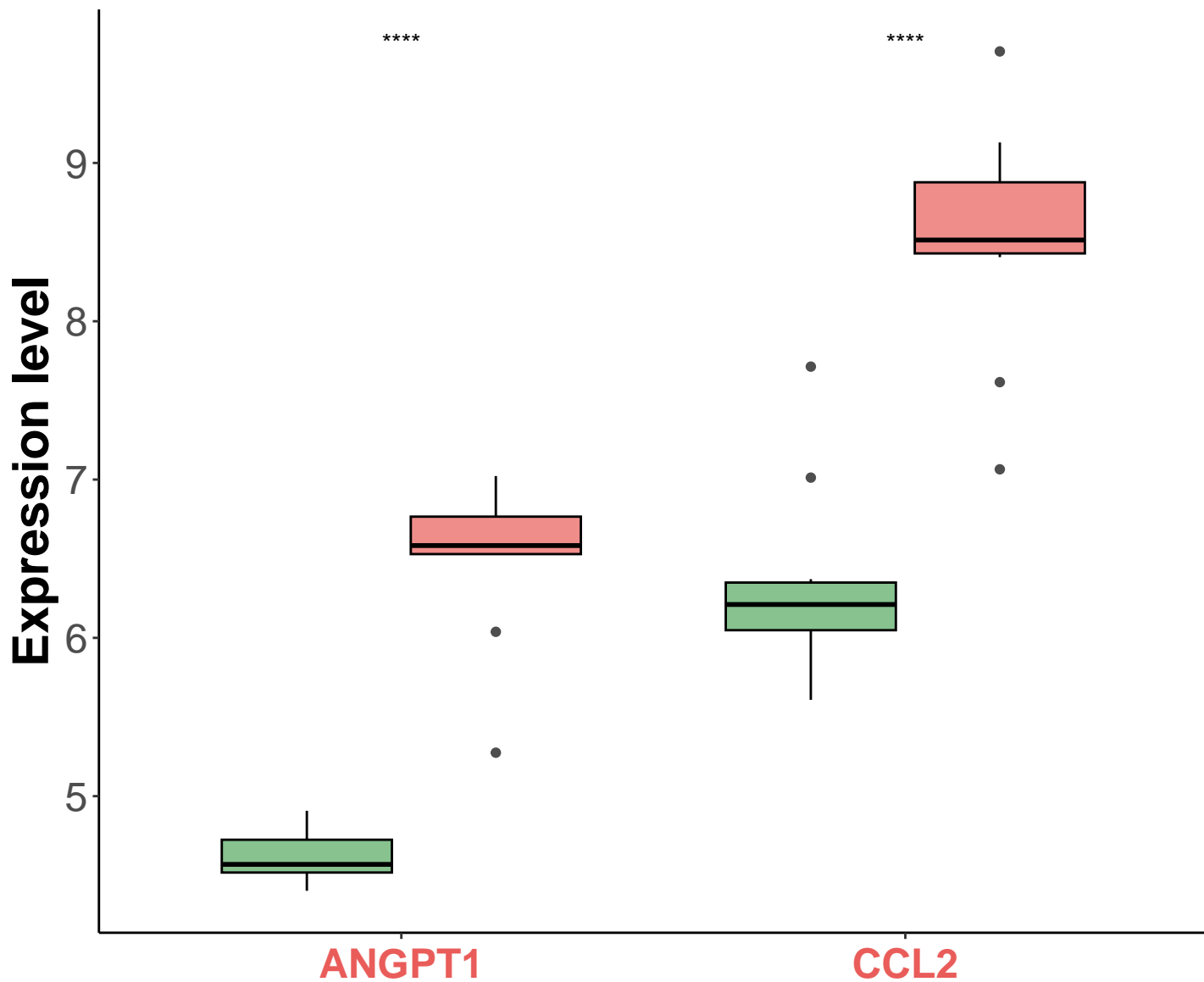

Supplement: Supplementary file 4 [file DataSheet1.zip › Raw data/07Expr&ROC/fig01_train_Expr_wilcox.pdf]

Control EM

Expression level

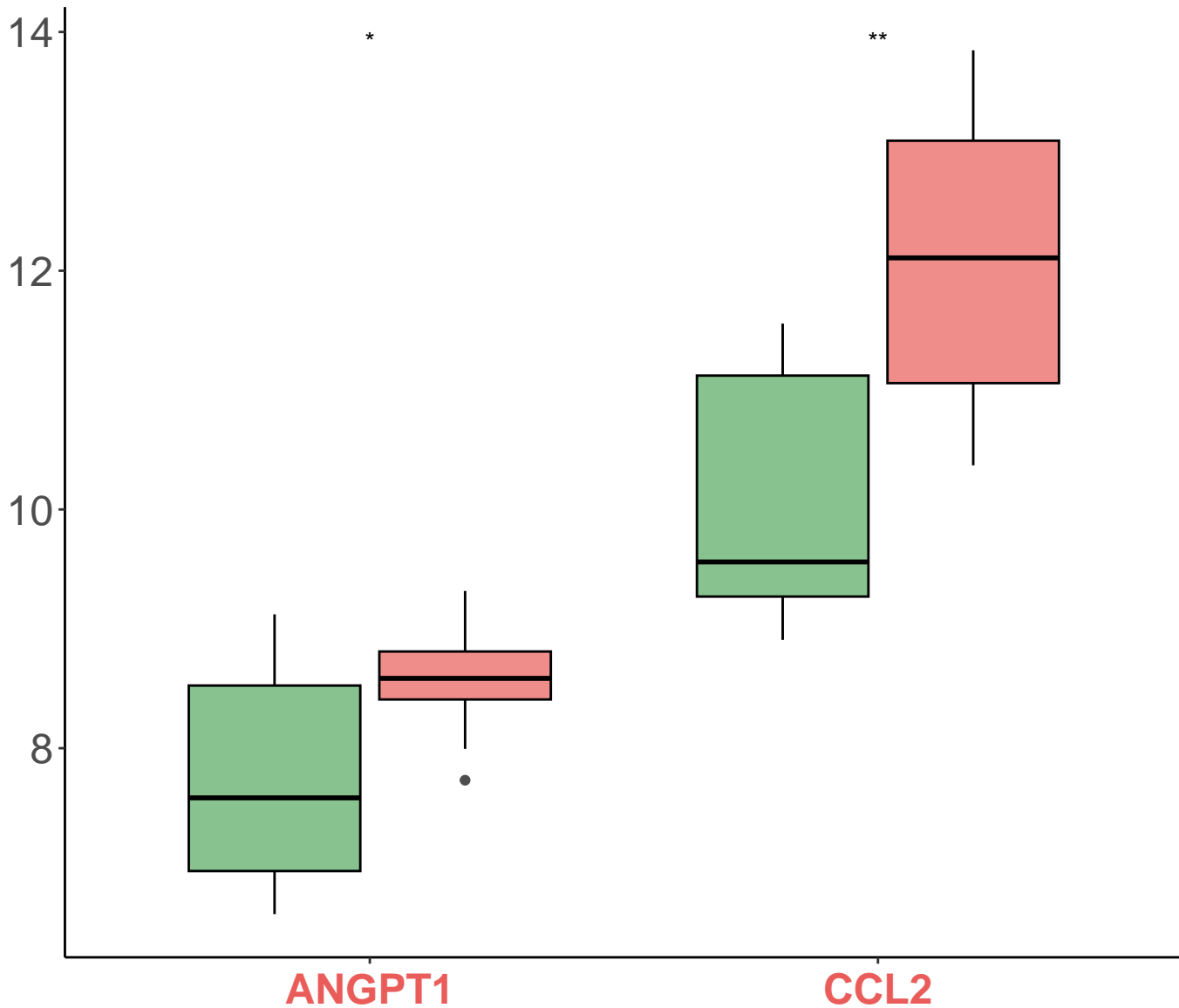

Supplement: Supplementary file 4 [file DataSheet1.zip › Raw data/07Expr&ROC/fig02_test_Expr_wilcox.pdf]

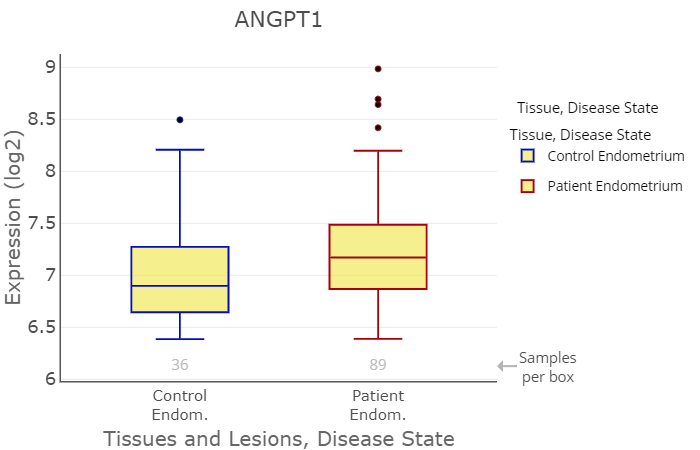

Supplement: Supplementary file 4 [file DataSheet1.zip › Raw data/07Expr&ROC/fig03_ANGPT1.png]

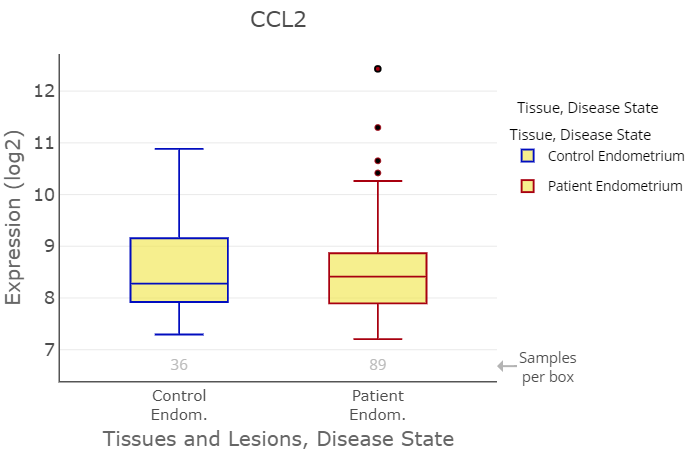

Supplement: Supplementary file 4 [file DataSheet1.zip › Raw data/07Expr&ROC/fig04_CCL2.png]

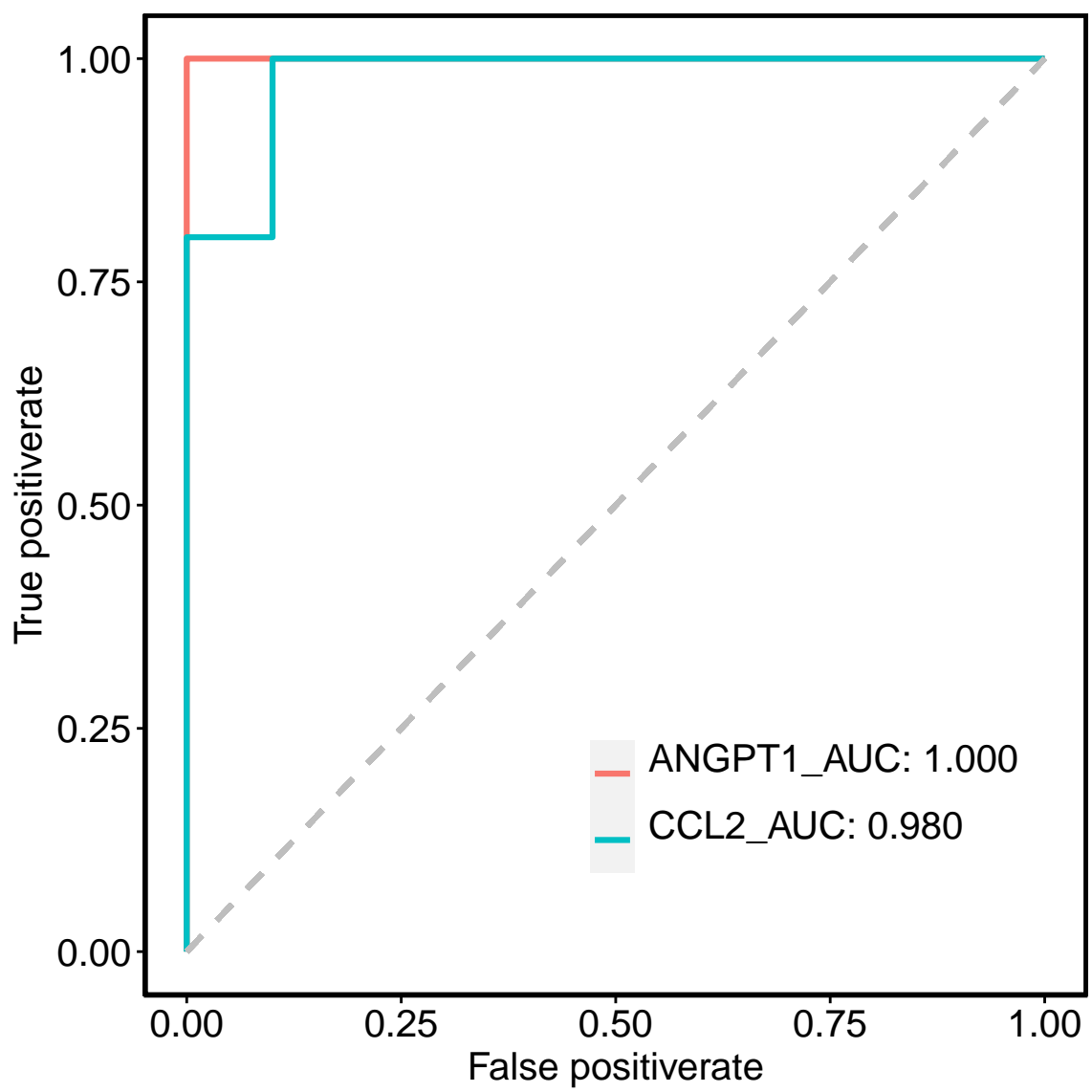

Supplement: Supplementary file 4 [file DataSheet1.zip › Raw data/07Expr&ROC/fig05_train_ROC.pdf]

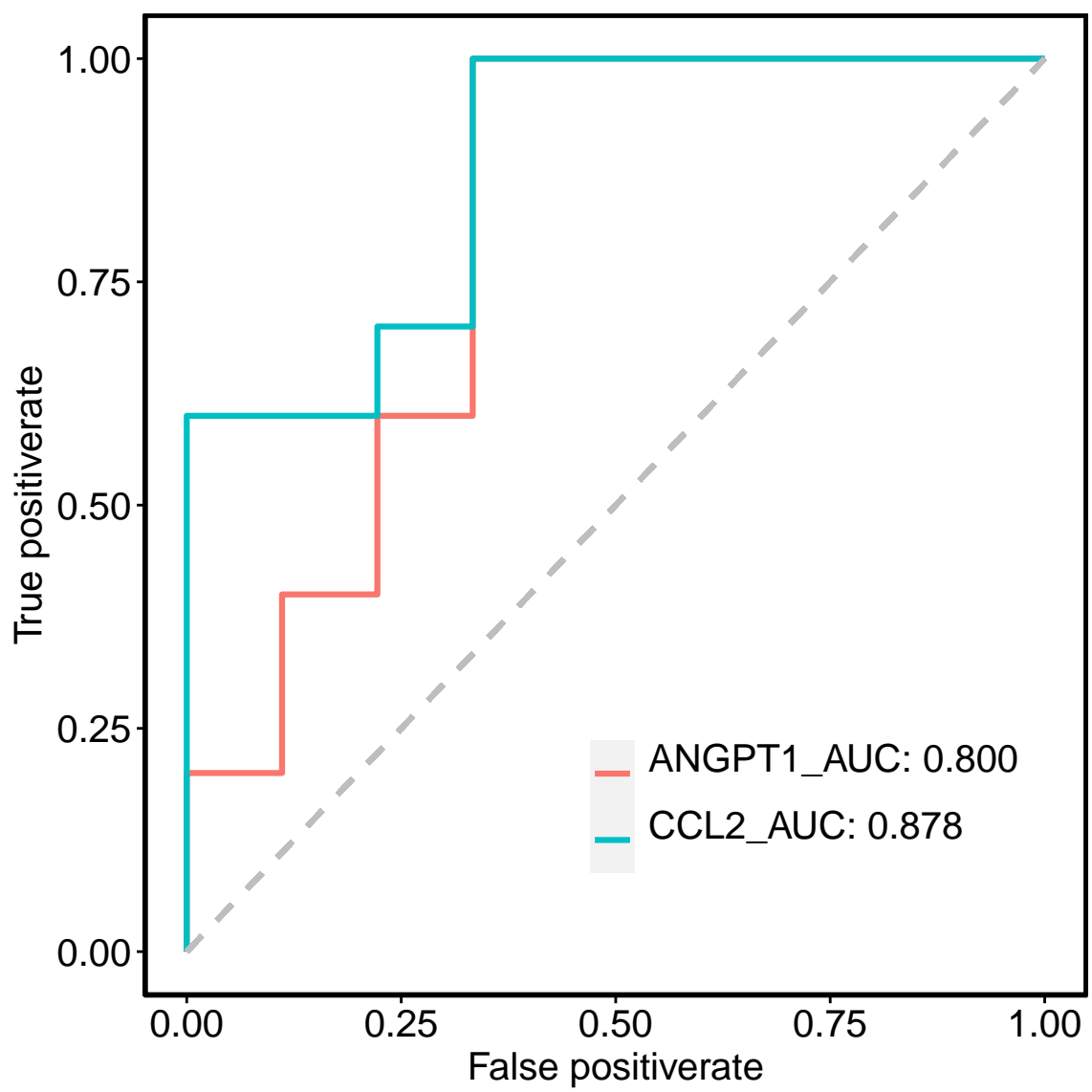

Supplement: Supplementary file 4 [file DataSheet1.zip › Raw data/07Expr&ROC/fig06_test_ROC.pdf]

Points

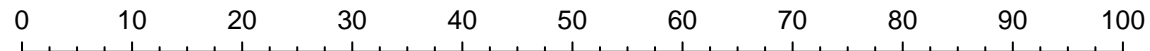

ANGPT1

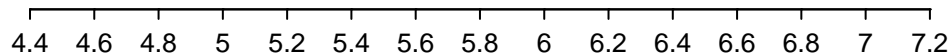

CCL2

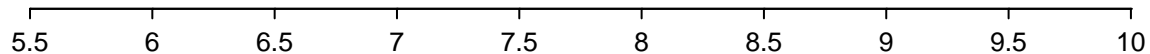

Total Points

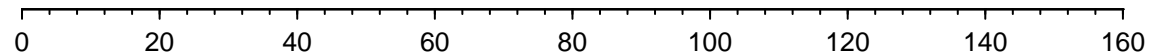

Risk of EM

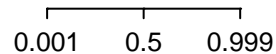

Supplement: Supplementary file 4 [file DataSheet1.zip › Raw data/08Nomogram/fig01_Gene_Nomogram.pdf]

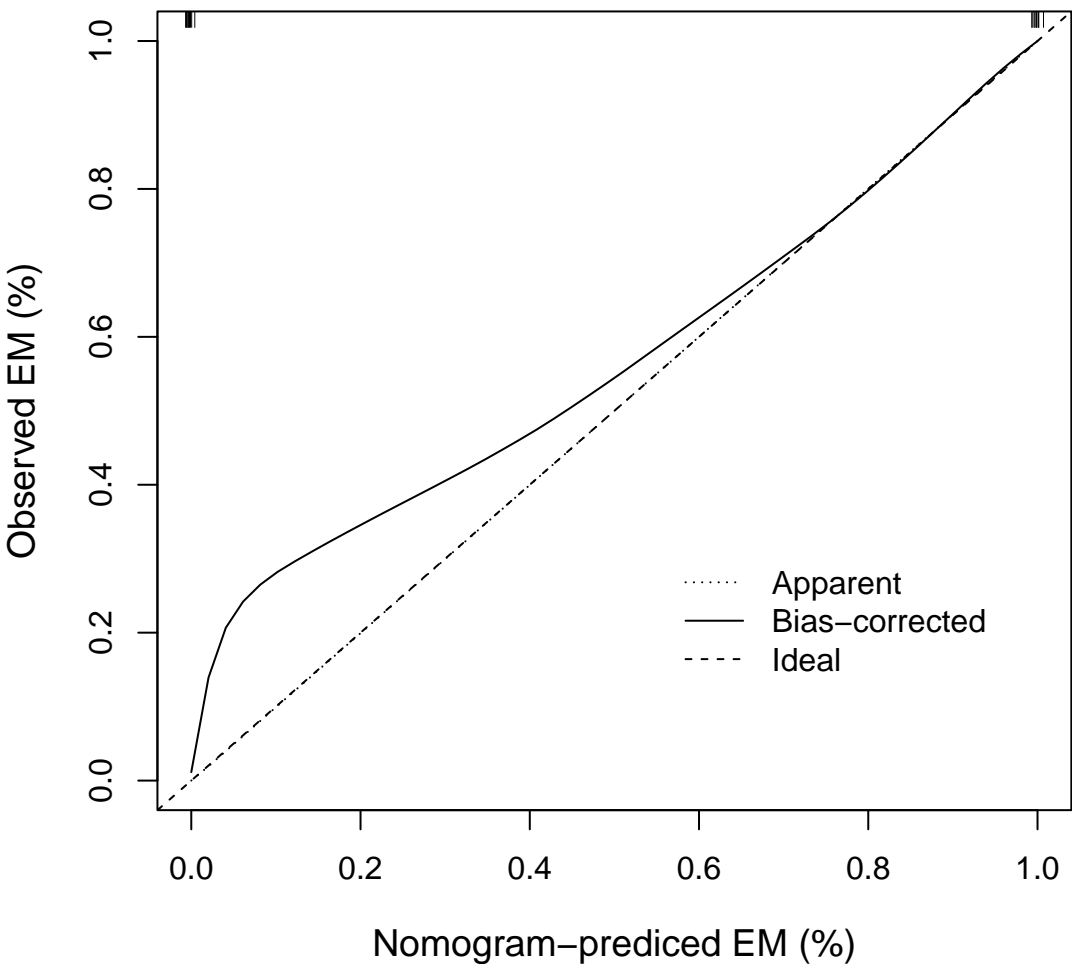

B= 1000 repetitions, boot

Mean absolute error=0.006 n=20

Supplement: Supplementary file 4 [file DataSheet1.zip › Raw data/08Nomogram/fig02_Nomogram_cal.pdf]

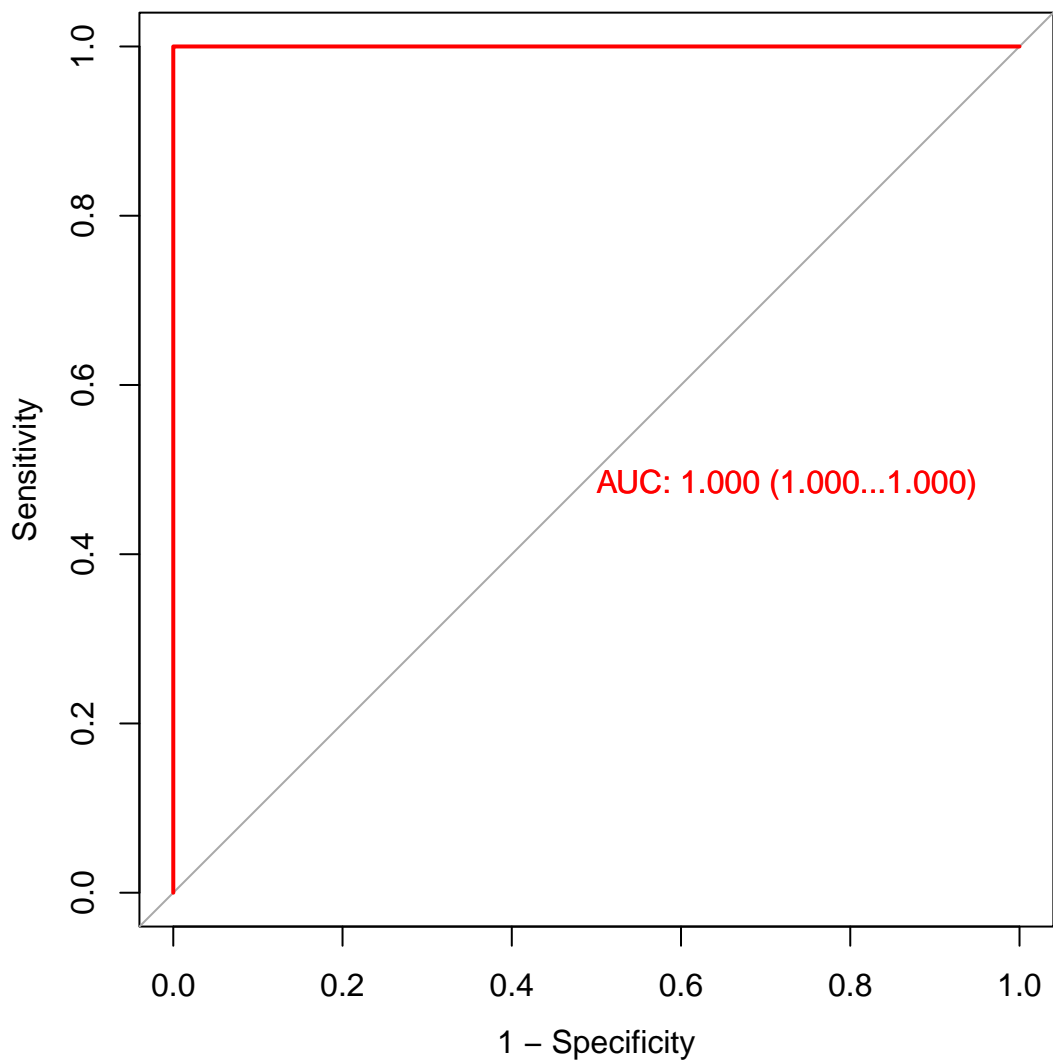

Supplement: Supplementary file 4 [file DataSheet1.zip › Raw data/08Nomogram/fig03_logistic_roc.pdf]

# ANGPT1

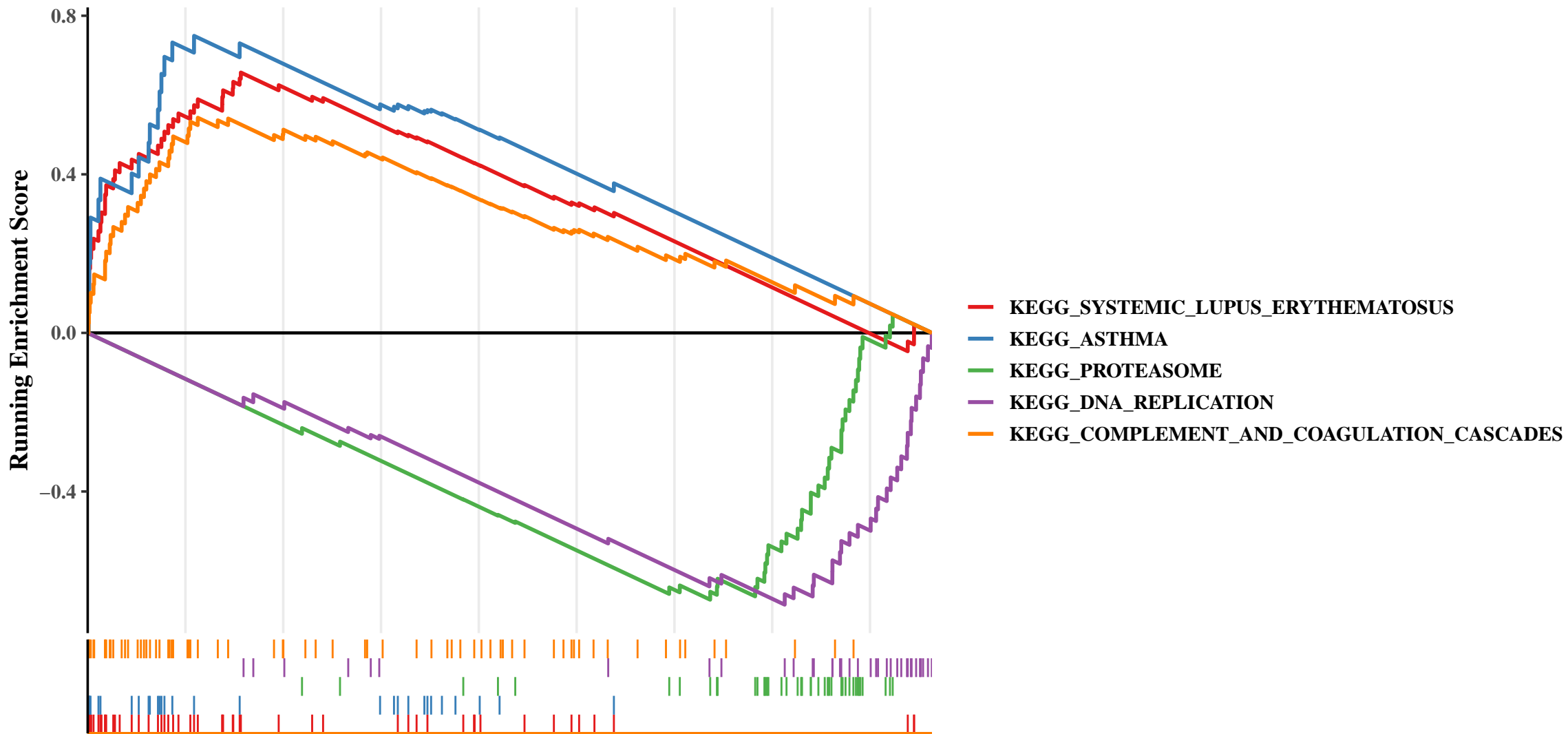

Supplement: Supplementary file 4 [file DataSheet1.zip › Raw data/09GSEA/fig01.ANGPT1_KEGG.pdf]

CCL2

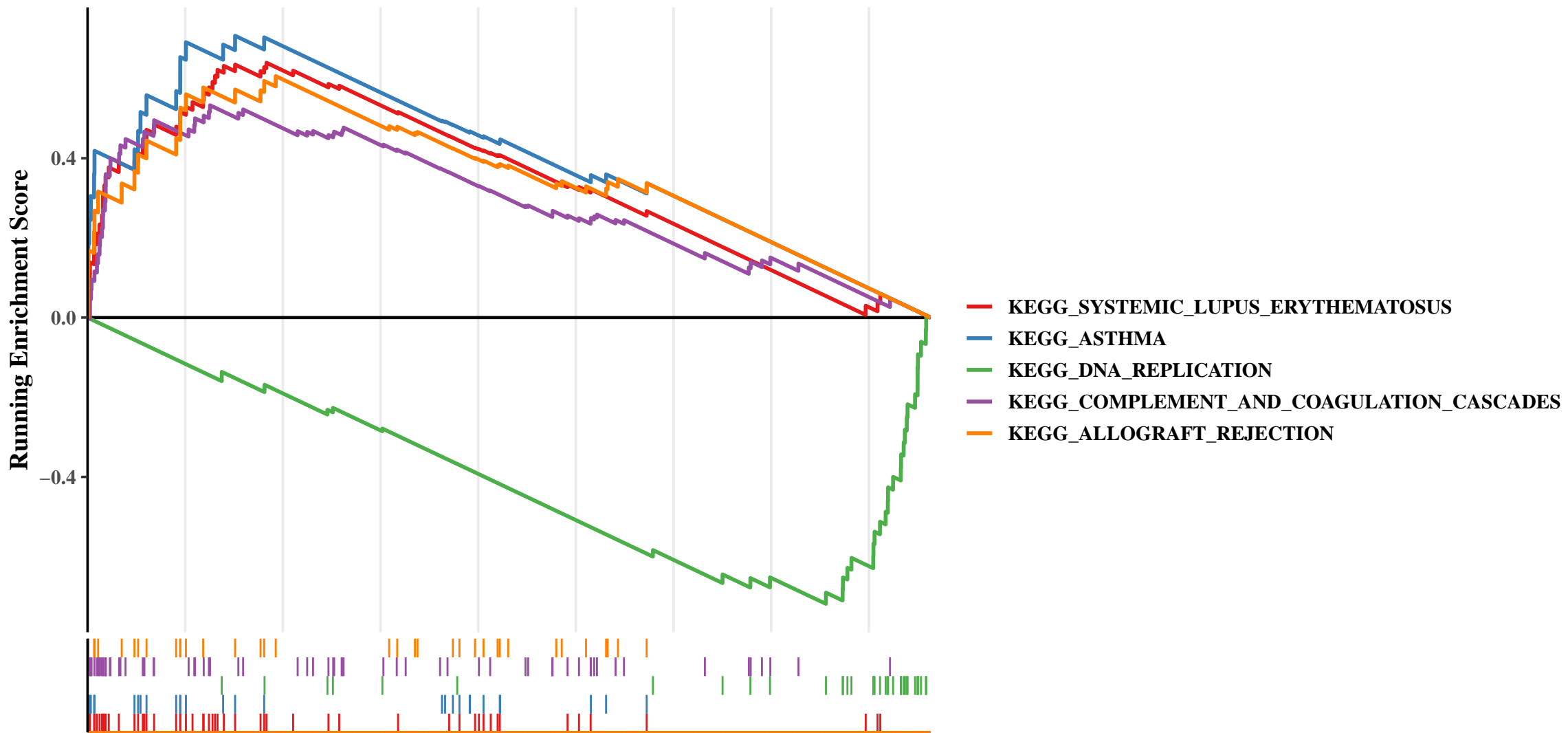

Supplement: Supplementary file 4 [file DataSheet1.zip › Raw data/09GSEA/fig02.CCL2_KEGG.pdf]

group Control EM

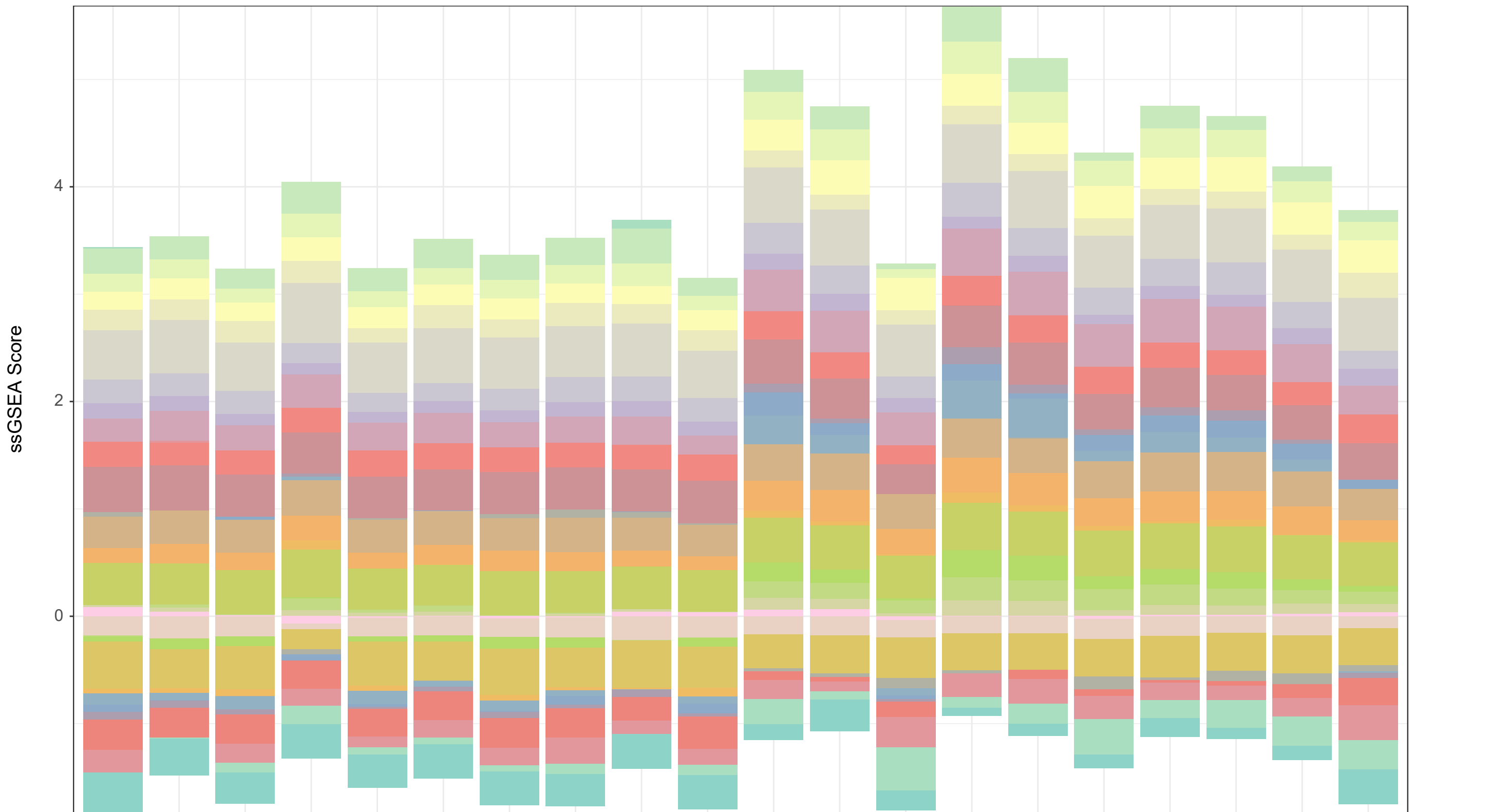

Cell Type

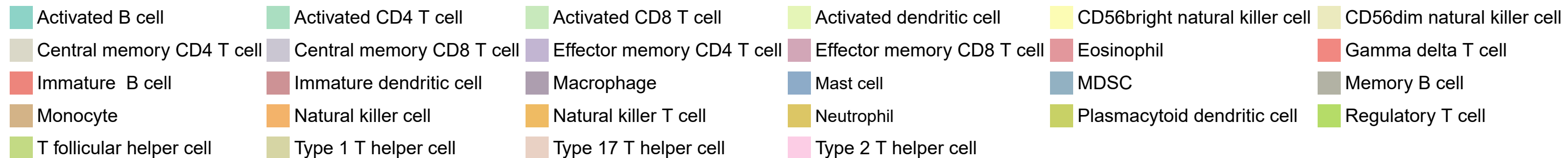

Supplement: Supplementary file 4 [file DataSheet1.zip › Raw data/13Immune/fig01_Immune_Cell_rate.pdf]

ssGSEA Score

Control EM

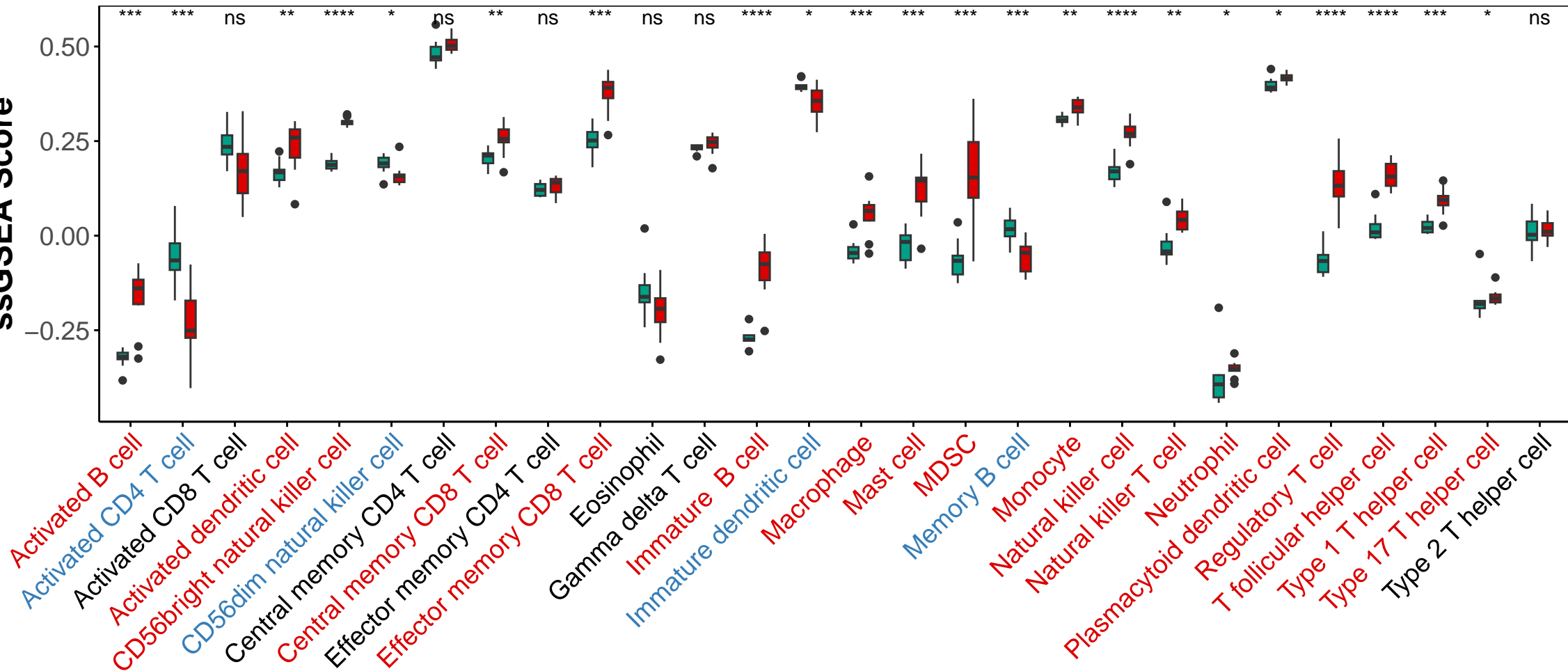

Supplement: Supplementary file 4 [file DataSheet1.zip › Raw data/13Immune/fig02_Immune_Box.pdf]

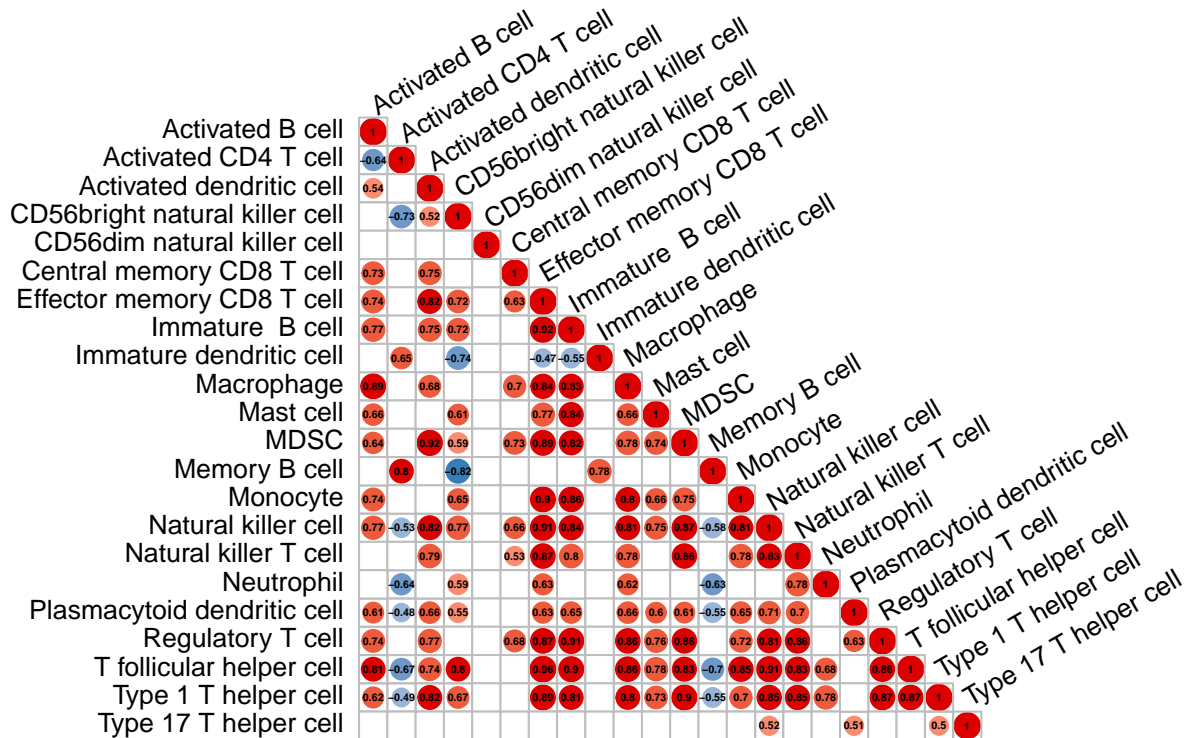

Supplement: Supplementary file 4 [file DataSheet1.zip › Raw data/13Immune/fig03_de_Immcell_cor.pdf]

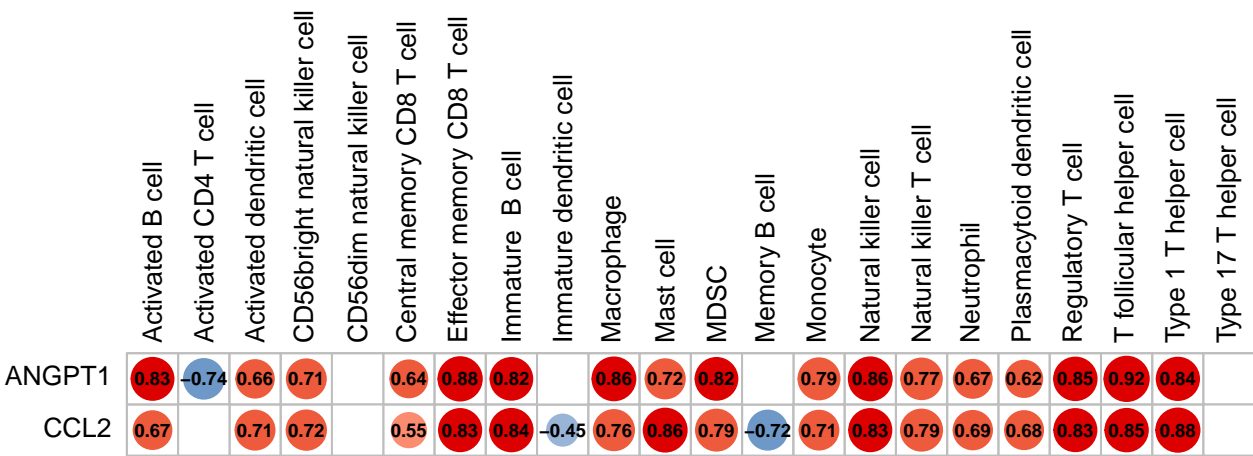

Supplement: Supplementary file 4 [file DataSheet1.zip › Raw data/13Immune/fig04_hub_de_Immcell_cor.pdf]

MET

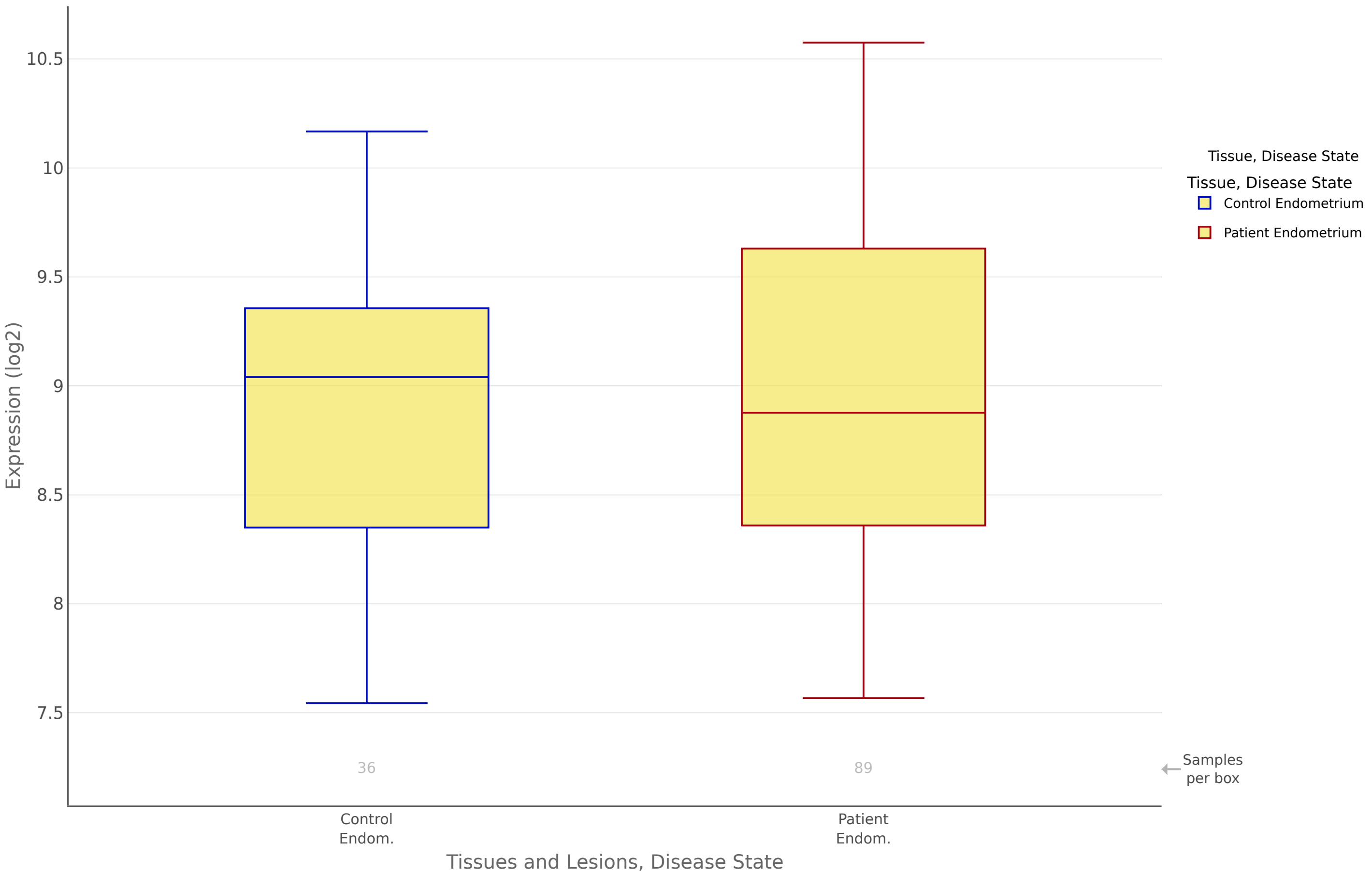

Supplement: Supplementary file 4 [file DataSheet1.zip › Raw data/MET.pdf]

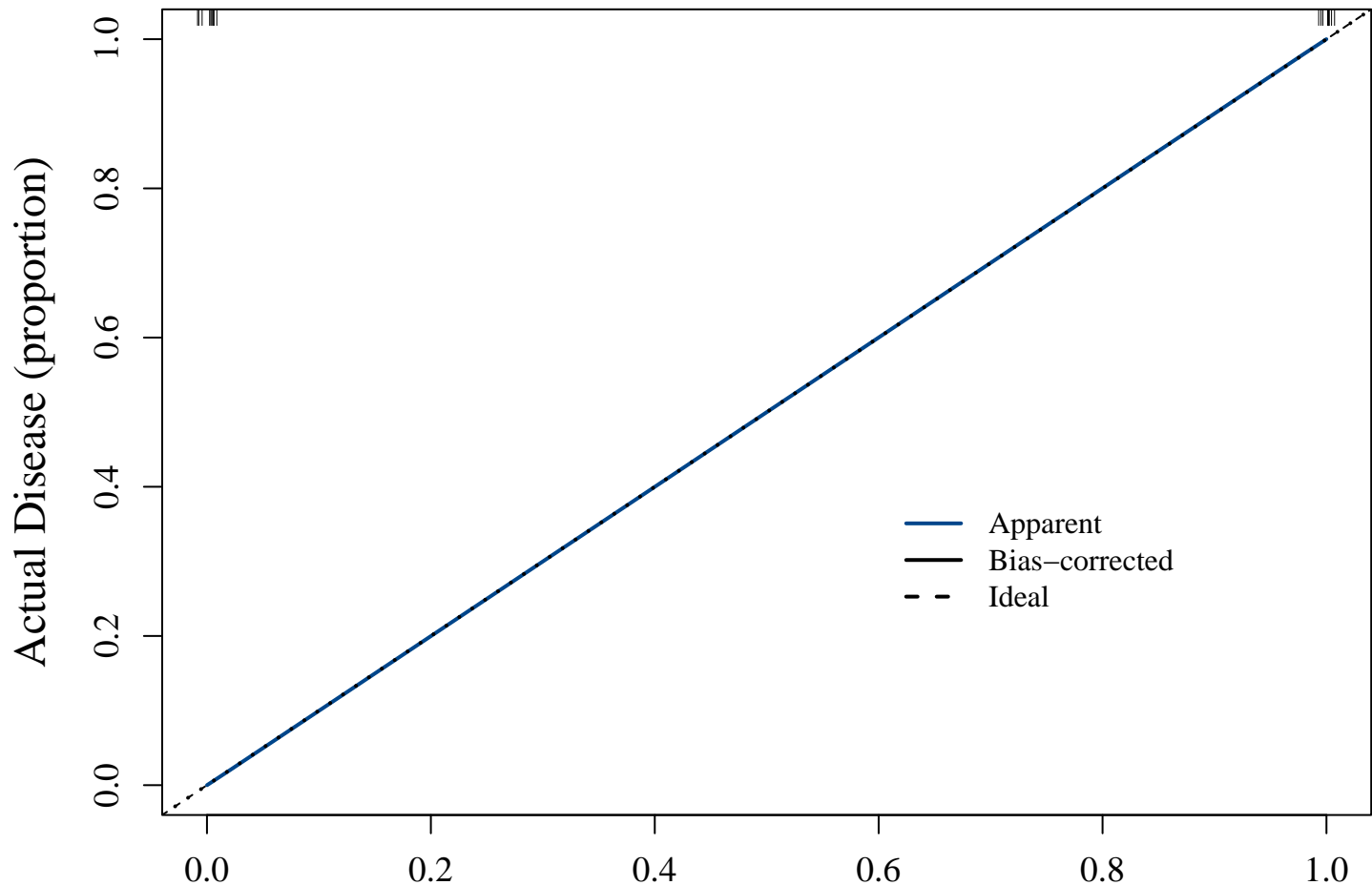

Nomogram-Predicted Probability of Disease risk

B= 30 repetitions, boot

Mean absolute error=0 n=20

Supplement: Supplementary file 4 [file DataSheet1.zip › Raw data/trian/02.calibrate.pdf]

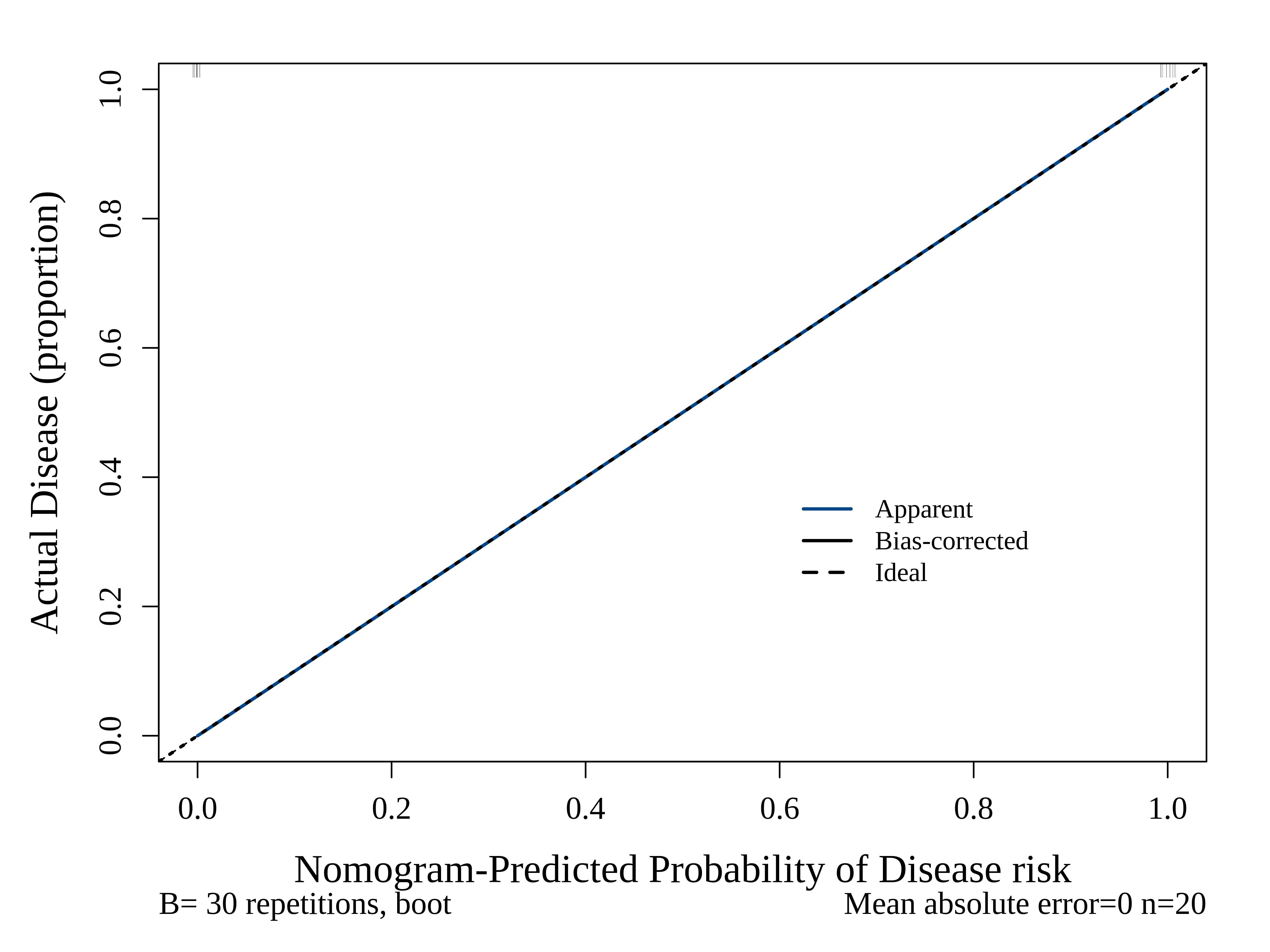

Supplement: Supplementary file 4 [file DataSheet1.zip › Raw data/trian/02.calibrate.png]

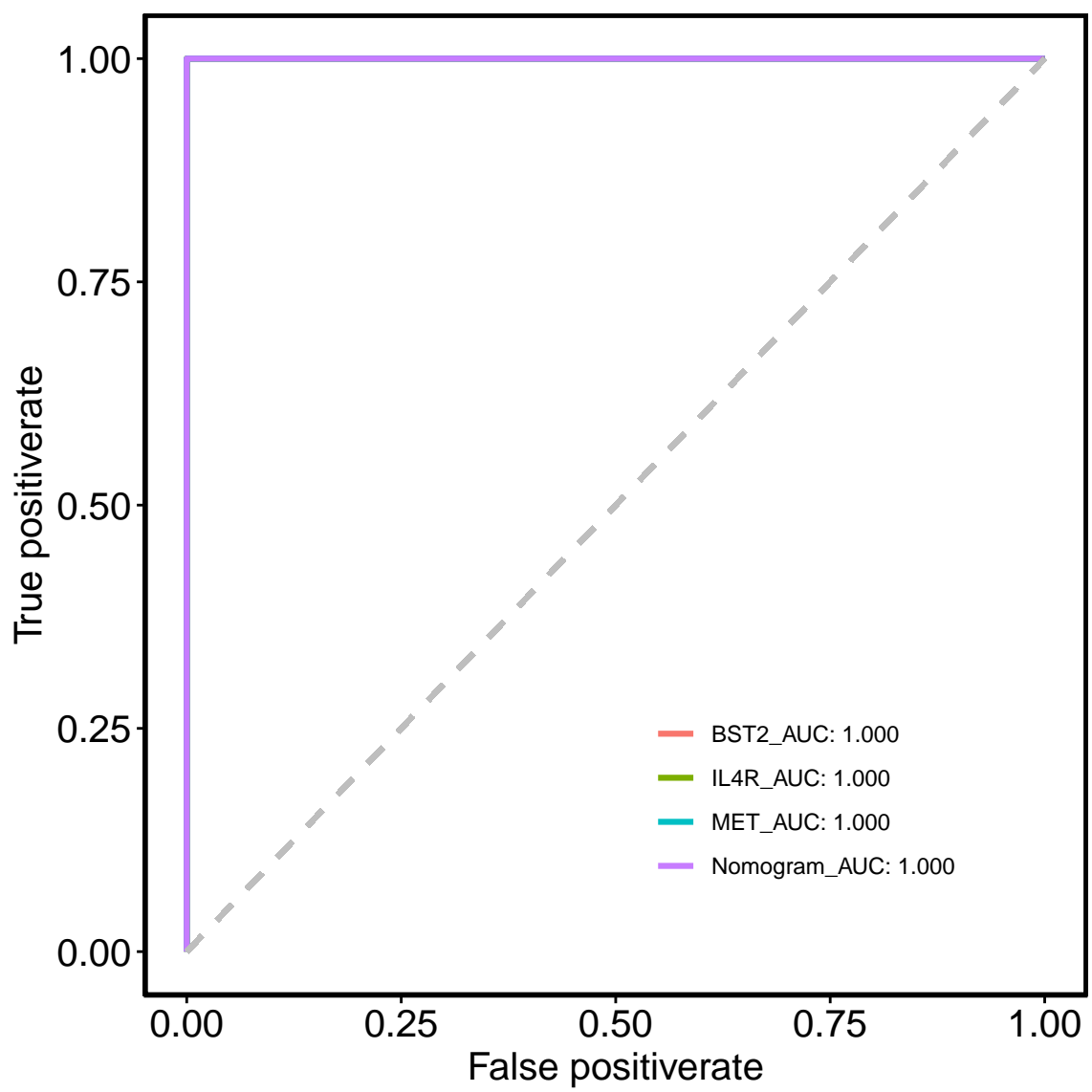

Supplement: Supplementary file 4 [file DataSheet1.zip › Raw data/trian/03.ROC.pdf]

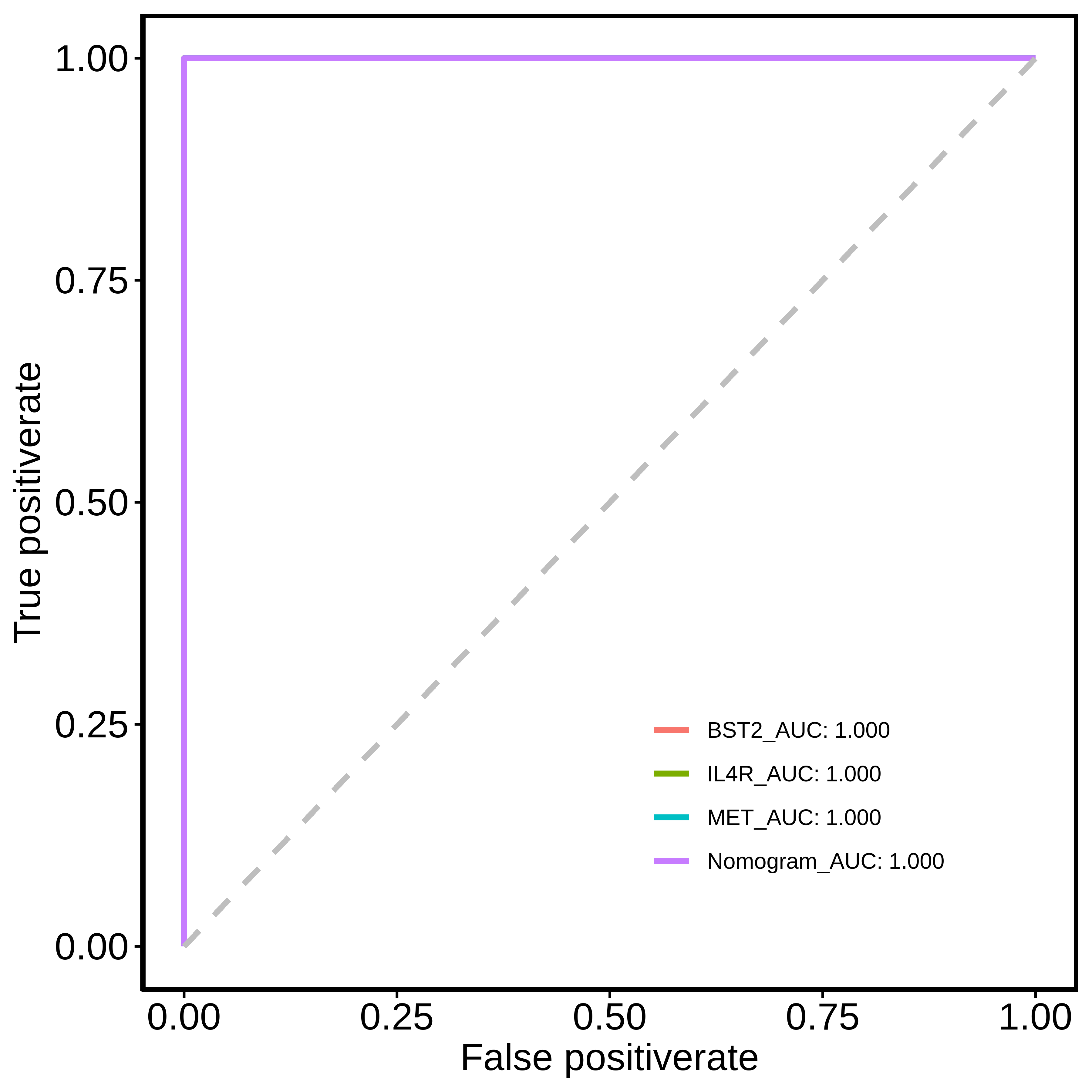

Supplement: Supplementary file 4 [file DataSheet1.zip › Raw data/trian/03.ROC.png]

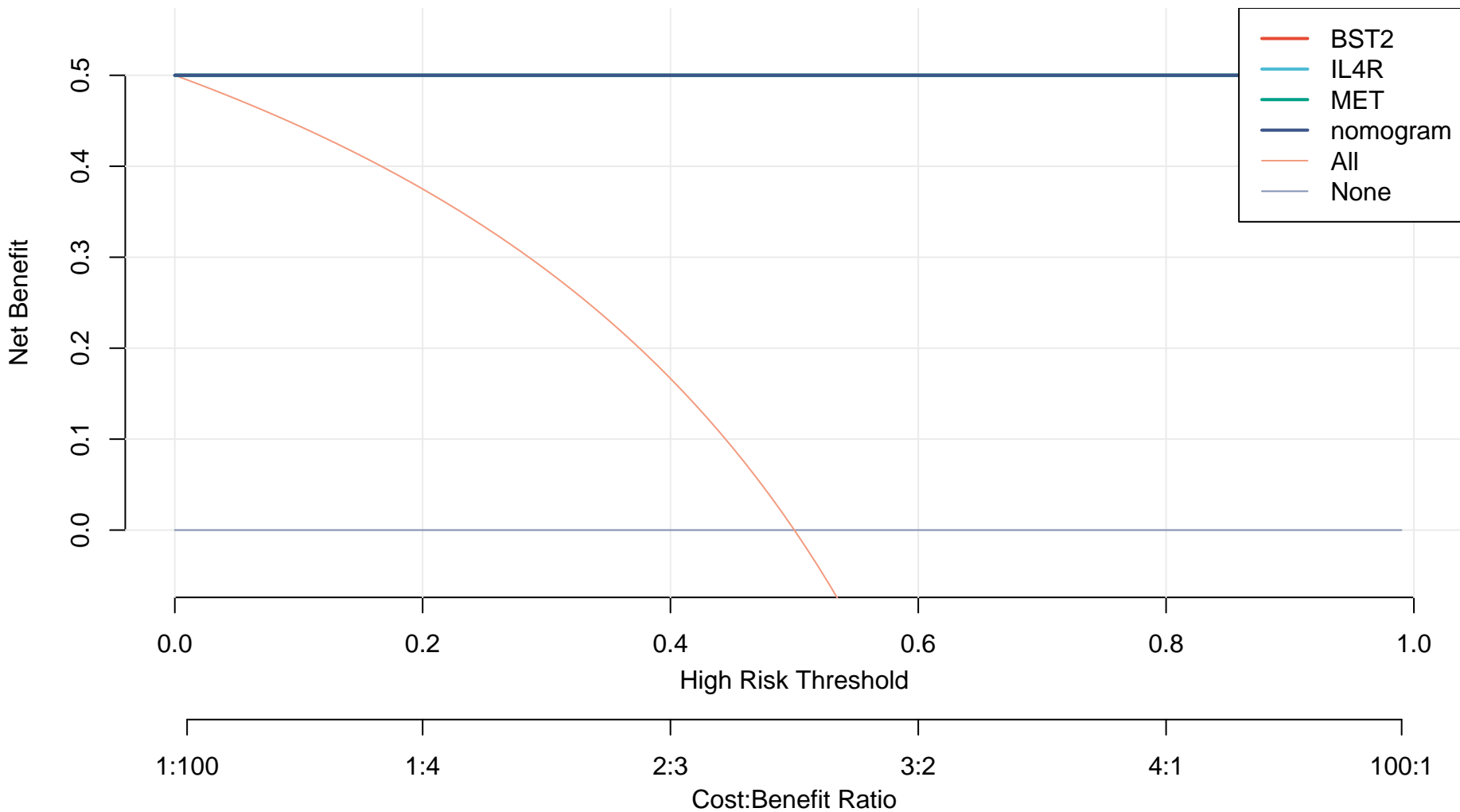

Supplement: Supplementary file 4 [file DataSheet1.zip › Raw data/trian/04.DCA.pdf]

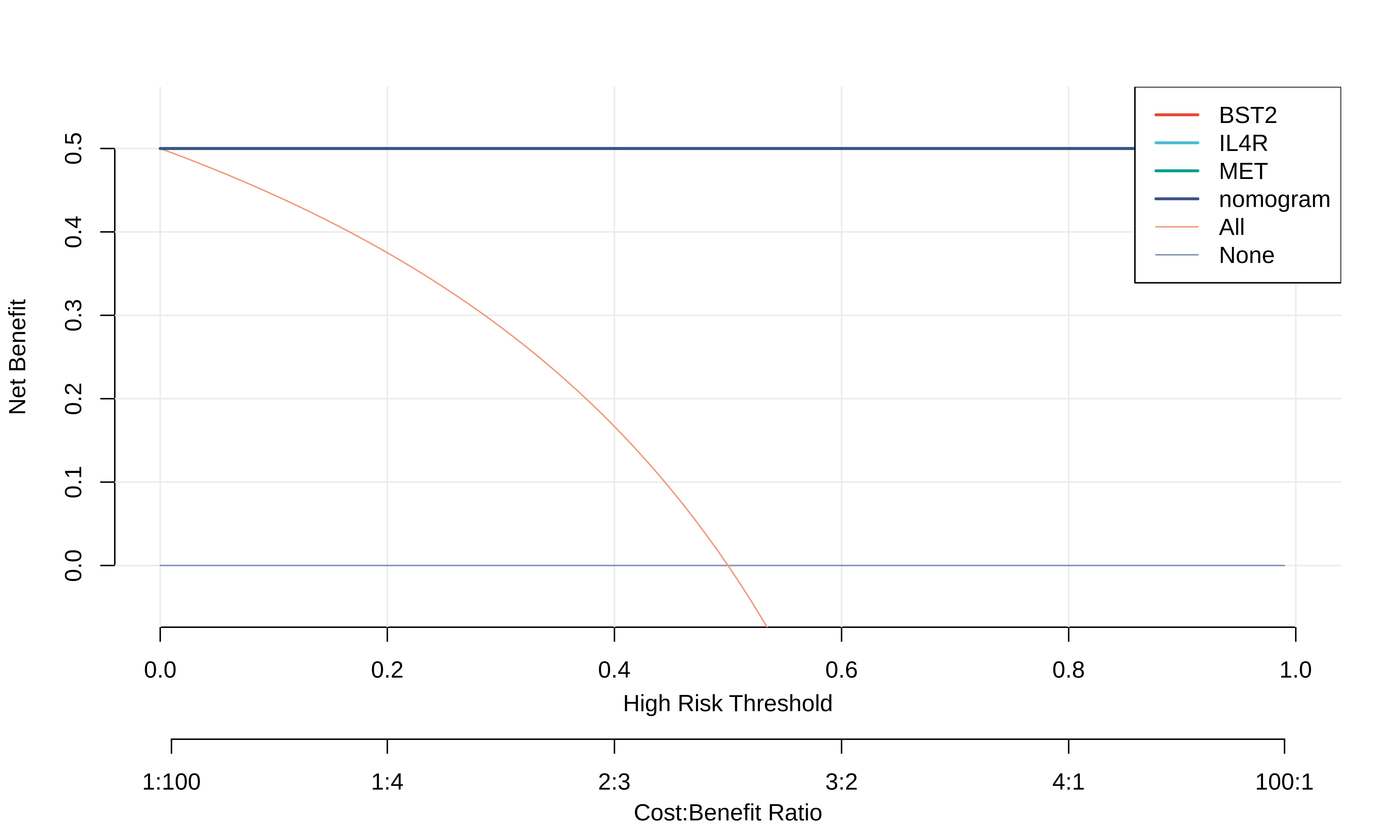

Supplement: Supplementary file 4 [file DataSheet1.zip › Raw data/trian/04.DCA.png]
