# Supplementary material for: Identification and validation of immune-related and inflammation-related genes in endometriosis
Source: Front Endocrinol (Lausanne). 2025 May 8;16:1545670. doi: 10.3389/fendo.2025.1545670 (PMC12095003; doi:10.3389/fendo.2025.1545670)
Supplement: Supplementary file 4 [file fig02_mdsPlot.pdf › Raw data/02Met/fig02_mdsPlot.pdf]

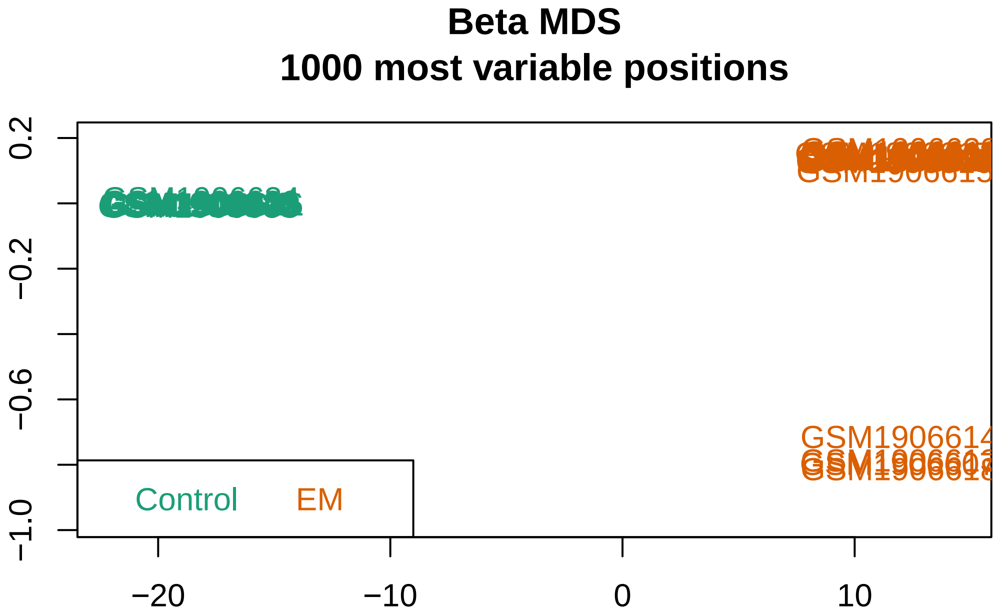
Beta MDS
1000 most variable positions
0.2
−0.2
−0.6
−1.0
−20
−10
0
10
GSM1906614
Control
EM
