# Supplementary material for: Identification and validation of immune-related and inflammation-related genes in endometriosis
Source: Front Endocrinol (Lausanne). 2025 May 8;16:1545670. doi: 10.3389/fendo.2025.1545670 (PMC12095003; doi:10.3389/fendo.2025.1545670)
Supplement: Supplementary file 4 [file DataSheet1.zip › Raw data/soft.doc]

| Program | Version | Reference/URL  Program information |
| --- | --- | --- |
| limma | 3.56.2 | Ritchie, M.E., Phipson, B., Wu, D., Hu, Y., Law, C.W., Shi, W.,  and Smyth, G.K. (2015). limma powers differential expression  analyses for RNA-sequencing and microarray studies. Nucleic  Acids Research 43(7), e47. |
| clusterProfiler | 4.8.2 | Tang, L Zhan, X Fu, S Liu, X Bo, and G Yu. clusterProfiler  4.0: A universal enrichment tool for interpreting omics data.  The Innovation. 2021, 2(3):100141. |
| e1071 | 1.7-13 | Statistics, Probability Theory Group (Formerly: E1071), TU  Wien. R package version 1.7-13, <https://CRAN.R-project.org/package=e1071>. |
| glmnet | 4.1-6 | Journal of Statistical Software, *33*(1), 1-22. doi:10.18637/jss.v033.i01  <https://doi.org/10.18637/jss.v033.i01>. |
| pROC | 1.18.4 | Müller (2011). pROC: an open-source package for R and S+ to analyze and compare ROC curves. BMC Bioinformatics, 12, p. 77. DOI: 10.1186/1471-2105-12-77 <http://www.biomedcentral.com/1471-2105/12/77/> |
| rms | 6.7-0 | Harrell Jr FE (2023). _rms: Regression Modeling Strategies. R package version  6.7-0, <https://CRAN.R-project.org/package=rms>. |
| psych | 2.3.6 | Psychometric, and Personality Research. Northwestern University, Evanston, Illinois. R package version 2.3.6, <https://CRAN.R-project.org/package=psych>. |
|  |  |  |
